# Hydrophobic alkyl-linked base-modified pyrimidine and 7-deazapurine 2′-deoxyribonucleoside phosphoramidites: synthesis and application in solid-phase synthesis of modified and hypermodified oligonucleotides

**DOI:** 10.1039/d5ra06147d

**Published:** 2025-12-08

**Authors:** Ivana Jestřábová, Lucie Bednárová, Lenka Poštová Slavětínská, Michal Hocek

**Affiliations:** a Institute of Organic Chemistry and Biochemistry, Czech Academy of Sciences Flemingovo nam. 2 CZ-16000 Prague 6 Czech Republic hocek@uochb.cas.cz; b Department of Organic Chemistry, Faculty of Science, Charles University Hlavova 8 CZ-12843 Prague 2 Czech Republic

## Abstract

A series of 2′-deoxyribonucleoside 3′-phosphoramidites bearing hydrophobic alkyl-linked modifications at nucleobases was synthesized, namely 5-phenylethyluracil, 5-pentylcytosine, 7-(indol-3-yl)ethyl-7-deazaadenine, and 7-isopentyl-7-deazaguanine derivatives. These nucleoside phosphoramidites were used for solid-phase synthesis of modified and hypermodified oligonucleotides containing up to fifteen modified nucleotides in a row. Their hybridization with complementary non-modified or modified oligonucleotides and thermal stability of the resulting DNA duplexes was studied using UV-vis denaturing experiments and CD spectroscopy. The results indicate that the partially modified hydrophobic DNA can still retain B-conformation, although with lower thermal stability. On the other hand, the hypermodified ONs containing all four modified nucleotides did not hybridize to duplexes likely due to formation of aggregates as indicated by dynamic light scattering measurement. This work expands the toolkit of chemically modified nucleotides for applications in functional nucleic acids or nucleic acid therapeutics, but also shows the scope and limitations of the use of hydrophobic nucleotides in hypermodified oligonucleotides and DNA.

## Abstract 2

Four nucleoside phosphoramidites bearing aliphatic or aromatic groups linked through flexible alkyl tether were synthesized and used for solid-phase oligonucleotide synthesis and for study of stability of duplexes.

## Graphical abstract

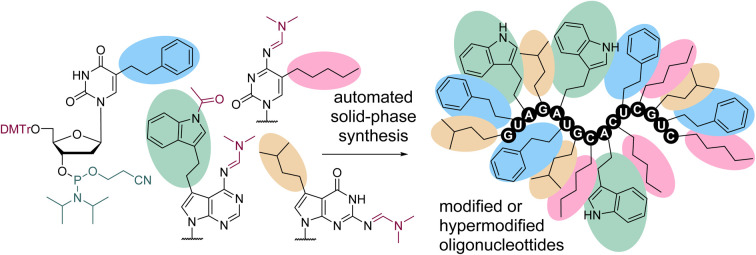

## Introduction

Chemically modified nucleic acids have become indispensable tools in chemical biology, diagnostics, therapeutic development, and material sciences.^1^ Synthetic oligonucleotides, bearing site-specific modifications on nucleobases, offer a powerful approach for tuning hybridization behaviour, enhancing binding specificity and affinity, and introducing new functionalities. This is particularly relevant in the context of aptamer selection and engineering,^2^ where introduction of one^3–10^ or two^11,12^ aromatic or hydrophobic moieties at nucleobases has led to significantly improved affinity and selectivity for protein targets, when compared to their unmodified counterparts.

The substitution of nucleobases with rigid alkynyl-linked substituents has been studied extensively. These modifications can be easily introduced through Pd-catalyzed Sobnogashira reactions of halogenated nucleosides with terminal alkynes and the resulting 5-alkynylpyrimidine^13–18^ and 7-alkynyl-7-deazapurine^19–21^ nucleotides were reported to stabilize (but in some cases destabilize^22^) DNA and RNA duplexes *via* enhanced π–π stacking interactions and increased hydrophobicity the and decrease the toxicity of antisense oligonucleotides (ASOs).^23^ On the other hand, flexible alkyl-linked modifications at the nucleobase have been studies less frequently mostly focusing on derivatives of 5-methylpyrimidines.^24–26^

In most of the above mentioned works, the studies have been limited to nucleic acids containing just one or two modified bases, whereas just a handful of scattered papers reported substitution on all four canonical nucleobases.^27,28^ In our lab, we have recently developed the general concept of hypermodified DNA and RNA where each and every nucleobase is bearing a different modification and their enzymatic synthesis using sets of all four modified (d)NTPs. In hypermodified DNA we reported examples containing four different hydrophobic modifications,^29,30^ anionic^31^ or cationic^32^ groups, sugars,^33^ amino acid-like functional groups^34^ and combinations of substituents from these classes. These hypermodified DNA that can contain up to 150 modified nucleotides in a row can still be sequenced and thus have potential in selection of aptamers and other functional nucleic acids. However, for these applications a phosphoramidite-based chemical synthesis^35^ of the hypermodified oligonucleotides will be mostly needed to provide these polymers in larger quantities. We have previously reported^36^ the design and synthesis of a complete set of base-modified 2′-deoxyribonucleoside 3′-phosphoramidites, each bearing alkyne-linked hydrophobic arylethyl or alkynyl substituents (analogous to the hydrophobic alkyne-linked dNTPs used for enzymatic synthesis of hypermodified DNA) and found an interesting effect that one or several alkynyl modifications slightly destabilize the DNA duplexes while the full modification at every nucleotide leads to stabilization of duplexes. To complement the previous study and extend the portfolio of hydrophobic modifications also to flexible alkyl substituents, we report here the synthesis of full set of all four 2′-deoxyribonucleoside 3′-phosphoramidites bearing four different hydrophobic alkyl or arylalkyl substituents and their use on the solid-phase synthesis of ONs.

## Results and discussion

### Synthesis of modified nucleoside phosphoramidites

**Fig. 1:**
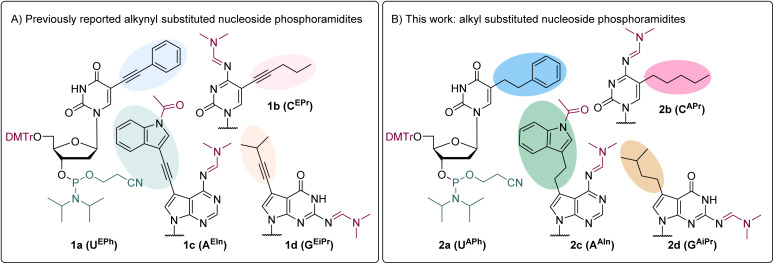
(A): Previously reported series with modifications linked to the nucleobase *via* ethynyl linker; (B): new series with ethyl linker between the modification and the nucleobase.

The set of modified nucleoside phosphoramidite building blocks was designed based on previously reported alkynyl-linked phosphoramidites (1a–1d)^36^ and on the set of modified dNTPs^29^ used for the enzymatic synthesis of hypermodified hydrophobic DNA (Fig. 1A). Hence the new target nucleoside phoshoramidites were derived from 5-phenylethyl-2′-deoxyuridine (2a), 5-pent-1-yl-2′-deoxycytidine (2b), 7-(indol-3-yl)ethyl-7-deaza-2′-deoxyadenosine (2c) and 7-isopentyl-7-deaza-2′-deoxyguanosine (2d) (Fig. 1B), where the substituents resemble and mimic side chains of amino acid residues phenylalanine (Phe), methionine (Met), tryptophan (Trp), and valine (Val), respectively.

**Scheme 1:**
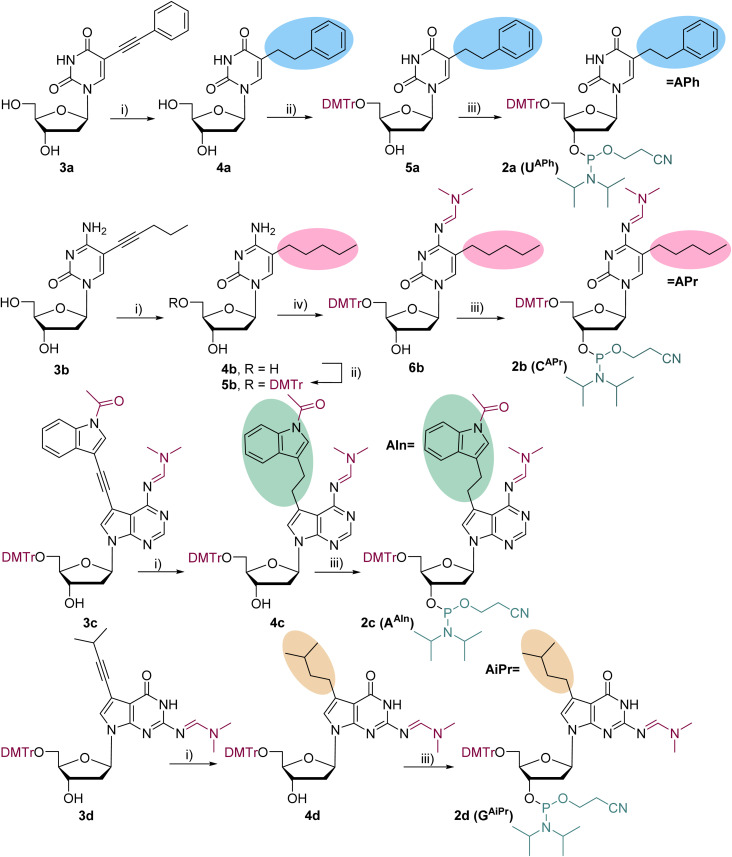
Reagents and conditions: (i) H_2_, 10% Pd/C, MeOH, iPrOH (in case of 3c and 3d), RT, 3 h (3a 90%, 3b 84%, 4c 77%, 4d 89%); (ii) DMTrCl (1.2 equiv.), DMAP (0.1 equiv.), pyridine (dry), RT, overnight (5a 77%, 5b 69%); (iii) 2-cyanoethyl-*N*,*N*-diisopropylchlorophosphoramidite (1.2 equiv.), DIPEA (2.5 equiv.), DCM (dry), 0 °C to RT, 1.5 h (2a 65%, 2b 54%, 2c 41%, 2d 57%); (iv) DMF-DMA (14 equiv.), DMF (dry), 40 °C, under Ar, 4 h (6b 92%).

The synthesis of modified pyrimidine nucleosides consisted of attaching the modifications through Sonogashira cross-coupling to the iodo-nucleoside according to the previous work,^36^ followed by hydrogenation of the ethynyl to the ethyl linker, introduction of protecting groups and and attachment of the phosphoramidite function (Scheme 1, see details in SI). The synthesis of functionalized pyrimidine nucleosides commenced with the reduction^29^ of the ethynyl linker to ethyl in compound 3a and 3b (prepared according to ref. 36) by H_2_ with 10% Pd/C (0.1 equiv for 3a and 0.3 equiv. for 3b) in MeOH. The reaction afforded the alkyl-linked nucleosides 4a and 4b in good yields of 90% and 84%, respectively. The 5′-hydroxyl group was then selectively protected using dimethoxytrityl chloride (DMTrCl) in the presence of *N,N*-dimethylaminopyridine (DMAP),^37^ resulting in DMTr-protected nucleosides 5a (77%) and 5b (69%). Subsequently, the exocyclic amino group of modified 2′-deoxycytidine 5b as a dimethylformamidine derivative by treatment with *N*,*N*-dimethylformamide dimethylacetal (DMF-DMA) in DMF^38^ at 40 °C resulting in compound 6b in a good yield (92%). Finally, coupling of 5a and 6b with 2-cyanoethyl-*N*,*N*-diisopropylchlorophosphoramidite^35^ in the presence of *N*,*N*-diisopropylethylamine (DIPEA) led to fully protected nucleoside phosphoramidites 2a (65%) and 2b (54%).

For the 7-substiututed 2′-deoxy-7-deazapurine nucleoside phosphoramidites, a different strategy was employed. First, the protecting groups were first installed, followed by attachment of the hydrophobic alkyne moiety, reduction of the alkyne linker, and conversion of the modified nucleosides to the corresponding phosphoramidites. Previously,^36^ for the introduction of indolylethyl moiety, we used 1-acetyl-3-(trimethylsilyl)ethynylindole, which required TEA·3HF for *in situ* cleavage of the TMS during the coupling reaction. Since we were not able to scale up the reaction under such conditions, we deprotected the trimethylsilyl from the indole moiety beforehand and used it as such in the Sonogashira cross-coupling. Therefore, we synthesized known 1-acetyl-3-ethynylindole^39^ which was then used in the Sonogashira reaction with protected 7-iodo-2′-deoxy-7-deazaadenosine 7c (see details in SI).

Hydrogenation of protected and functionalized nucleosides 3c and 3d by H_2_ with 10% Pd/C in MeOH resulted in compound 4c (77%) and 4d (89%), however, we needed to use large amount (1 equiv.) of the palladium catalyst to be able to perform the reduction in case of 3c (0.2 equiv. in case of 3d). Final reactions with 2-cyanoethyl-*N*,*N*-diisopropylchlorophosphoramidite, under the conditions mentioned above, resulted in target fully protected nucleoside phosphoramidites 2c (41%) and 2d (57%), respectively.

### Solid-phase synthesis of modified oligonucleotides

**Scheme 2:**
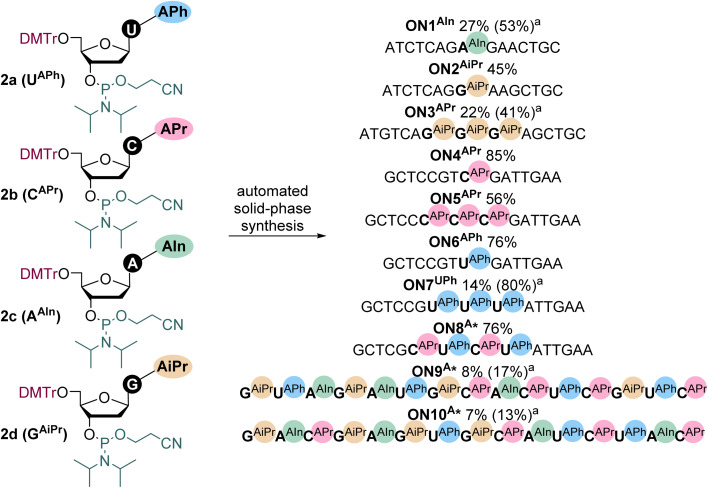
Solid-phase synthesis of the partially and hyper-modified oligonucleotides with their yields (a) HPLC yields given in parenthesis in case of lower isolated yield).

The modified nucleoside phosphoramidites 2a-2d were used for the automated synthesis of partially and fully modified oligonucleotides (ONs). We designed ten target sequences containing one or three modified nucleotides in the central part of the chain, combination of two different modified nucleotides and then fully modified complementary sequences containing combinations of all four modified nucleotides, corresponding to those we previously published^36^ in the alkyne-linked series (for sequences see Scheme 2 or Table S2 in SI). Since the final amount of the nucleoside phosphoramidite 2c was not sufficient for all types of ONs to be synthesized, oligonucleotides containing three 2c were neglected (Scheme 2), but we still synthesized the hyper-modified sequences (ON9^A^* and ON10^A^*). The ONs were synthesized using standard synthesis protocol for solid-phase synthesis employing the phosphoramidite method. The partially modified ON1^AIn^–ON8^A^* were synthesized on standard solid-phase columns with one nucleoside attached at the 3′ end, whereas the hyper-modified ON9^A^* and ON10^A^* were synthesized on the universal solid-phase columns, with no nucleoside on the solid phase support. The synthetic scale was set to 1 µmole using the 0.1 M concentration of all the phosphoramidites. The coupling duration for the natural phosphoramidites was 1 minute 30 seconds while for the modified phosphoramidites the coupling time was increased to 6 minutes to elevate the probability of successful incorporation. After the synthesis, the cleavage of the ONs from the solid phase was performed by aqueous ammonia, followed by deprotection of the protecting groups by heating the solution to 65 °C for 6 h. Subsequently, the HPLC purification of the ONs was performed, followed by the mass spectrometry characterization and purity control by UHPLC-MS (see details in SI). Concentration was approximately measured by nanodrop and precise concentration and yield determination was done by the elemental analysis of phosphorus content.

All the partially modified (ON1^AIn^–ON8^A^*) and fully modified ONs (ON9^A^*, ON10^A^*) were successfully synthesized, purified and characterized (see details in SI). According to the retention times obtained during HPLC purification on a C18 column (Table S1 in SI), the relative hydrophobicity of the resulting partially modified ONs can be estimated. Singly modified ONs show the lowest hydrophobicity from the set (ON4^APr^ < ON6^APh^ < ON2^AiPr^ < ON1^AIn^), indicating pentyl-C modification is the least hydrophobic and indole-modified A the highest. Generally, increasing the number of incorporated modifications further enhances hydrophobicity, as is reflected in the trend ON5^APr^ (3 mod.) < ON7^APh^ (3 mod.) < ON3^AiPr^ (3 mod.) < ON8A* (4 mod.). However, the isolation of the hyper-modified strands (ON9^A^*, ON10^A^*) was more difficult as it was needed to prolong the HPLC gradient (of MeCN, phase B, in 0.1 M TEAB aqueous solution, phase A) from 1 h to 2 h to obtain pure ON9^A^* and ON10^A^*. Isolated yields (Scheme 2 and Table S2) in some cases were good: 45–85% for ONs containing one modification (45% for ON2^AiPr^, 85% for ON4^APr^, and 76% for ON6^APh^), three pentylC modifications (ON5^Apr^, 56%) and combination of pentylC with phenylethylU modifications (ON8^A^*, 76%). On the other hand, only 14% isolated yield was obtained when three phenylethylU modifications were incorporated in a row (ON7^APh^). Lower yields (7–27%) were also obtained for other ONs containing multiple modifications (22% for ON3^AiPr^, 8% for ON9^A^*, and 7% for ON10^A^*) or containing single modification of indolylethylA (ON1^AIn^, 27%).

The purity of synthesized ONs* was determined by UHPLC-MS and in most cases was above 96% except ON6^APh^ and ON10^A^* with purities 91% and 88%, respectively (see details in SI, Table S2). Pure modified ONs were successfully isolated in sufficient amounts for following studies (avg. 2 mm in 200 µl). It should be mentioned, that during the course of our work, a preprint^40^ has been released reporting the use of related unprotected indol-linked phosphoramidites in the oligonucleotide synthesis, but this approach did not work in our hands.

The hydrophobic modifications most likely decrease the solubility of the resulting ONs in water, since the individual modifiers have lower solubilities in water (benzene 1.8 g L^−1^, indole 3.6 g L^−1^, propane 0.07 g L^−1^ at 25 °C) then the nucleosides (uridine 50 g L^−1^, adenosine 25 g L^−1^, cytidine 36 g L^−1^, guanosine 10 g L^−1^).^41^ However, we did not observe any precipitation in water or in buffer solutions, therefore, we imply all of the studied ONs are soluble in water and commonly used buffers.

The yields of the modified ONs (Scheme 2 and Table S2 in SI) synthesized from the hydrophobically modified phosphoramidites 2a–2d are lower compared to usual yields for non-modified ONs, especially for the hypermodified sequences, but still mostly somewhat better than for the previously reported alkynyl-linked phosphoramidites (1a–1b).

## Hybridization of modified oligonucleotides

**Fig. 2:**
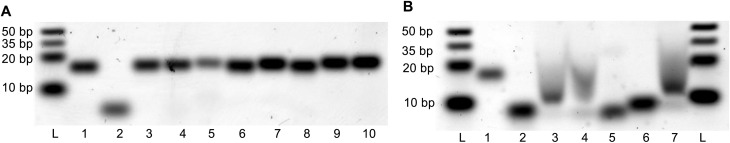
(A): Agarose gel analysis of (1) non-modified DNA1, (2) non-modified ON1 (3–10) modified DNA1^AIn^–DNA8^A^*; (L): DNA ladder; (B): agarose gel analysis of (1) non-modified DNA9, (2) non-modified ON9, (3) fully modified ON9^A^*, (4) fully modified ON10^A^*, (5) fully modified ON9^A^* with non-modified cON9 (DNA9^A^*), (6) fully modified ON10^A^* with non-modified ON9 (DNA10^A^*), (7) fully modified ON9^A^* with fully modified ON10^A^* (DNA11^A^*); (L): DNA ladder.

All partially modified ONs (ON1^AIn^–ON8^A^*) were annealed with non-modified complementary strands (cON1–cON8) from commercial supplier (to form DNA1^AIn^–DNA8^A^*, Table 1) and compared by gel electrophoresis with matching non-modified DNA (see sequences in SI, Table S3). As expected, the size of unmodified and partially modified DNA was very similar, as shown by mobility on the agarose gel (Fig. 2).

**Table 1:** List of synthesized modified oligonucleotides (ON^A^*) with purcharsed non-modified complementary strands (cON) forming modified duplexes DNA^A^* (15 bp) with modifications attached to the nucleobase *via* ethyl linker (DNA^A^*; A = alkyl/ethyl linker, * = modification)

Code		DNA^A^*
DNA1^AIn^	ON1^AIn^	5′-ATCTCAGA^AIn^GAACTGC-3′
	cON1	3′-TAGAGTC T CTTGACG-5′
DNA2^AiPr^	ON2^AiPr^	5′-ATCTCAGG^AiPr^AAGCTGC-3′
	cON2	3′-TAGAGTC C TTCGACG-5′
DNA3^AiPr^	ON3^AiPr^	5′-ATGTCAG^AiPr^G^AiPr^G^AiPr^AGCTGC-3′
	cON3	3′-TACAGT C C C TCGACG-5′
DNA4^APr^	ON4^APr^	5′-GCTCCGTC^APr^GATTGAA-3′
	cON4	3′-CGAGGCA G CTAACTT-5′
DNA5^APr^	ON5^APr^	5′-GCTCCC^APr^C^APr^C^APr^GATTGAA-3′
	cON5	3′-CGAGG G G G CTAACTT-5′
DNA6^APh^	ON6^APh^	5′-GCTCCGTU^APh^GATTGAA-3′
	cON6	3′-CGAGGCA A CTAACTT-5′
DNA7^APh^	ON7^APh^	5′-GCTCCGU^APh^U^APh^U^APh^ATTGAA-3′
	cON7	3′-CGAGGC A A A TAACTT-5′
DNA8^A^*	ON8^A^*	5′-GCTCGC^APr^U^APh^C^APr^U^APh^ATTGAA-3′
	cON8	3′-CGAGC G A G A TAACTT-5′
DNA9^A^*	ON9^A^*	5′-G^AiPr^U^APh^A^AIn^G^AiPr^A^AIn^U^APh^G^AiPr^C^APr^A^AIn^C^APr^U^APh^C^APr^G^AiPr^U^APh^C^APr^-3′
	cON9	3′-C A T C T A C G T G A G C A G-5′
DNA10^A^*	ON10^A^*	5′-G^AiPr^A^AIn^C^APr^G^AiPr^A^AIn^G^AiPr^U^APh^G^AiPr^C^APr^A^AIn^U^APh^C^APr^U^APh^A^AIn^C^APr^-3′
	ON9	3′-C T G C T C A C G T A G A T G-5′
DNA11^A^*	ON9^A^*	5′-G^AiPr^U^APh^A^AIn^G^AiPr^A^AIn^U^APh^G^AiPr^C^APr^A^AIn^C^APr^U^APh^C^APr^G^AiPr^U^APh^C^APr^-3′
	ON10^A^*	3′-C^APr^A^AIn^U^APh^C^APr^U^APh^A^AIn^C^APr^G^AiPr^U^APh^G^AiPr^A^AIn^G^AiPr^C^APr^A^AIn^G^AiPr^-5′

Nonetheless, in case of the hypermodified strands combined with non-modified complementary strand (forming DNA9^A^*, DNA10^A^*) and in case of both hypermodified complementary strands (forming DNA11^A^*), we were unable to confirm the formation of double-stranded DNA on the gel (Fig. 2), even though we tried different buffer compositions and various ionic strengths to promote the annealing (see details and gels in SI).

### Circular dichroism spectroscopy

**Fig. 3:**
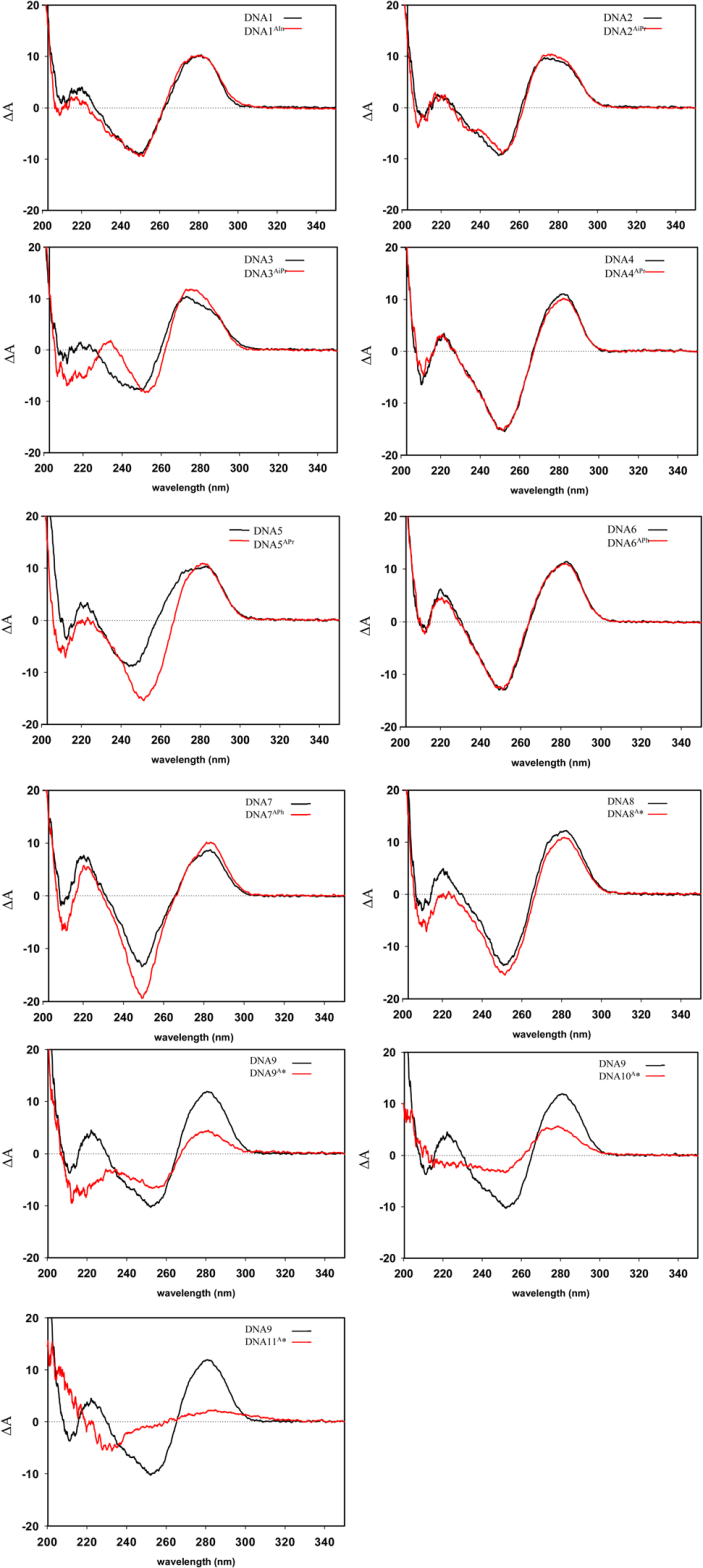
CD Spectra of modified DNA1^AIn^–DNA11^A^* (red curves) compared with their non-modified equivalent DNA1–DNA9 (black curves).

The structure of synthetic DNA duplexes (DNA1^AIn^–DNA8^A^*) was studied by circular dichroism (CD) spectroscopy. CD spectra for non-modified DNAs had characteristic conservative pattern for B-form DNA thus showing positive band at ∼280 nm and a negative band at around ∼255 nm (Fig. 3). Eventual difference in spectral shape and/or their intensity is due to difference in primary sequence of nucleotides. CD spectra of the partially modified DNA had rather similar pattern indicating that the modified DNA duplexes probably adopt B-form conformation. The observed variations in spectral patterns and intensities, relative to the corresponding unmodified DNA, can be attributed to the introduced oligonucleotide modifications (Fig. 3). While a single modification exerts minimal influence on the CD spectrum, the incorporation of three or more modifications results in pronounced spectral alterations.

**Fig. 4:**
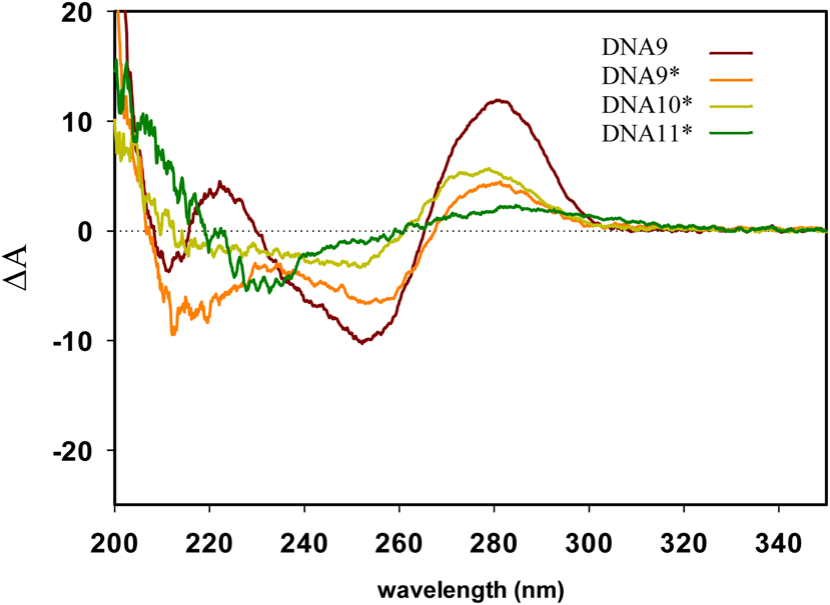
Comparison of CD spectra at 5 °C of non-modified DNA9 and corresponding modified sequences of DNA9^A^*, DNA10^A^* and hyper-modified DNA11^A^*.

Though the pattern of CD spectrum obtained for DNA9^A^* (Fig. 4), with one strand modified and the complementary non-modified, seems rather similar as those for non-modified DNA9, however, its low intensity reflects mostly the presence of unannealed oligonucleotides in agreement with agarose gel analysis (Fig. 2, lane 5).

For DNA10^A^*, similarly as DNA9^A^* with one strand modified and the complementary strand non-modified, or for DNA11^A^*, the hyper-modified duplex with two fully modified strands, the duplex formation is not detected, neither by the agarose gel analysis, nor by the CD spectroscopy (Fig. 4). Presumably, the multiple adjacent highly hydrophobic moieties tend to form aggregates or micelle-like structures that prevent the hypermodified ONs from hybridization into DNA duplex. This is in contrast to previously reported hypermodified rigid alkynyl-linked ONs that were able to form the duplexes. However, amphiphilic DNA is generally known to aggregate, as is in the case of cholesterol-modified DNA,^42–44^ where aggregation causes a challenge while studying membrane proteins.^45^ Example of base-modified ONs with 5-(dodec-1-ynyl)uracil exhibited micelle formation, whose size, and stability depended strongly on the number of hydrophobic units, rather than their position in the sequence.^46^

**Fig. 5:**
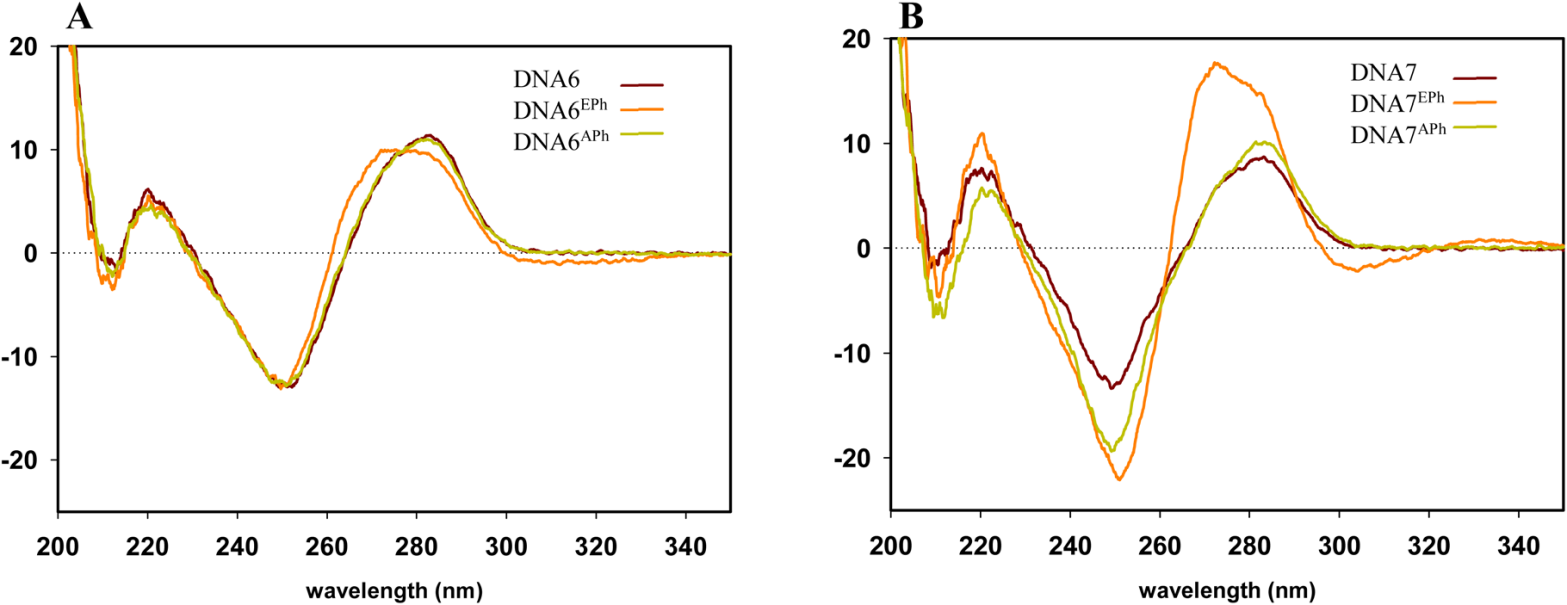
(A): CD spectra of modified DNA6^EPh^ and DNA6^APh^ of the same sequence compared with the corresponding non-modified sequence (DNA6). (B): CD spectra of modified DNA7^EPh^ and DNA7^APh^ compared with the corresponding non-modified sequence (DNA7). CD spectra were measured at 5 °C.

To further examine the effect of the linker on the duplex behavior, CD spectra of DNA6^EPh^ and DNA7^EPh^ were additionally measured (Fig. 5). One modification in the middle for both types of modification linkers cause only minor changes in the CD spectra compared to non-modified DNA duplex (DNA6^E/APh^ in comparison with non-modified DNA6, Fig. 5A). The overall shape of obtained CD spectra confirms that DNA6^E/APh^ form B-type DNA conformation.

Modified DNA7^E/APh^ still retain B-form DNA, according to measured CD spectra (Fig. 5B). Introducing three modifications cause spectral changes especially in case of ethynyl-phenyl modifier. Observed effects are influenced by the presence of additional chromophores in conjugation with the nucleobase and their eventual exciton coupling. Specifically, the spectral changes including the emergence of the negative spectral band at ∼305 nm accompanied by the blue shift of positive band from ∼285 nm to ∼275 nm together with increasing its intensity. Ethyl-linked phenyl modifications only cause increase in negative spectral band at ∼250 nm compared to non-modified DNA7, however, overall change in the spectra are minimal indicating structural likeness with corresponding non-modified duplex.

### Dynamic light scattering measurements

To qualitatively assess the aggregation state of the presented hyper-modified ONs, dynamic light scattering (DLS) measurements were performed, and the derived count rate (DCR) was compared. The DCR normalizes the measured scattered light intensity by accounting for instrument attenuation, allowing for a direct comparison of the overall scattering signal between samples.

Hyper-modified ON9^A^* and ON10^A^* exhibited a higher DCR (2555 ± 107.1 kcps and 6139 ± 278.7 kcps, respectively) compared to the non-modified ON9 (297 ± 40.2 kcps) and ON10 (568 ± 51.8 kcps). Given that the ONs were measured under the same conditions (0.5 mg per ml, in H_2_O, 25 °C), the increase in light scattering intensity is characteristic for the presence of larger scattering species.

While the poor quality of the measured autocorrelation functions precluded the derivation of reliable quantitative size metrics (*e.g.*, *Z*-average or Polydispersity Index, Table S7 in SI), the clear difference in normalized scattering intensity supports the hypothesis that the hydrophobic modification in ON9^A^* and ON10^A^* drives the formation of large, unstable aggregates in solution.

### Duplex stability

Melting (*T*_m_, Table 2) and annealing (*T*_a_, see Table S4 in SI) temperatures of all modified and non-modified DNA duplexes were measured by UV-vis spectroscopy at 260 nm, and also by CD spectroscopy (see Table S5 in SI).

**Table 2:** Comparison of melting temperatures (*T*_m_ [°C]) of non-modified (DNA) and modified DNA duplexes (DNA^A/E^*, E stands for ethynyl linker, A stands for alkyl/ethyl linker) determined by UV-vis spectroscopy at 260 nm, their sequences (X stands for A/E), the absolute difference of *T*_m_ between modified DNAs and non-modified DNAs (DNA^A/E^* Δ*T*_m_,[°C]), and the difference of *T*_m_ between modified DNAs and non-modified DNAs per modification (DNA^A/E^* Δ*T*_m_/modification, DNA^A/E^* Δ*T*_m_/modification [°C])

Code	Sequence	DNA^A^* Δ*T*_m_	DNA^E^* Δ*T*_m_^b^	DNA^A^* Δ*T*_m_/ mod	DNA^E^* Δ*T*_m_/ mod^b^
DNA1^A/EIn^	5′-ATCTCAGA^XIn^GAACTGC-3′	−5.5	−2.2	−5.5	−2.2
	3′-TAGAGTC T CTTGACG-5′				
DNA2^A/EiPr^	5′-ATCTCAGG^XiPr^AAGCTGC-3′	−4.4	−1.2	−4.4	−1.2
	3′-TAGAGTC C TTCGACG-5′				
DNA3^A/EiPr^	5′-ATGTCAG^XiPr^G^XiPr^G^XiPr^AGCTGC-3′	−8.1	−1.9	−2.7	−0.6
	3′-TACAGT C C C TCGACG-5′				
DNA4^A/EPr^	5′-GCTCCGTC^XPr^GATTGAA-3′	−5.3	+0.2	−5.3	+0.2
	3′-CGAGGCA G CTAACTT-5′				
DNA5^A/EPr^	5′-GCTCCC^XPr^C^XPr^C^XPr^GATTGAA-3′	−4.6	+7.2	−1.5	+2.4
	3′-CGAGG G G G CTAACTT-5′				
DNA6^A/EPh^	5′-GCTCCGTU^XPh^GATTGAA-3′	−7.5	−1.1	−7.5	−1.1
	3′-CGAGGCA A CTAACTT-5′				
DNA7^A/EPh^	5′-GCTCCGU^XPh^U^XPh^U^XPh^ATTGAA-3′	−11.2	−1.0	−3.7	−0.3
	3′-CGAGGC A A A TAACTT-5′				
DNA8^A/E^*	5′-GCTCGC^XPr^U^XPh^C^XPr^U^XPh^ATTGAA-3′	−10.8	+6.3	−2.7	+1.6
	3′-CGAGC G A G A TAACTT-5′				
DNA9^A/E^*	5′-G^XiPr^U^XPh^A^XIn^G^XiPr^A^XIn^U^XPh^G^XiPr^	ND^a^	+7.1	ND^a^	+0.5
	C^XPr^A^XIn^C^XPr^U^XPh^C^XPr^G^XiPr^U^XPh^C^XPr^-3′				
	3′-CATCTACGTGAGCAG-5′				
DNA10^A/E^*	5′-G^XiPr^A^XIn^C^XPr^G^XiPr^A^XIn^G^XiPr^U^XPh^	ND^a^	+7.4	ND^a^	+0.5
	G^XiPr^C^XPr^A^XIn^U^XPh^C^XPr^U^XPh^A^XIn^C^XPr^-3′				
	3′-CTGCTCACGTAGATG-5′				
DNA11^A/E^*	5′-G^XiPr^U^XPh^A^XIn^G^XiPr^A^XIn^U^XPh^G^XiPr^	ND^a^	+6.6	ND^a^	+0.2
	C^XPr^A^XIn^C^XPr^U^XPh^C^XPr^G^XiPr^U^XPh^C^XPr^-3′				
	3′-C^XPr^A^XIn^U^XPh^C^XPr^U^XPh^A^XIn^C^XPr^				
	G^XiPr^U^XPh^G^XiPr^A^XIn^G^XiPr^C^XPr^A^XIn^G^XiPr^-5′				

a ND: not determined.

b Taken from ref. 30.

The *T*_m_ data from UV-vis spectroscopy show that the stability of the modified DNA duplexes are significantly lower compared to their unmodified variants. The most prominent decrease of stability is visible for DNA6^APh^ bearing single ethylphenyl modification (−7.5 °C), followed by singly modified DNA1^AIn^ (−5.5 °C), DNA4^APr^ (−5.3 °C) and DNA2^AiPr^ (−4.4 °C). These results suggest one modification has higher destabilization potency than more incorporated modifications. In the case of hypermodified constructs DNA9^A^*, DNA10^A^* and DNA11^A^* the melting temperatures could not be reliably measured as they did not form duplexes.

In comparison with previously published series with ethynyl linker (DNA^E^*)^36^ we can see that DNA duplexes formed from ONs containing modifications linked by ethynyl linker have generally higher *T*_m_ than in the ethyl-linked series (Table 2). The biggest difference is visible with singly phenyl-substituted U-modified DNA6^A/EPh^ where with ethynyl linker the *T*_m_ is lower by −1.1 °C in comparison with corresponding non-modified DNA6 whereas by −7.5 °C when ethyl linker is present. In some cases, the *T*_m_ of ethynyl-linked modified DNAs is higher compared to the corresponding non-modified DNAs. Namely, those bearing modified C or U (DNA5^EPr^, DNA6^EPr^, DNA8^E^*) and hyper-modified DNAs with either one or both strands fully modified (DNA9^E^*, DNA10^E^*, DNA11^E^*) are showing higher stability than the non-modified version. We suggested that the stabilizing effect of multiple ethynyl-linked modifications might be caused by stabilizing π–π interactions of the ethynyl-linked modifications next to each other, whereas the flexible ethyl linkers cannot provide such stabilization and rather destabilize the duplex due to adding more energy to the system.

The previous study of nuclease resistance of hydrophobically modified ONs with the same modifications differing in ethynyl linkers^32^ showed no significant increase or decrease in enzymatic stability in comparison with non-modified ONs. Another study with 2′-*O*-alkylcarbamoylethyl modifications revealed similar findings,^47^ that length of the alkyl chain (C1–C5) did not significantly alter the nuclease resistance until longer chain was introduced (C8). We assume that the enzymatic stability of our ONs with ethyl-linked hydrophobic modifications would be showing similar trend.

## Conclusions

We have synthesized a set of alkyl and arylethyl-linked pyrimidine and 7-deazapurine 2′-deoxyribonucleoside phoshoramidites and used them in solid-supported synthesis of modified and hypermodified oligonucleotides bearing flexible hydrophobic groups. The yields of the ONs bearing the flexible alkyl groups were mostly better than the previously reported ethynyl-linked ONs. The annealing of the partially modified ONs with non-modified complementary strands successfully formed DNA duplexes, whereas the hypermodified strands did not form double-stranded B-DNA. CD spectra for all partially modified DNA duplexes showed B-form like pattern. Apparently, the hypermodified ONs bearing multiple hydrophobic modifications in a row form aggregates in aqueous solution, as supported by DLS, that prevent hybridization and forming of dsDNA. However, partially modified ONs did not suffer from this problem, hence the potential usage of such hydrophobic modifications for partial modification of ONs and in selections remains to be feasible. The melting temperatures studies showed, that the overall stability of the modified duplexes with ethyl linker is lower than the stability of non-modified corresponding DNAs and of the ethynyl-linked modified DNA duplexes. The reported phosphoramidites will be useful reagents in solid-phase automated synthesis of partially modified ONs in aptamer engineering^6^ and other functional nucleic acids, as well as potential oligonucleotide therapeutics. For potential applications as therapeutics further studies on cellular uptake, nuclease resistance, and immunogenicity would need to be performed.

## Conflicts of interest

The authors declare no competing financial interest.

## Supplementary Material

RA-015-D5RA06147D-s001

## Data Availability

The data supporting this article have been included as part of the supplementary information (SI). Supplementary information: synthetic protocols and characterization of new compounds, additional tables, chromatograms, melting curves, additional CD spectra and copies of NMR and MS spectra. See DOI: https://doi.org/10.1039/d5ra06147d.

## References

[cit1] McKenzie L. K., El-Khoury R., Thorpe J. D., Damha M. J., Hollenstein M. (2021). Chem. Soc. Rev..

[cit2] Röthlisberger P., Hollenstein M. (2018). Adv. Drug Delivery Rev..

[cit3] Vaught J. D., Bock C., Carter J., Fitzwater T., Otis M., Schneider D., Rolando J., Waugh S., Wilcox S. K., Eaton B. E. (2010). J. Am. Chem. Soc..

[cit4] Rohloff J. C., Gelinas A. D., Jarvis T. C., Ochsner U. A., Schneider D. J., Gold L. (2014). Mol. Ther. Nucleic Acids.

[cit5] Cheung Y.-W., Röthlisberger P., Mechaly A. E., Weber P., Levi-Acobas F., Lo Y., Wong A. W. C., Kinghorn A. B., Haouz A., Savage G. P., Hollenstein M., Tanner J. A. (2020). Proc. Natl. Acad. Sci. U.S.A.

[cit6] Mulholland C., Jestrábová I., Sett A., Ondruš M., Sýkorová V., Manzanares C. L., Šimončík O., Muller P., Hocek M. (2023). Commun. Chem..

[cit7] Gold L., Ayers D., Bertino J., Bock C., Bock A., Brody E. N., Carter J., Dalby A. B., Eaton B. E., Fitzwater T., Flather D., Forbes A., Foreman T., Fowler C., Gawande B., Goss M., Gunn M., Gupta S., Halladay D., Heil J., Heilig J., Hicke B., Husar G., Janjic J., Jarvis T., Jennings S., Katilius E., Keeney T. R., Kim N., Koch T. H., Kraemer S., Kroiss L., Le N., Levine D., Lindsey W., Lollo B., Mayfield W., Mehan M., Mehler R., Nelson S. K., Nelson M., Nieuwlandt D., Nikrad M., Ochsner U., Ostroff R. M., Otis M., Parker T., Pietrasiewicz S., Resnicow D. I., Rohloff J., Sanders G., Sattin S., Schneider D., Singer B., Stanton M., Sterkel A., Stewart A., Stratford S., Vaught J. D., Vrkljan M., Walker J. J., Watrobka M., Waugh S., Weiss A., Wilcox S. K., Wolfson A., Wolk S. K., Zhang C., Zichi D. (2010). PLoS One.

[cit8] Davies D. R., Gelinas A. D., Zhang C., Rohloff J. C., Carter J. D., O'Connell D., Waugh S. M., Wolk S. K., Mayfield W. S., Burgin A. B., Edwards T. E., Stewart L. J., Gold L., Janjic N., Jarvis T. C. (2012). Proc. Natl. Acad. Sci. U.S.A..

[cit9] Ondruš M., Franco-Urquijo P. A., Lerga T. M., Mužíková Cechová L., Bermudo Redondo M. C., O´Sullivan C. K., Hocek M. (2025). ChemBioChem.

[cit10] Flamme M., Niogret G., McKenzie L., Akhtar U., Levi-Acobas F., Gandioso A., Clarke E., Bizat P. N., Connor M., Hamon M. A., Gasser G., Hollenstein M. (2025). J. Am. Chem. Soc..

[cit11] Gawande B. N., Rohloff J. C., Carter J. D., von Carlowitz I., Zhang C., Schneider D. J., Janjic N. (2017). Proc. Natl. Acad. Sci. U. S. A.

[cit12] Liu K., Robinson N., Tekoglu E., Lozada J., Yu I. P. L., Tai R. A., Ozturan D., Dikbas U. M., Lack N. A., Cox M. E., Mannas M. P., Cohen P., Goldenberg S. L., Perrin D. M. (2025). J. Am. Chem. Soc..

[cit13] Wagner R. W. (1994). Nature.

[cit14] Wagner R. W., Matteucci M. D., Lewis J. G., Gutierrez A. J., Moulds C., Froehler B. C. (1993). Science.

[cit15] Wagner R. W., Matteucci M. D., Grant D., Huang T., Froehler B. C. (1996). Nat. Biotechnol..

[cit16] Barnes T. W., Turner D. H. (2001). J. Am. Chem. Soc..

[cit17] Gyi J. I., Gao D., Conn G. L., Trent J. O., Brown T., Lane A. N. (2003). Nucleic Acids Res..

[cit18] Kottysch T., Ahlborn C., Brotzel F., Richert C. (2004). Chem. Eur J..

[cit19] Buhr C. A., Wagner R. W., Grant D., Froehler B. C. (1996). Nucleic Acids Res..

[cit20] Seela F., Zulauf M. (1998). Chem. Eur J..

[cit21] Seela F., Shaikh K. I. (2005). Tetrahedron.

[cit22] Mikami A., Mori S., Osawa T., Obika S. (2023). Chem. Eur J..

[cit23] Yoshida T., Morihiro K., Naito Y., Mikami A., Kasahara Y., Inoue T., Obika S. (2022). Nucleic Acids Res..

[cit24] Renders M., Miller E., Lam C. H., Perrin D. M. (2017). Org. Biomol. Chem..

[cit25] Vaníková Z., Janoušková M., Kambová M., Krásný L., Hocek M. (2019). Chem. Sci..

[cit26] Gracias F., Pohl R., Sýkorová V., Hocek M. (2024). Commun. Chem..

[cit27] Jäger S., Famulok M. (2004). Angew. Chem., Int. Ed..

[cit28] Jäger S., Rasched G., Kornreich-Leshem H., Engeser M., Thum O., Famulok M. (2005). J. Am. Chem. Soc..

[cit29] Ondruš M., Sýkorová V., Bednárová L., Pohl R., Hocek M. (2020). Nucleic Acids Res..

[cit30] Ondruš M., Sýkorová V., Hocek M. (2022). Chem. Commun..

[cit31] Kuprikova N., Ondruš M., Bednárová L., Riopedre-Fernández M., Poštová Slavětínská L., Sýkorová V., Hocek M. (2023). Nucleic Acids Res..

[cit32] Kuprikova N., Ondruš M., Bednárová L., Hocek M. (2025). Nucleic Acids Res..

[cit33] Krömer M., Poštová Slavětínská L., Hocek M. (2024). Chem. Eur J..

[cit34] Kaiser L., Ondruš M., Poštová Slavětínská L., Raindlová V., Hocek M. (2025). Chem. Eur J..

[cit35] Beaucage S. L., Caruthers M. H. (1981). Tetrahedron Lett..

[cit36] Jestrábová I., Poštová Slavětínská L., Hocek M. (2023). ACS Omega.

[cit37] Chakrapani A., Vaňková Hausnerová V., Ruiz-Larrabeiti O., Pohl R., Krásný L., Hocek M. (2020). Org. Lett..

[cit38] Seela F., Budow S., Shaikh K. I., Jawalekar A. M. (2005). Org. Biomol. Chem..

[cit39] Sánchez-Cantalejo F., Priest J. D., Davies P. W. (2018). Chem. Eur J..

[cit40] Lingala S., Fisiuk A., Stephen M., Mohanrao R., Klingsberg J., Vecchioni S., Volvovitz E. S., Rozhkov S., Mallikaratchy P. (2025). ACS Omega.

[cit41] PubChem, NCBI, accessed [28/09/2025], https://pubchem.ncbi.nlm.nih.gov/

[cit42] Krishnan S., Ziegler D., Arnaut V., Martin T. G., Kapsner K., Henneberg K., Bausch A. R., Dietz H., Simmel F. C. (2016). Nat. Commun..

[cit43] Johnson-Buck A., Jiang S., Yan H., Walter N. G. (2014). ACS Nano.

[cit44] Kocabey S., Kempter S., List J., Xing Y., Bae W., Schiffels D., Shih W. M., Simmel F. C., Liedl T. (2015). ACS Nano.

[cit45] Gubu A., Zhang X., Lu A., Zhang B., Ma Y., Zhang G. (2023). Mol. Ther. Nucleic Acids.

[cit46] Anaya M., Kwak M., Musser A. J., Mühlen K., Herrmann A., Müllen K. (2010). Chem.

[cit47] Kishimura T., Tomori T., Masaki Y., Seio K. (2021). Bioorg. Med. Chem. Lett..

