# Supplementary material for: Hydrophobic alkyl-linked base-modified pyrimidine and 7-deazapurine 2′-deoxyribonucleoside phosphoramidites: synthesis and application in solid-phase synthesis of modified and hypermodified oligonucleotides
Source: RSC Adv. 2025 Dec 8;15(56):48556–65. doi: 10.1039/d5ra06147d (PMC12683653; doi:10.1039/d5ra06147d)
Supplement: RA-015-D5RA06147D-s001 [file RA-015-D5RA06147D-s001.pdf]

Supplementary Information (SI) for RSC Advances.
This journal is © The Royal Society of Chemistry 2025

ELECTRONIC SUPPORTING INFORMATION

Hydrophobic Alkyl-Linked Base-Modified Pyrimidine and 7-Deazapurine 2′-Deoxyribonucleoside Phosphoramidites: Synthesis and Application in Solid-Phase Synthesis of Modified and Hypermodified Oligonucleotides

Ivana Jestřábová,[a,b] Lucie Bednárová,[a] Lenka Poštová Slavětínská,[a] Michal Hocek*[a,b]

*a) Institute of Organic Chemistry and Biochemistry, Czech Academy of Sciences, Flemingovo nam. 2, CZ-16000 Prague 6, Czech Republic; *hocek@uochb.cas.cz*

b) Department of Organic Chemistry, Faculty of Science, Charles University, Hlavova 8, CZ-12843 Prague 2, Czech Republic

Contents

General Remarks 3

1 Synthesis of Phosphoramidites 5

1.1 Synthesis of Published Compounds 5
1.2 General Method A: Dimethyltritylation of 5′-OH 5
1.3 General Method B: Reduction of ethynyl to ethyl linker 5
1.4 General Method C: Synthesis of 3′-Phosphoramidites 5
1.5 Synthesis of Modified 2′-Deoxyuridine 6
1.6 Synthesis of Modified 2′-Deoxycytidine 9
1.7 Synthesis of Modified 2′-Deoxyadenosine 13
1.8 Synthesis of Modified 2′-Deoxyguanosine 16

2 Synthesis of Modified Oligonucleotides 20

2.1 Solid-phase Synthesis 20
2.2 Purification and Characterization 20
2.3 Hybridization Experiments 22
2.4 Stability Analysis 22
2.5 Dynamic Light Scattering 25

3 Copies of Chromatograms, Spectra and Graphs 26

3.1 HPLC Chromatograms of Synthesized Oligonucleotides 26
3.2 Absorbance Chromatograms of Purified Oligonucleotides 32
3.3 ESI Spectra of Oligonucletotides 37
3.4 Melting and Annealing Curves Measured by UV-vis Absorption 47
3.5 Melting Temperatures Measured by Circular Dichroism Spectroscopy 50
3.6 Circular Dichroism Spectroscopy Spectra 53
3.7 Copies of NMR Spectra 56

4 Reference List 78

General Remarks

Chemicals were purchased from usual commercial suppliers, such as Fluorochem, Sigma Aldrich and Enamine. Solvents were removed *in vacuo* with the bath temperature between 40–60 °C. Reactions were monitored by silica gel thin-layer chromatography (TLC) in Merck silica gel 60 F_{254} plates with UV light detection (254 and 365 nm) combined with visualisation by the solution of 4-anisaldehyde in ethanol with sulphuric acid (10%). The masses of individual spots on TLC plate were measured by Advion Expression Compact Mass Spectrometer connected with Plate Express TLC Plate Reader (TLC-MS) using electrospray ionization (ESI). Isolation of nucleosides was carried out on CombiFlash Rf+ (Teledyne Isco) with Silicagel 40–63 μm stationary phase from VWR International or C18-HP 30 μm from Interchim.

Purity of all compounds was analyzed by NMR and HR-MS spectra. ^{1}H and ^{13}C NMR spectra were measured on Bruker Avance III HD 500 MHz (^{1}H at 500.0 MHz, ^{13}C at 125.7 MHz and ^{31}P at 202.4 MHz) and JEOL ECZR 500 MHz (^{1}H at 500.2 MHz, ^{13}C at 125.8 MHz and ^{31}P at 202.5 MHz) in DMSO-*d6*, D_2O or CD_3CN referenced to the residual solvent signal. Chemical shifts are given in ppm (δ-scale), coupling constants (J) in Hz. Determination of all NMR signals was accomplished using a combination of H,H-COSY, H,C-HSQC and H,C-HMBC experiments. Low and high resolution mass spectra of small molecules were measured on LTQ Orbitrap XL spectrometer (ESI ionization, Thermo Fisher Scientific). The matrix (1 μL) was applied to the target (ground steel) and dried down at room temperature. Mass of the small molecules were acquired by the MS service at IOCB. MestreNova (version 14) from Mestrelab Research and MSView from Morningstar was used for data analysis.

Reagents and solvents for the solid-phase synthesis of oligonucleotides were purchased from Sigma-Aldrich, Link Technologies, and Thermo Fisher-Scientifics. Modified oligonucleotides were synthesized through standard phosphoramidite chemistry with an automated DNA solid-phse synthesizer (Mermade 8, BioAutomation Corporation) either on standard or universal solid-phase columns (500 Å). Purification of the prepared oligonucleotides was performed using semi-preparative HPLC (Waters modular HPLC system) on a column packed with C18 reverse phase (5% to 100% of MeCN in aqueous 0.1M TEAB solution, Column XBridge Prep C18 5 μm, OBD from Waters). The analysis of ONs was performed by UHPLC-MS-ESI (Agilent 1290 Infinity II Bio LC System with DAD detector and mass spectrometer MSD XT). The analysis was

carried out according to standard procedures using mobile phases A (12.2 mM TEA (triethylamine), 300 mM HFIP (hexafluoro-2-propanol) in H_2O) and B (12.2 mM Et_3N, 300 mM HFIP in 100% MeOH) by 24 min gradient from 5% B to 100% B in A using BioZen 1.7 μm oligo column 2.1×50 mm from Phenomenex. The absorbance chromatograms were evaluated in MestreNova and the LC-ESI-MS were deconvoluted using free software UniDec.[1] Approximate concentrations of ONs solutions were determined using the Nanodrop 1000 (Thermo Fischer Scientific). The exact concentrations and yields were determined based on the phosphorus content measured by the elemental analysis of pure ONs. The non-modified oligonucleotides were ordered from Eurofins.

The circular dichroism (CD) measurements were performed on a Jasco-1500 spectropolarimeter equipped with Peltier thermostated holder PTC-517 (JASCO Inc. Easton, MD, USA). Oligonucleotides were dissolved in annealing buffer (10 mM Tris, 50 mM NaCl, 1 mM EDTA, pH 7.5 - 8.0) all of them in concentration 25μM. The spectra were measured in temperature range 5–95°C with temperature increment 5°C in spectral range from 200 nm to 400 nm in 2 mm rectangular quartz cell. Experimental setup was as following: standard instrument sensitivity, 1 nm bandwidth, a scanning speed of 10 nm/min, a response time of 8 s and one accumulation. The temperature of the sample was kept constant during each data accumulation and the same experimental setup was used for temperature increase and decrease. After baseline subtraction the final data were expressed as molar differential extinction $\Delta\varepsilon$ ($cm^{-1}mol^{-1}$). The melting temperatures were calculated using program Sigmaplot12.5 (Systat software) where sigmoid fitting was applied.

The agarose gels were visualized by GelRed (Biotium) using Amersham Typhoon (Cytiva). As a ladder served ultra-low range ladder from Invitrogen (Thermo Fischer Scientific). Melting and annealing temperatures were measured by UV-absorption measurements which were performed on Cary 100 Bio UV-vis Spectrophotometer with temperature controller (Varian) or by CD spectroscopy.

The dynamic light scattering (DLS) experiments were measured on Zetasizer Nano ZSP (Malvern) and evaluated in DTS Nano software. Solution of ONs were measured at 25°C in UPLC-grade water in 70 μl volume cuvettes.

1 Synthesis of Phosphoramidites

1.1 Synthesis of Published Compounds

Compounds **3a** and **3b** were prepared according to the reported procedures[2–4] from corresponding iodonucleosides **5a** and **6b**. 1-Acetyl-3-ethynylindole was synthesized following the published procedures[5–7] starting from 1*H*-indole. Compound **5c** was acquired by a multi-step synthesis reported in literature[2,3,8–10] from 4-chloro-7*H*-pyrrolo[2,3-d]pyrimidine. Compound **5d** was synthesized according to the published synthesis[2,11] from 4-chloro-7*H*-pyrrolo[2,3-d]pyrimidin-2-amine.

1.2 General Method A: Dimethyltritylation of 5′-OH

Precursor was dried by several co-evaporations with anhydrous pyridine (3×5 mL) and finally dissolved in anhydrous pyridine along with *N,N*-dimethylaminopyridine (DMAP, 0.1 equiv.). Solution of 4,4′-dimethoxytrityl chloride (DMTrCl, 1.2 equiv.) in anhydrous pyridine was added in 4 portions over 1 h and the reaction was stirred at room temperature overnight. The solvent was removed under reduced pressure and the crude was re-dissolved in DCM, washed with 10% aqueous solution of $NaHCO_3$, brine, and finally dried over anhydrous Na_2SO_4. Purification by high-performance liquid chromatography (HPFC; usually DCM/MeOH 0–1% with 0.5% Et_3N) afforded the desired compound.

1.3 General Method B: Reduction of ethynyl to ethyl linker

Functionalized nucleoside was dissolved in MeOH and iPrOH (3:1) and 10% Pd/C was added. Then, the atmosphere was exchanged for H_2 and the reaction was left stirring for 3 h. After confirming the completion of the reaction on TLC, the Pd/C catalyst was filtered off and solvent was evaporated to obtain pure product.

1.4 General Method C: Synthesis of 3′-Phosphoramidites

Protected nucleoside was dried by repeated co-evaporation with anhydrous pyridine (3×5 mL), followed by co-evaporation with anhydrous DCM (3×5 mL), and dried under vacuum for 30 min. Subsequently, the starting material was dissolved in anhydrous DCM in a sealed flask under argon atmosphere with molecular sieves (4 Å). Subsequently, the reaction was cooled down to 0 °C and freshly distilled *N*,*N*-diisopropylethylamine (DIPEA) was added followed by the addition of 2-cyanoethyl-*N*,*N*-

diisopropylchlorophosphoramidite. The mixture was then warmed up to room temperature and stirred until a complete conversion was observed by TLC analysis (cyclohexane/EtOAc, approx. 1.5 h). Then, the mixture was diluted with anhydrous DCM, quickly washed under an argon atmosphere with saturated aqueous solution of KI and dried over Na_2SO_4. Purification was done by reverse-phase HPFC (H_2O/MeCN 9:1 to 100% MeCN) provided final compound usually as a mixture of two diastereomers.

1.5 Synthesis of Modified 2′-Deoxyuridine

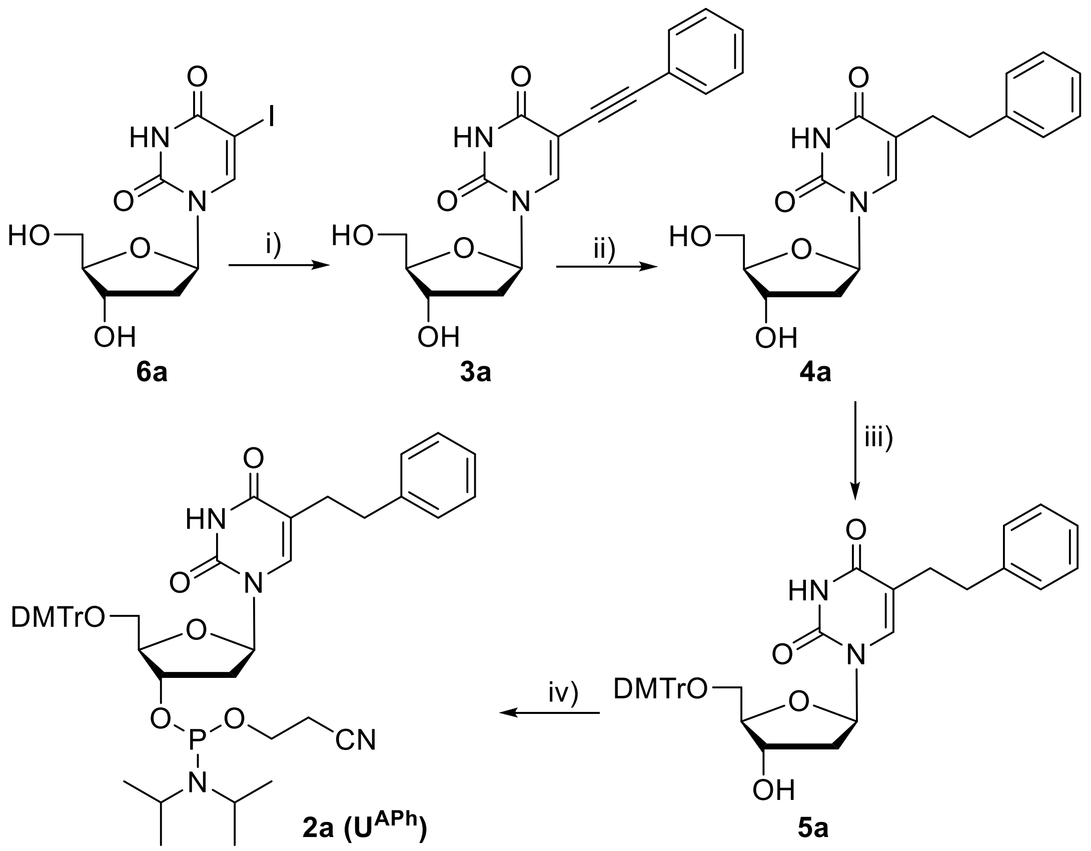

Scheme S1 Reagents and conditions: i) ethynyl benzene (10 equiv), $Pd(OAc)_2$ (0.1 equiv.), CuI (0.1 equiv.), TPPTS (0.1 equiv.), TEA (6 equiv.), MeCN/H_2O (1:1), RT, Ar, overnight; ii) H_2, 10% Pd/C (0.1 equiv.), MeOH, RT, 3 h; iii) DMTrCl (1.2 equiv.), DMAP (0.1 equiv.), pyridine (dry), RT, overnight; iv) 2-cyanoethyl-*N,N*-diisopropylchlorophosphoramidite (1.2 equiv.), DIPEA (2.5 equiv.), DCM (dry), 0 °C to RT, 1.5 h;

5'-*O*-[Bis(4-methoxyphenyl)phenylmethyl]-5-(2-phenyleth-1-yl)-2′-deoxyuridine (5a)

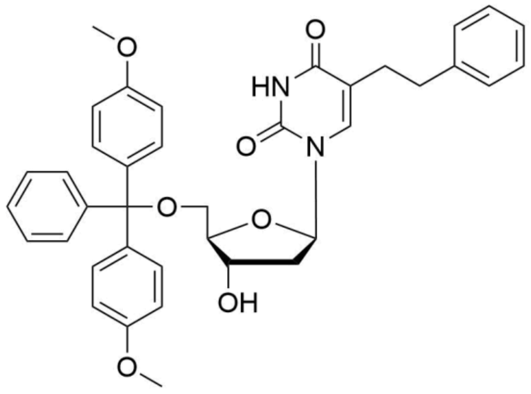

For synthesis of compound **4a** General method A was utilized. To the solution of compound **3a** (3.3 g, 9.9 mmol) in dry pyridine (45 mL), DMAP (112 mg, 1.0 mmol) and solution of DMTrCl (4.0 g, 11.9 mmol) in pyridine (15 mL) were added. The compound **4a** (4.9 g, 77%) was afforded as a greenish foam.

^{1}H NMR (500.2 MHz, DMSO-d_6): 2.08 – 2.16 (m, 3H, H-2′, CH$_a$**H$_b$**CH$_2$Ph); 2.20 (ddd, 1H, J_{gem} = 14.0, J_{vic} = 9.7, 5.9, C**H$_a$**H$_b$CH$_2$Ph); 2.46 – 2.59 (m, 2H, CH$_2$C**H$_2$**Ph, overlapped with DMSO signal); 3.13 (dd, 1H, J_{gem} = 10.4, $J_{5'b,4'}$ = 4.7, H-5′b); 3.18 (dd, 1H, J_{gem} = 10.4, $J_{5'a,4'}$ = 3.2, H-5′a); 3.68 (s, 6H, CH$_3$O-DMTr); 3.88 (dt, 1H, $J_{4',5'}$ = 4.7, 3.2, $J_{4',3'}$ = 3.2, H-4′); 4.22 (m, 1H, H-3′); 5.33 (d, 1H, $J_{OH,3'}$ = 4.5, OH-3′); 6.18 (t, 1H, $J_{1',2'}$ = 6.8, H-1′); 6.84 – 6.90 (m, 6H, H-*o*-Ph, H-*m*-C$_6$H$_4$OMe-DMTr); 7.10 (m, 1H, H-*p*-Ph); 7.14 – 7.18 (m, 2H, H-*m*-Ph); 7.20 (m, 1H, H-*p*-C$_6$H$_5$-DMTr); 7.23 – 7.31 (m, 6H, H-*o*-C$_6$H$_4$OMe-DMTr, H-*m*-C$_6$H$_5$-DMTr); 7.33 (s, 1H, H-6); 7.37 – 7.40 (m, 2H, H-*o*-C$_6$H$_5$-DMTr); 11.39 (s, 1H, NH).

^{13}C NMR (125.7 MHz, DMSO-d_6): 28.59 (**C**H$_2$CH$_2$Ph); 34.44 (CH$_2$**C**H$_2$Ph); 39.65 (CH$_2$-2′); 55.19 (CH$_3$O-DMTr); 63.98 (CH$_2$-5′); 70.69 (CH-3′); 83.99 (CH-1′); 85.64 (CH-4′); 85.93 (C-DMTr); 113.39 (C-5); 113.42 (CH-*m*-C$_6$H$_4$OMe-DMTr); 125.95 (CH-*p*-Ph); 127.01 (CH-*p*-C$_6$H$_5$-DMTr); 127.92 (CH-*o*-C$_6$H$_5$-DMTr); 128.08 (CH-*m*-C$_6$H$_5$-DMTr); 128.26, 128.31 (CH-*o,m*-Ph); 129.87, 129.91 (CH-*o*-C$_6$H$_4$OMe-DMTr); 135.58, 135.60 (C-*i*-C$_6$H$_4$OMe-DMTr); 136.12 (CH-6); 141.26 (C-*i*-Ph); 144.78 (C-*i*-C$_6$H$_5$-DMTr); 150.42 (C-2); 158.32, 158.33 (C-*p*-C$_6$H$_4$OMe-DMTr); 163.40 (C-4).

HR ESI-MS calculated m/z: 657.25712 [M+Na]$^+$, found m/z: 657.25684 [M+Na]$^+$.

5'-*O*-[Bis(4-methoxyphenyl)phenylmethyl]-5-(2-phenyleth-1-yl)-2′-deoxyuridine-3′-(2-cyanoethyl *N,N*-diisopropylphosphoramidite) (2a)

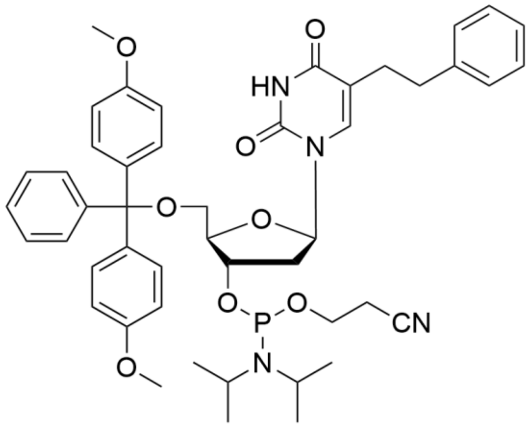

Compound **2a** was synthesized using General method C by dissolving the precursor **5a** (1.0 g, 1.6 mmol) in dry DCM (6 mL) and addition of *N*,*N*-diisopropylethylamine (DIPEA, 686 μL, 3.9 mmol) and 2-cyanoethyl-*N*,*N*-diisopropylchlorophosphoramidite (422 μL, 1.9 mmol). Purification provided the final compound **2a** (867 mg, 65%) as a 1:1 mixture of two diastereomers in a form of a white foam.

^{1}H NMR (500.0 MHz, CD_3CN): 1.03,1.14 and 1.16 (4×d, 4×3H, $J_{CH3,CH}$ = 6.8 Hz, (C**H**$_3$)$_2$CHN); 2.05 – 2.43 (m, 2×4H, H-2′, C**H**$_2$CH$_2$Ph); 2.51 and 2.64 (2×t, 2×2H, $J_{CH2,CH2}$ = 5.9 Hz, OCH$_2$C**H**$_2$CN); 2.50 - 2.64 (m, 2×2H, CH$_2$C**H**$_2$Ph); 3.16 - 3.25 (m, 2×1H, H-5′a); 3.29 - 3.38 (m, 2×1H, H-5′b); 3.72, 3.712, 3.711 and 3.710 (4×s, 4×3H, CH$_3$O-DMTr); 3.49 -3.87 (m, 2×4H, (CH$_3$)$_2$C**H**N, OC**H**$_2$CH$_2$CN), 4.04 and 4.09 (2×bq, 2×1H, $J_{4′,3′} = J_{4′,5′}$ = 3.5 Hz, H-4′); 4.50 – 4.59 (m, 2×1H, H-3′); 6.20 - 6.26 (m, 2×1H, H-1′); 6.81 – 6.86 (m, 2×4H, H-*m*-C$_6$H$_4$OMe-DMTr); 6.86 – 6.91 (m, 2×2H, H-*o*-Ph); 7.08 – 7.13 (m, 2×1H, H-*p*-Ph); 7.12 – 7.17 (m, 2×2H, H-*m*-Ph); 7.19 - 7.24 (m, 2×1H, H-*p*-C$_6$H$_5$-DMTr); 7.26 – 7.35 (m, 2×7H, H-*m*-C$_6$H$_5$-DMTr, H-*o*-C$_6$H$_4$OMe-DMTr, H-6); 7.41 – 7.47 (m, 2×2H, H-*o*-C$_6$H$_5$-DMTr); 9.02 (bs, 2×1H, NH).

^{13}C NMR (125.7 MHz, CD_3CN): 20.94 and 21.03 (2×d, $J_{C,P}$ = 7.1 Hz, OCH$_2$**C**H$_2$CN); 24.78 – 24.91 (m, (**C**H$_3$)$_2$CHN); 29.75 and 29.78 (**C**H$_2$CH$_2$Ph); 35.49 and 35.51 (CH$_2$**C**H$_2$Ph); 39.86 and 39.98 (2×d, $J_{C,P}$ = 4.5 and 3.4 Hz, CH$_2$-2′); 43.99 (d, $J_{C,P}$ = 12.3 Hz, (CH$_3$)$_2$**C**HN); 55.87 and 55.89 (CH$_3$O-DMTr); 59.42 and 59.43 (2×d, $J_{C,P}$ = 19.1 Hz, O**C**H$_2$CH$_2$CN); 64.15 and 64.28 (CH$_2$-5′); 73.93 and 74.31 (2×d, $J_{C,P}$ = 16.7 and 17.5 Hz, CH-3′); 85.21 and 85.26 (CH-1′); 85.71 and 85.99 (2×d, $J_{C,P}$ = 5.9 and 4.2 Hz, CH-4′); 87.32 and 87.35 (C-DMTr); 114.12 (CH-*m*-C$_6$H$_4$OMe-DMTr); 114.83 and 114.87 (C-5); 119.40 and 119.56 (OCH$_2$CH$_2$**C**N); 126.80 and 126.82 (CH-*p*-Ph); 127.99 and 128.01 (CH-*p*-C$_6$H$_5$-DMTr); 128.93 (CH-*o*-C$_6$H$_5$-DMTr); 129.05 and 129.11 (CH-*m*-C$_6$H$_5$-DMTr); 129.18 and 129.19 (CH-*m*-Ph); 129.29 (CH-*o*-Ph); 131.02 and 131.05 (CH-*o*-C$_6$H$_4$OMe-DMTr); 136.66, 136.70 and 136.71 (C-*i*-C$_6$H$_4$OMe-DMTr); 136.92 and

136.97 (CH-6); 142.42 (C-*i*-Ph); 145.75 and 145.77 (C-*i*-C_6H_5-DMTr); 151.21 (C-2); 159.74 (C-*p*-C_6H_4OMe-DMTr); 164.08 and 164.09 (C-4).

$^{31}P\{^1H\}$ NMR (202.4 MHz, CD_3CN): 149.34 and 149.37 (2×s, 2×1P).

HR ESI-MS calculated m/z: 835.38303 $[M+H]^+$, 857.36497 $[M+Na]^+$, found m/z: 835.38398 $[M+H]^+$, 857.36580 $[M+Na]^+$.

1.6 Synthesis of Modified 2′-Deoxycytidine

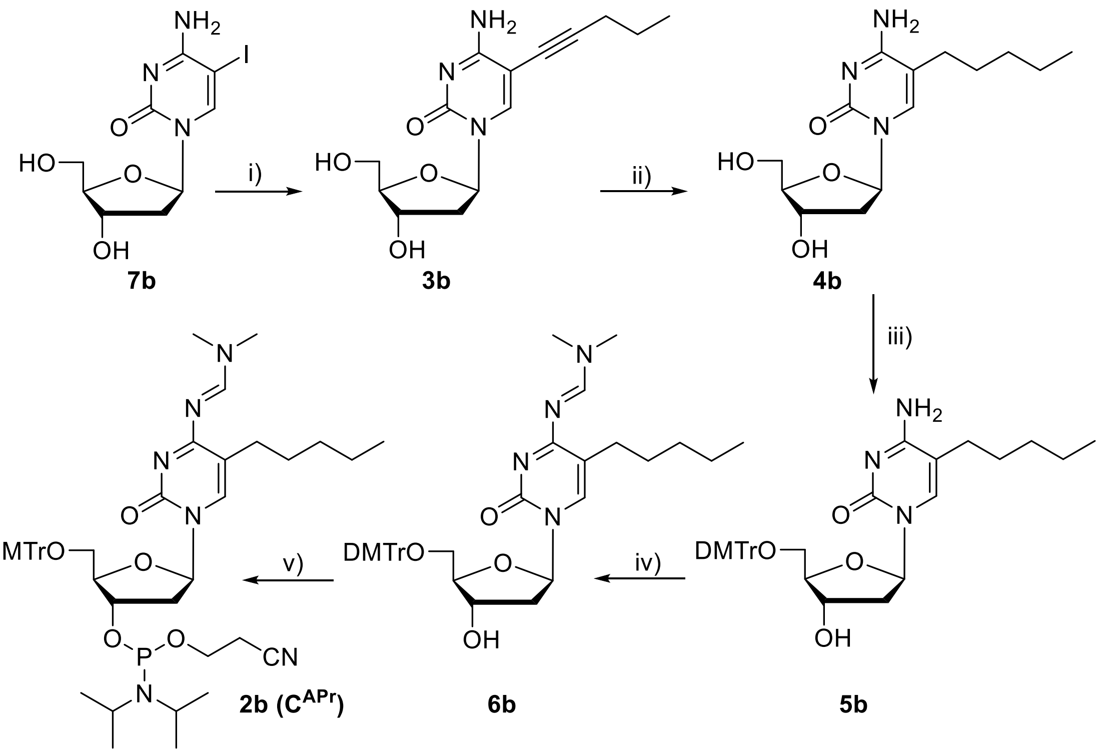

Scheme S2 Reagents and conditions: i) pent-1-yne (10 equiv.), $Pd(OAc)_2$ (0.1 equiv.), CuI (0.1 equiv.), TPPTS (0.1 equiv.), TEA (6 equiv.), MeCN/H_2O (1:1), RT, Ar, overnight; ii) H_2, 10% Pd/C (0.3 equiv.), MeOH, RT, 3 h; iii) DMTrCl (1.2 equiv.), DMAP (0.1 equiv.), pyridine (dry), RT, overnight; iv) DMF-DMA (14 equiv.), DMF (dry), 40°C, Ar, 4 h; v) 2-cyanoethyl-*N,N*-diisopropylchlorophosphoramidite (1.2 equiv.), DIPEA (2.5 equiv.), DCM (dry), 0 °C to RT, 1.5 h;

5'-*O*-[Bis(4-methoxyphenyl)phenylmethyl]-5-(pent-1-yl)-2′-deoxycytidine (5b)

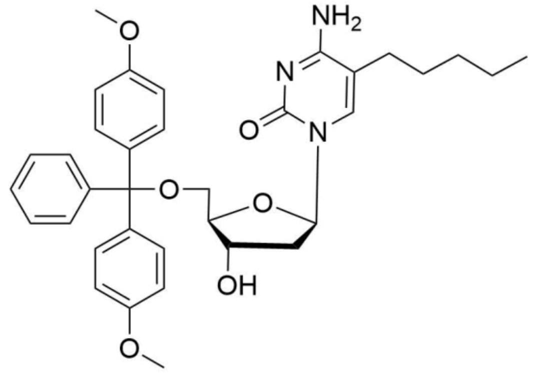

General method A was utilized to obtain compound **5b**. Upon dissolution of compound **4b** (6.0 g, 20.2 mmol) and DMAP (226 mg, 2.0 mmol) in dry pyridine (60 mL), solution of DMTrCl (8.2 g, 24.2 mmol) in 10 mL of dry pyridine was added. Final product **5b** (8.2 g, 68%) was acquired in a form of a white foam.

^{1}H NMR (500.0 MHz, DMSO-d_6): 0.71 (t, 3H, J_{vic} = 7.2, $C\mathbf{H_3}CH_2CH_2CH_2CH_2$); 0.92 – 1.23 (m, 6H, $CH_3C\mathbf{H_2}C\mathbf{H_2}C\mathbf{H_2}CH_2$); 1.81, 1.96 (2 × ddd, 2 × 1H, J_{gem} = 15.1, J_{vic} = 10.0, 5.6, $CH_3CH_2CH_2CH_2C\mathbf{H_2}$); 2.07 (ddd, 1H, J_{gem} = 13.3, $J_{2'b,1'}$ = 7.4, $J_{2'b,3'}$ = 6.4, H-2′b); 2.16 (ddd, 1H, J_{gem} = 13.3, $J_{2'a,1'}$ = 6.1, $J_{2'a,3'}$ = 3.5, H-2′a); 3.15 (dd, 1H, J_{gem} = 10.4, $J_{5'b,4'}$ = 4.2, H-5′b); 3.22 (dd, 1H, J_{gem} = 10.4, $J_{5'a,4'}$ = 3.0, H-5′a); 3.73 (s, 6H, CH_3O-DMTr); 3.88 (ddd, 1H, $J_{4',5'}$ = 4.2, 3.0, $J_{4',3'}$ = 3.5, H-4′); 4.26 (ddt, 1H, $J_{3',2'}$ = 6.4, 3.5, $J_{3',OH}$ = 4.5, $J_{3',4'}$ = 3.5, H-3′); 5.29 (d, 1H, $J_{OH,3'}$ = 4.5, OH-3′); 6.23 (dd, 1H, $J_{1',2'}$ = 7.4, 6.1, H-1′); 6.81 (bs, 1H, $NH_a\mathbf{H_b}$); 6.86 – 6.90 (m, 4H, H-*m*-C_6H_4OMe-DMTr); 7.23 (m, 1H, H-*p*-C_6H_5-DMTr); 7.24 – 7.28 (m, 5H, $N\mathbf{H_a}H_b$, H-*o*-C_6H_4OMe-DMTr); 7.28 – 7.33 (m, 2H, H-*m*-C_6H_5-DMTr); 7.37 – 7.40 (m, 3H, H-6, H-*o*-C_6H_5-DMTr).

^{13}C NMR (125.7 MHz, DMSO-d_6): 14.06 ($\mathbf{C}H_3CH_2CH_2CH_2CH_2$); 22.00 ($CH_3\mathbf{C}H_2CH_2CH_2CH_2$); 26.91 ($CH_3CH_2CH_2CH_2\mathbf{C}H_2$); 28.37 ($CH_3CH_2CH_2\mathbf{C}H_2CH_2$); 30.98 ($CH_3CH_2\mathbf{C}H_2CH_2CH_2$); 40.73 ($CH_2$-2′); 55.22 ($CH_3O$-DMTr); 63.79 ($CH_2$-5′); 70.78 (CH-3′); 84.58 (CH-1′); 85.47 (CH-4′); 85.91 (C-DMTr); 106.29 (C-5); 113.37 (CH-*m*-C_6H_4OMe-DMTr); 126.96 (CH-*p*-C_6H_5-DMTr); 127.93 (CH-*o*-C_6H_5-DMTr); 128.04 (CH-*m*-C_6H_5-DMTr); 129.88, 129.90 (CH-*o*-C_6H_4OMe-DMTr); 135.59, 135.62 (C-*i*-C_6H_4OMe-DMTr); 137.48 (CH-6); 144.79 (C-*i*-C_6H_5-DMTr); 155.00 (C-2); 158.32, 158.33 (C-*p*-C_6H_4OMe-DMTr); 164.87 (C-4).

HR ESI-MS calculated m/z: 600.30681 $[M+H]^+$, 622.28876 $[M+Na]^+$, found m/z: 600.30650 $[M+H]^+$, 622.28864 $[M+Na]^+$.

***N*[4]-Dimethylformimidamide-5'-*O*-[bis(4-methoxyphenyl)phenylmethyl]-5-(pent-1-yl)-2′-deocytidine (6b)**

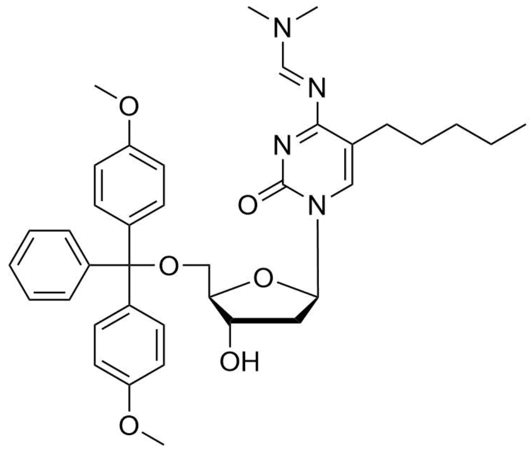

To the amino nucleoside precursor **5b** (8.1 g, 13.5 mmol) dissolved in dry DMF (135 mL) under argon atmosphere, dimethylformamide dimethylacetal (DMF-DMA, 25.2 mL, 189.1 mmol) was added and the reaction was stirred for 4 hours at 40 C. Subsequently, the solvent was evaporated and the crude product was purified by HPFC (DCM/MeOH) to obtain pure compound **6b** (8.1 g, 92%) in a form of a white foam.

^{1}H NMR (500.2 MHz, DMSO-d_6): .89 (t, 3H, $J_{CH3,CH2}$ = 7.2 Hz, C**H$_3$**CH$_2$CH$_2$CH$_2$CH$_2$); 0.95 – 1.35 (m, 6H, CH$_3$C**H$_2$**C**H$_2$**C**H$_2$**CH$_2$); 1.90 and 2.08 (2 × ddd, 2 × 1H, J_{gem} = 13.5, $J_{CH2,CH2}$ = 10.0 and 5.7 Hz, CH$_3$CH$_2$CH$_2$CH$_2$C**H$_2$**); 2.11 (dt, 1H, J_{gem} = 13.4 Hz, $J_{2'b,1'}$ = $J_{2'b,3'}$ = 6.8 Hz, H-2′a); 2.24 (ddd, 1H, J_{gem} = 13.3 Hz, $J_{2'b,1'}$ = 6.2 Hz, $J_{2'b,3'}$ = 3.7 Hz, H-2′b); 3.04 and 3.17 (2 × s, 2 × 3H, (CH$_3$)$_2$N); 3.20 (dd, 1H, J_{gem} = 10.6 Hz, $J_{5'a,4'}$ = 4.2 Hz, H-5′a); 3.23 (dd, 1H, J_{gem} = 10.6 Hz, $J_{5'b,4'}$ = 2.9 Hz, H-5′b); 3.73 (s, 2 × 3H, CH$_3$O-DMTr); 3.92 (q, 1H, $J_{4',5'b}$ = $J_{4',5'a}$ = $J_{4',3'}$ = 3.6 Hz, H-4′); 4.32 (dq, 1H, $J_{3',2'a}$ = 7.7 Hz, $J_{3',2'b}$ = $J_{3',4'}$ = $J_{3',OH}$ = 3.9 Hz, H-3′); 5.32 (d, 1H, $J_{OH,3'}$ = 4.5 Hz, OH-3′); 6.24 (t, 1H, $J_{1',2'a}$ = $J_{1',2'b}$ = 6.6 Hz, H-1′); 6.86 – 6.90 (m, 4H, H-*m*-C$_6$H$_4$OMe-DMTr); 7.23 (m, 1H, H-*p*-C$_6$H$_5$-DMTr); 7.24 – 7.29 (m, 4H, H-*o*-C$_6$H$_4$OMe-DMTr); 7.28 – 7.33 (m, 2H, H-*m*-C$_6$H$_5$-DMTr); 7.37 – 7.41 (m, 2H, H-*o*-C$_6$H$_5$-DMTr); 7.60 (s, 1H, H-6); 8.59 (m, 1H, NCH=N).

^{13}C NMR (125.8 MHz, DMSO-d_6): 14.02 (**C**H$_3$CH$_2$CH$_2$CH$_2$CH$_2$); 21.84 (CH$_3$**C**H$_2$CH$_2$CH$_2$CH$_2$); 27.84 (CH$_3$CH$_2$CH$_2$CH$_2$**C**H$_2$); 29.10 (CH$_3$CH$_2$CH$_2$**C**H$_2$CH$_2$); 31.26 (CH$_3$CH$_2$**C**H$_2$CH$_2$CH$_2$); 34.75 and 40.90 ((CH$_3$)$_2$N); 41.01 (CH$_2$-2′); 55.22 (CH$_3$O-DMTr); 63.70 (CH$_2$-5′); 70.71 (CH-3′); 85.15 (CH-1′); 85.72 (CH-4′); 86.01 (C-DMTr); 113.40 (CH-*m*-C$_6$H$_4$OMe-DMTr); 113.83 (C-5); 126.99 (CH-*p*-C$_6$H$_5$-DMTr); 127.95 (CH-*o*-C$_6$H$_5$-DMTr); 128.06 (CH-*m*-C$_6$H$_5$-DMTr); 129.90 (CH-*o*-C$_6$H$_4$OMe-DMTr); 135.59 and 135.65 (C-*i*-C$_6$H$_4$OMe-DMTr); 138.42 (CH-6); 144.78 (C-*i*-C$_6$H$_5$-DMTr); 155.10 (C-2); 157.57 (NCH=N); 158.36 (C-*p*-C$_6$H$_4$OMe-DMTr); 170.07 (C-4).

HR ESI-MS calculated m/z: 655.34901 $[M+H]^+$, 677.33096 $[M+Na]^+$, found m/z: 655.34880 $[M+H]^+$, 677.33078 $[M+Na]^+$.

N^4-Dimethylformimidamide-5'-*O*-[bis(4-methoxyphenyl)phenylmethyl]-5-(pent-1-yl)-2′-deocytidine-3′-(2-cyanoethyl *N,N*-diisopropylphosphoramidite) (2b)

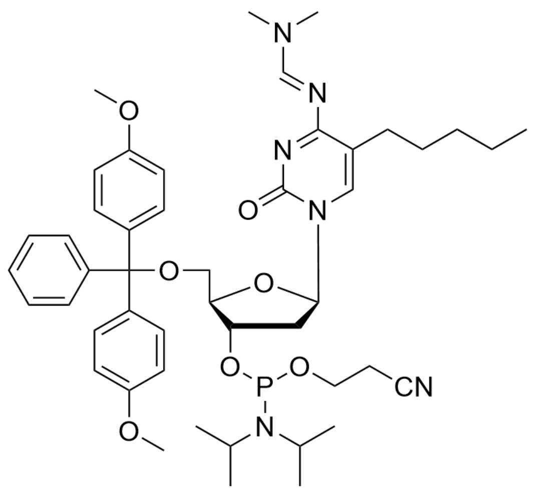

Compound **2b** was synthesized using General method C. Upon dissolution of protected nucleoside **6b** (1.0 g, 1.5 mmol) was in dry DCM (6 mL), DIPEA (665 μL, 3.8 mmol) was added, followed by 2-cyanoethyl-*N*,*N*-diisopropylchlorophosphoramidite (409 μL, 1.8 mmol). After the completion of the reaction (1.5 h), the final compound **2b** was acquired as two diastereomers (706 mg, 54%) in a form of a white foam.

^{1}H NMR (500.0 MHz, CD_3CN): 0.77 (t, 2×3H, $J_{CH3,CH2}$ = 7.3 Hz, $C\mathbf{H_3}CH_2CH_2CH_2CH_2$); 1.03,1.14 and 1.16 (4×d, 4×6H, $J_{CH3,CH}$ = 6.8 Hz, $(C\mathbf{H_3})_2CHN$); 1.02 – 1.09 (m, 2×2H, $CH_3CH_2C\mathbf{H_2}CH_2CH_2$); 1.10 – 1.19 (m, 2×2H, $CH_3C\mathbf{H_2}CH_2CH_2CH_2$); 1.24 – 1.41 (m, 2×2H, $CH_3CH_2CH_2C\mathbf{H_2}CH_2$); 1.95 – 2.06 and 2.13 -2.23 (2×m, 2×2H, $CH_3CH_2CH_2CH_2C\mathbf{H_2}$); 2.23 – 2.32 and 2.41 - 2.53 (m, 2×2H, H-2′); 2.51 and 2.64 (2×t, 2×2H, $J_{CH2,CH2}$ = 6.0 Hz, $OCH_2C\mathbf{H_2}CN$); 3.07 and 3.14 (2×s, 2×6H, $(C\mathbf{H_3})_2NCH$); 3.27 and 3.28 (2×dd, 2×1H, J_{gem} = 10.6 Hz, $J_{5'a,4'}$ = 3.7 and 4.1 Hz, H-5′a); 3.37 and 3.40 (2×dd, 2×1H, J_{gem} = 10.6 Hz, $J_{5'b,4'}$ = 2.7 Hz, H-5′b); 3.49 -3.83 (m, 8H, $(CH_3)_2C\mathbf{H}N$, $OC\mathbf{H_2}CH_2CN$); 3.75 and 3.76 (2×s, 2×6H, CH_3O-DMTr); 4.08 and 4.11 (2×bq, 2×1H, $J_{4',3'} = J_{4',5'}$ = 3.6 and 3.4 Hz, H-4′); 4.53 – 4.65 (m, 2×1H, H-3′); 6.27 and 6.29 (2×t, 2×1H, $J_{1',2'}$ = 6.5 and 6.6 Hz, H-1′); 6.83 – 6.88 (m, 2×4H, H-*m*-C_6H_4OMe-DMTr); 7.20 - 7.26 (m, 2×1H, H-*p*-C_6H_5-DMTr); 7.27 – 7.36 (m, 2×6H, H-*m*-C_6H_5-DMTr, H-*o*-C_6H_4OMe-DMTr); 7.42 – 7.47 (m, 2×2H, H-*o*-C_6H_5-DMTr); 7.60 and 7.64 (2×s, 2×1H, H-6); 8.63 (bs, 2×1H, $(CH_3)_2NC\mathbf{H}$).

^{13}C NMR (125.7 MHz, CD_3CN): 14.35 ($\mathbf{C}H_3CH_2CH_2CH_2CH_2$); 20.94 and 21.02 (2×d, $J_{C,P}$ = 7.1 and 7.2 Hz, $OCH_2\mathbf{C}H_2CN$); 23.05 ($CH_3\mathbf{C}H_2CH_2CH_2CH_2$); 24.78 – 24.92 (m, $(\mathbf{C}H_3)_2CHN$); 28.91 and 29.02 ($CH_3CH_2CH_2CH_2\mathbf{C}H_2$); 30.26 and 30.31 ($CH_3CH_2CH_2\mathbf{C}H_2CH_2$); 32.41 and 32.43 ($CH_3CH_2\mathbf{C}H_2CH_2CH_2$); 35.29 ($(\mathbf{C}H_3)_2NCH$); 40.95 and 41.11 (2×d, $J_{C,P}$ = 4.4 and 3.5 Hz, CH_2-2′); 41.57 ($(\mathbf{C}H_3)_2NCH$); 43.98 (d, $J_{C,P}$ = 12.4 Hz, $(CH_3)_2\mathbf{C}HN$); 55.90 and 55.91 (CH_3O-DMTr); 59.43 and 59.45 (2×d, $J_{C,P}$ = 19.0 and 19.1 Hz, $O\mathbf{C}H_2CH_2CN$); 63.87 and 64.15 (CH_2-5′); 73.70 and 74.35 (2×d, $J_{C,P}$ = 16.3 and 17.4 Hz, CH-3′); 85.81 and 86.01 (2×d, $J_{C,P}$ = 6.0 and 4.3 Hz, CH-4′); 86.53

(CH-1′); 87.34 and 87.35 (C-DMTr); 114.08, 114.09 and 114.10 (CH-*m*-C_6H_4OMe-DMTr); 115.53 and 115.58 (C-5); 119.40 and 119.56 (OCH_2CH_2**C**N); 127.94 and 127.96 (CH-*p*-C_6H_5-DMTr); 128.90 (CH-*o*-C_6H_5-DMTr); 129.06 and 129.13 (CH-*m*-C_6H_5-DMTr); 131.05, 131.07 and 131.08 (CH-*o*-C_6H_4OMe-DMTr); 136.71, 136.72, 136.76 and 136.78 (C-*i*-C_6H_4OMe-DMTr); 139.31 and 139.32 (CH-6); 145.83 and 145.84 (C-*i*-C_6H_5-DMTr); 156.73 (C-2); 158.49 and 158.50 ($(CH_3)_2$N**C**H); 159.73 (C-*p*-C_6H_4OMe-DMTr); 171.65 (C-4).

^{31}P{^{1}H} NMR (202.4 MHz, CD_3CN): 149.16 and 149.22 (2×s, 2×1P).

HR ESI-MS calculated m/z: 855.45686 $[M+H]^+$, 877.43881 $[M+Na]^+$, found m/z: 855.45655 $[M+H]^+$, 877.43811 $[M+Na]^+$.

1.7 Synthesis of Modified 2′-Deoxyadenosine

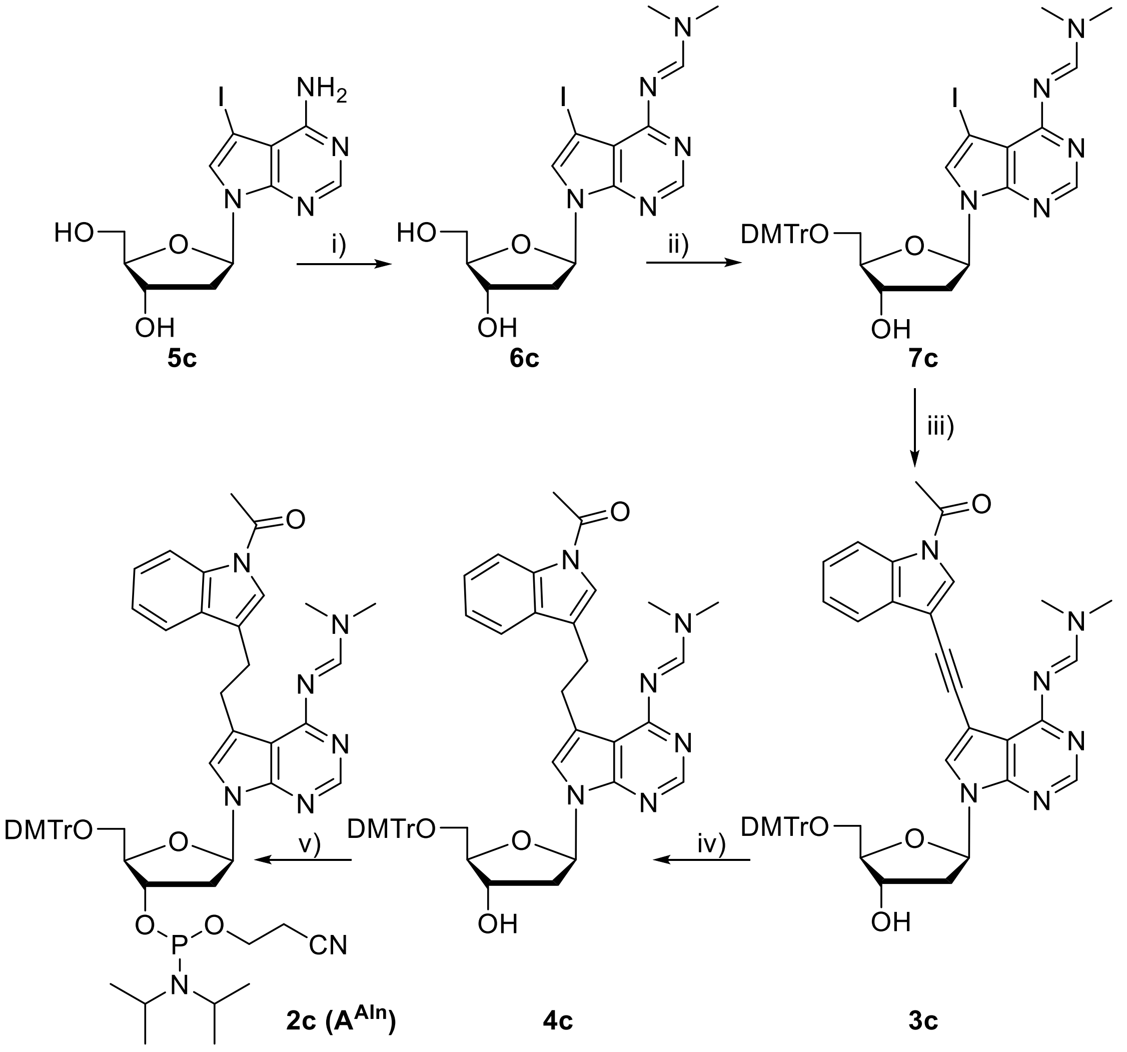

Scheme S3 Reagents and conditions: i) *N,N*-dimethylformamide dimethylacetal (14 equiv.), DMF (dry), 40°C, Ar, 4 h; ii) DMTrCl (1.3 equiv.), DMAP (0.1 equiv.), pyridine (dry), RT, overnight; iii) 1-acetyl-3-ethynylindole (1.3 equiv.), $(PPh_3)_2PdCl_2$ (0.2 equiv.), CuI (0.4 equiv.),

TEA (5 equiv.), DMF, RT, Ar, overnight; iv) H_2, 10% Pd/C (1 equiv.), MeOH, iPrOH, RT, 3 h; v) 2-cyanoethyl-*N,N*-diisopropylchlorophosphoramidite (1.5 equiv.), DIPEA (2.5 equiv.), DCM (dry), 0 °C to RT, 1.5 h;

N[6]-Dimethylformimidamide-5'-*O*-[bis(4-methoxyphenyl)phenylmethyl]-7-(1-acetyl-1*H*-indol-3-yl)ethyl)-2′-deoxy-7-deazaadenosine (4c)

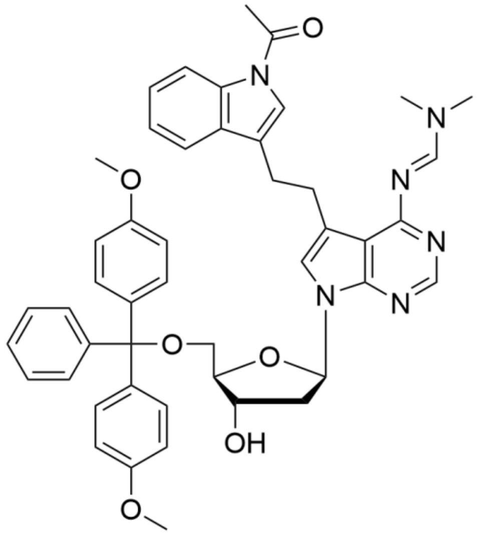

Compound **4c** was synthesized using General method B by dissolving compound **3c** (691 mg, 0.87 mmol) in MeOH and iPrOH and adding 10% Pd/C (926 mg, 0.87 mmol). Product **4c** was isolated as a brownish foam (538 mg, 77%).

^{1}H NMR (500.0 MHz, DMSO-d_6): 2.25 (ddd, 1H, Jgem = 13.1 Hz, J2′a,1′ = 6.3 Hz, J2′a,3′ = 3.6 Hz, H-2′a); 2.56 (s, 3H, CH3CO); 2.56 (m, 1H, H-2′b); 2.84 – 2.96 (m, 2H, CH2CH2-indolyl); 2.96 and 3.13 (2×s, 2×3H, (CH3)2N); 3.11 – 3.20 (m, 4H, H-5′, CH2CH2-indolyl); 3.65 and 3.66 (2×s, 2×3H, CH3O-DMTr); 3.93 (m, 1H, H-4′); 4.42 (m, 1H, H-3′); 5.34 (d, 1H, JOH,3′ = 4.4 Hz, OH-3′); 6.61 (bt, 1H, J1′,2′b = J1′,2′a = 6.8 Hz, H-1′); 6.78 – 6.85 (m, 4H, H-m-C6H4OMe-DMTr); 7.16 – 7.21 (m, 2H, H-p-C6H5-DMTr, CH-5-indolyl); 7.21 – 7.28 (m, 7H, H-m-C6H5-DMTr, H-o-C6H4OMe-DMTr, H-6); 7.29 (m, 1H, H-6-indolyl); 7.34 – 7.41 (m, 3H, H-o-C6H5-DMTr, H-4-indolyl); 7.51 (s, 1H, H-2-indolyl); 8.29 (d, 1H, J7,6 = 8.2 Hz, H-7-indolyl), 8.30 (CH-2); 8.85 (s, 1H, NCH=N).

^{13}C NMR (125.7 MHz, DMSO-d_6): 23.76 (CH3CO); 25.67 (CH2CH2-indolyl); 26.49 (CH2CH2-indolyl); 34.36 ((CH3)2N); 39.87 (CH2-2′); 40.02 ((CH3)2N); 54.94 and 54.95 (CH3O-DMTr); 64.22 (CH2-5′); 71.03 (CH-3′); 82.24 (CH-1′); 85.24 (CH-4′); 85.60 (C-DMTr); 109.93 (C-4a); 113.12 and 113.15 (CH-m-C6H4OMe-DMTr); 115.86 (CH-7-indolyl); 116.59 (C-5); 119.00 (CH-4-indolyl); 120.15 (CH-6); 121.97 (C-3-indolyl); 122.76 (CH-2-indolyl); 123.00 (CH-5-indolyl); 124.66 (CH-6-indolyl); 126.69 (CH-p-C6H5-DMTr); 127.79 (CH-o,m-C6H5-DMTr); 129.72 and 129.77 (CH-o-C6H4OMe-DMTr); 130.46 (C-3a-indolyl); 135.11 (C-7a-indolyl); 135.55 and 135.59 (C-i-C6H4OMe-DMTr); 144.81 (C-i-C6H5-DMTr); 151.07 (CH-2); 151.85 (C-7a); 156.40 (NCH=N); 158.03 and 158.06 (C-p-C6H4OMe-DMTr); 160.67 (C-4); 169.01 (CH3CO).

HR ESI-MS calculated m/z: 793.37081 $[M+H]^+$, 815.35275 $[M+Na]^+$, found m/z: 793.37108 $[M+H]^+$, 815.35312 $[M+Na]^+$.

***N*[6]-Dimethylformimidamide-5'-*O*-[bis(4-methoxyphenyl)phenylmethyl]-7-(1-acetyl-1*H*-indol-3-yl)ethyl)-2′-deoxy-7-deazaadenosine-3′-(2-cyanoethyl *N,N*-diisopropylphosphoramidite) (2c)**

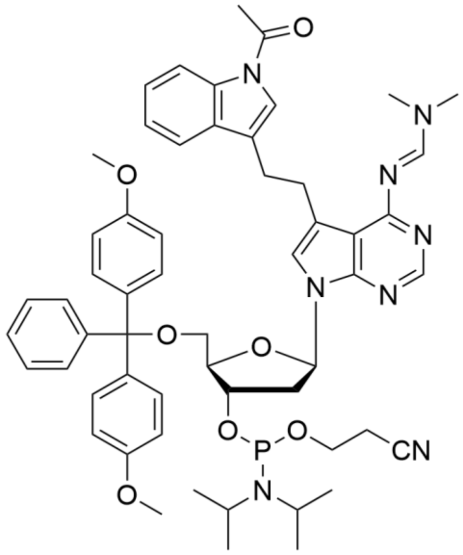

Protected nucleoside **4c** (492 mg, 0.62 mmol) was reacted to the corresponding phosphoramidite using General method C. Freshly distilled DIPEA (270 μL, 1.55 mmol) and subsequently 2-cyanoethyl-*N*,*N*-diisopropylchlorophosphoramidite (166 μL, 0.74 mmol) were injected. Purification provided final compound **2c** as diastereomers (254 mg, 41%) in a form of a white foam.

^{1}H NMR (500.0 MHz, CD_3CN): 1.08, 1.16 and 1.18 (4×d, 4×3H, JCH3,CH = 6.8 Hz, (CH3)2CHN); 2.48 and 2.49 (2×s, 2×3H, CH3CO); 2.40 – 2.55 (m, 2×1H, H-2′a); 2.53 and 2.65 (2×t, 2×2H, JCH2,CH2 = 6.0 Hz, OCH2CH2CN); 2.67 – 2.79 (m, 2×1H, H-2′b); 2.85 – 3.00 (m, 2×2H, CH2CH2-indolyl); 2.96, 2.97 and 3.09 (4×s, 4×3H, (CH3)2N); 3.05 – 3.35 (m, 8H, H-5′, CH2CH2-indolyl); 3.668, 3.673 and 3.678 (4×s, 4×3H, CH3O-DMTr); 3.51 – 3.88 (m, 8H, (CH3)2CHN, OCH2CH2CN); 4.09 and 4.12 (2×m, 2×1H, H-4′); 4.75 – 4.84 (m, 2×1H, H-3′); 6.62 and 6.63 (2×t, 2×1H, J1′,2′b = J1′,2′a = 6.7 Hz, H-1′); 6.72 – 6.80 (m, 2×4H, H-m-C6H4OMe-DMTr); 7.12 (s, 2×1H, H-6); 7.13 – 7.35 (m, 22H, H-o,p-C6H5-DMTr, H-o-C6H4OMe-DMTr, H-2,3,5,6-indolyl); 7.38 – 7.44 (m, 2×2H, H-m-C6H5-DMTr); 8.30 and 8.31 (2×s, 2×1H, H-2); 8.32 (m, 2×1H, H-7-indolyl); 8.80 (s, 2×1H, NCH=N).

^{13}C NMR (125.7 MHz, CD_3CN): 20.98 and 21.06 (d, JC,P = 7.1 Hz, OCH2CH2CN); 24.34 (CH3CO); 24.82 – 24.95 (m, (CH3)2CHN); 26.70 and 26.73 (CH2CH2-indolyl); 27.50 and 27.51 (CH2CH2-indolyl); 35.10 ((CH3)2N); 39.79 and 39.88 (d, JC,P = 4.3 and 3.4 Hz, CH2-2′); 41.18 ((CH3)2N); 43.99 (d, JC,P = 12.4 Hz, (CH3)2CHN); 55.80 and 55.81 (CH3O-DMTr); 59.39 and 59.45 (d, JC,P = 19.0 Hz, OCH2CH2CN); 64.53 and 64.65 (CH2-5′); 74.28 and 74.75 (d, JC,P = 16.5 and 17.4 Hz, CH-3′); 83.67 and 83.72 (CH-1′); 85.39 and 85.59 (d, JC,P = 5.9 and 4.1 Hz, CH-4′); 87.11 and 87.12 (C-DMTr); 111.46 and 111.48 (C-4a); 113.96 (CH-m-C6H4OMe-DMTr); 117.02 (CH-7-

indolyl); 118.35 and 118.37 (C-5); 119.43 and 119.59 (OCH2CH2CN); 120.01 and 120.03 (CH-4-indolyl); 121.10 and 121.13 (CH-6); 123.45 and 123.47 (C-3-indolyl); 123.54 nad 123.56 (CH-2-indolyl); 123.97 (CH-5-indolyl); 125.65 (CH-6-indolyl); 127.80 and 127.82 (CH-p-C6H5-DMTr); 128.75 and 128.77 (CH-m-C6H5-DMTr); 129.10 and 129.15 (CH-o-C6H5-DMTr); 131.00, 131.03 and 131.06 (CH-o-C6H4OMe-DMTr); 131.84 (C-3a-indolyl); 136.65 (C-7a-indolyl); 136.83, 136.88 and 136.92 (C-i-C6H4OMe-DMTr); 145.97 (C-i-C6H5-DMTr); 152.44 (CH-2); 153.41 (C-7a); 157.32 (NCH=N); 159.58 and 158.60 (C-p-C6H4OMe-DMTr); 162.17 (C-4); 169.94 (CH3CO).
^{31}P NMR: 149.23 and 149.12 (2×s, 2×1P).
HR ESI-MS: calculated m/z: 993.47866 $[M+H]^+$, 1015.46060 $[M+Na]^+$ found m/z: 993.47924 $[M+H]^+$, 1015.46079 $[M+Na]^+$.

1.8 Synthesis of Modified 2′-Deoxyguanosine

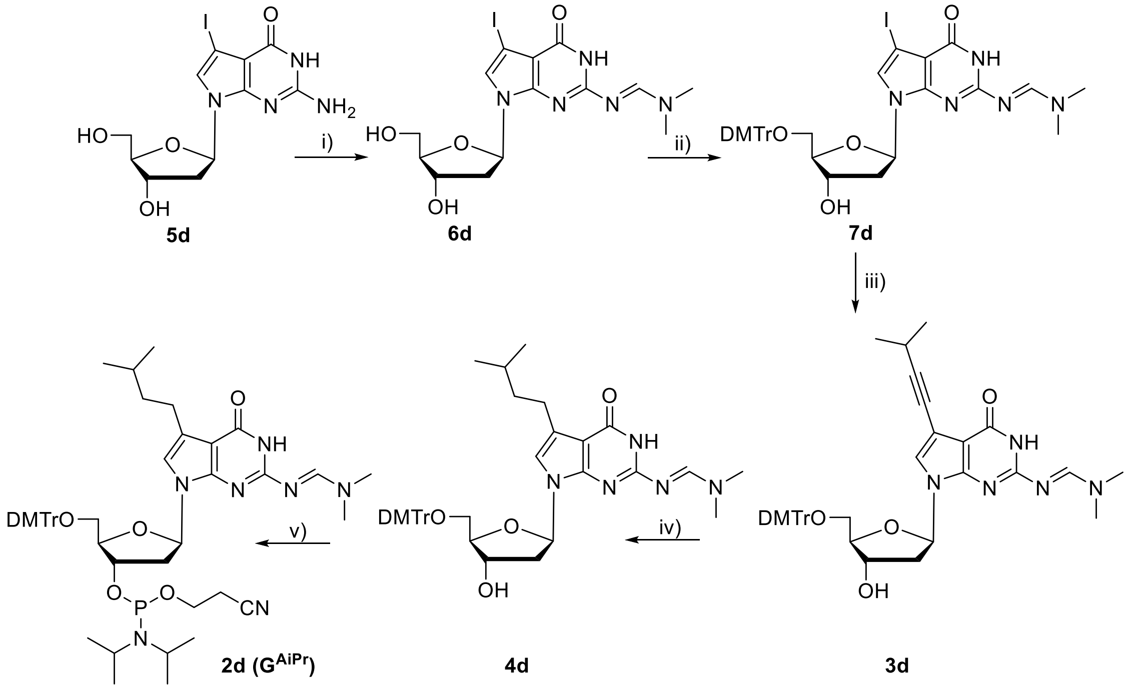

Scheme S4 Reagents and conditions: i) *N,N*-dimethylformamide dimethylacetal (14 equiv.), DMF (dry), 40°C, Ar, 4 h; ii) DMTrCl (1.3 equiv.), DMAP (0.1 equiv.), pyridine (dry), RT, overnight; iii) 3-methylbut-1-yne (25 equiv.), $(PPh_3)_2PdCl_2$ (0.2 equiv.), CuI (0.4 equiv.), TEA (5 equiv.), , DMF, RT, Ar, overnight; iv) H_2, 10% Pd/C (1 equiv.), MeOH, iPrOH, RT, 3 h; v) 2-cyanoethyl-*N,N*-diisopropylchlorophosphoramidite (1.5 equiv.), DIPEA (2.5 equiv.), DCM (dry), 0 °C to RT, 1.5 h;

N^2-Dimethylformimidamide-5'-*O*-[bis(4-methoxyphenyl)phenylmethyl]-7-isopentyl-2′-deoxy-7-deazaguanosine (4d)

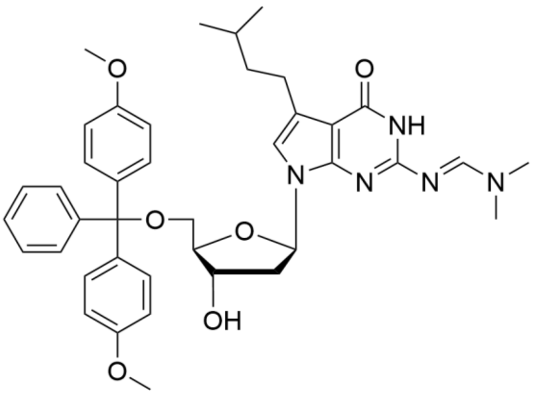

General method B was used to synthesize compound **4d** by dissolving compound **3d** (500 mg, 0.72 mmol) in MeOH (20 mL), adding 10% Pd/C (154 mg, 0.14 mmol), and exchanging the atmosphere for H_2. Product **4d** was isolated as a brownish foam (445 mg, 89%).

^{1}H NMR (500.0 MHz, DMSO-d$_6$): 0.76 and 0.78 (2×d, 2×3H, $J_{CH3,CH}$ = 6.6 Hz, (C**H$_3$**)$_2$CHCH$_2$CH$_2$); 1.27 – 1.37 (m, 2H, (CH$_3$)$_2$CHC**H$_2$**CH$_2$); 1.43 (m, 1H, (CH$_3$)$_2$C**H**CH$_2$CH$_2$); 2.19 (ddd, 1H, J_{gem} = 13.3 Hz, $J_{2'a,1'}$ = 6.2 Hz, $J_{2'a,3'}$ = 3.2 Hz, H-2′a); 2.52 (bddd, 1H, J_{gem} = 13.4 Hz, $J_{2'b,1'}$ = 7.8 Hz, $J_{2'b,3'}$ = 6.7 Hz, H-2′b); 2.43 - 2.57 (m, 2H, (CH$_3$)$_2$CHCH$_2$C**H$_2$**); 3.01 (s, 3H, (CH$_3$)$_2$N); 3.11 (dd, 1H, J_{gem} = 10.3 Hz, $J_{5'a,4'}$ = 3.3 Hz, H-5′a); 3.13 (s, 3H, (CH$_3$)$_2$N); 3.15 (dd, 1H, J_{gem} = 10.3 Hz, $J_{5'b,4'}$ = 5.0 Hz, H-5′a); 3.72 (2×s, 2×3H, CH$_3$O-DMTr); 3.90 (m, 1H, H-4′); 4.37 (dq, 1H, $J_{3',2'b}$ = 6.7 Hz, $J_{3',2'a}$ = $J_{3',4'}$ = $J_{3',OH}$ = 3.3 Hz, H-3′); 5.38 (bd, 1H, $J_{OH,3'}$ = 4.0 Hz, OH-3′); 6.46 (dd, 1H, $J_{1',2'b}$ = 7.8 Hz, $J_{1',2'a}$ = 6.2 Hz, H-1′); 6.65 (s, 1H, H-6); 6.81 – 6.89 (m, 4H, H-*m*-C$_6$H$_4$OMe-DMTr); 7.21 (m, 1H, H-*p*-C$_6$H$_5$-DMTr); 7.23 – 7.32 (m, 6H, H-*m*-C$_6$H$_5$-DMTr, H-*o*-C$_6$H$_4$OMe-DMTr); 7.39 (m, 2H, H-*m*-C$_6$H$_5$-DMTr); 8.55 (s, 1H, NCH=N); 10.86 (bs, 1H, NH).

^{13}C NMR (125.7 MHz, DMSO-d$_6$): 22.38 and 22.40 ((**C**H$_3$)$_2$CHCH$_2$CH$_2$); 23.67 ((CH$_3$)$_2$CHCH$_2$**C**H$_2$); 27.21 ((CH$_3$)$_2$**C**HCH$_2$CH$_2$); 34.58 ((CH$_3$)$_2$N); 40.15 (CH$_2$-2′); 40.52 ((CH$_3$)$_2$N); 55.01 (CH$_3$O-DMTr); 64.36 (CH$_2$-5′); 71.15 (CH-3′); 81.87 (CH-1′); 85.20 (CH-4′); 85.61 (C-DMTr); 101.93 (C-4a); 113.12 (CH-*m*-C$_6$H$_4$OMe-DMTr); 114.06 (CH-6); 119.93 (C-5); 126.67 (CH-*p*-C$_6$H$_5$-DMTr); 127.79 (CH-*o*,*m*-C$_6$H$_5$-DMTr); 129.74 (CH-*o*-C$_6$H$_4$OMe-DMTr); 135.50 and 135.61 (C-*i*-C$_6$H$_4$OMe-DMTr); 144.80 (C-*i*-C$_6$H$_5$-DMTr); 149.43 (C-7a); 155.73 (C-2); 157.22 (NCH=N); 158.06 (C-*p*-C$_6$H$_4$OMe-DMTr); 159.85 (C-4).

HR ESI-MS calculated m/z: 694.35991 $[M+H]^+$, 716.34186 $[M+Na]^+$, found m/z: 694.36060 $[M+H]^+$, 716.34229 $[M+Na]^+$.

N^2-Dimethylformimidamide-5'-*O*-[bis(4-methoxyphenyl)phenylmethyl]-7-isopentyl-2′-deoxy-7-deazaguanosine-3′-(2-cyanoethyl *N,N*-diisopropylphosphoramidite) (2d)

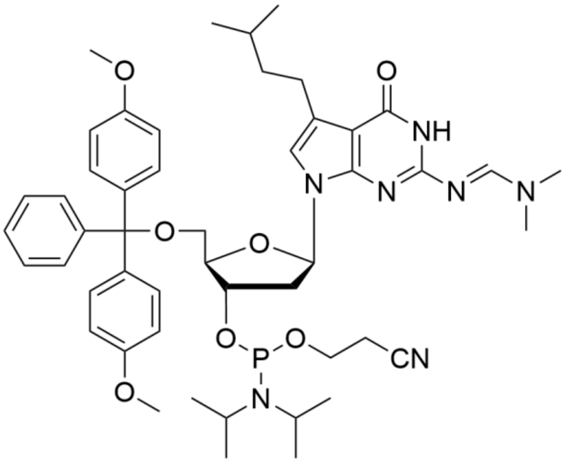

General method C was used to synthesize compound **2d**. Compound **4d** (435 mg, 0.63 mmol) was dissolved in dry DCM (4 mL), subsequently freshly distilled DIPEA (274 μL, 1.57 mmol) and 2-cyanoethyl-*N*,*N*-diisopropylchlorophosphoramidite (168 μL, 0.75 mmol) were added. After purification, target compound **2d** (318 mg, 57%) was obtained as a mixture of two diastereomers in a form of white foam. **^{1}H NMR** (500.0 MHz, CD_3CN): 0.81 and 0.83 (2×d, 4×6H, JCH3,CH = 6.6 Hz, (CH3)2CHCH2CH2); 1.05, 1.14 and 1.15 (4×d, 4×3H, JCH3,CH = 6.8 Hz, (CH3)2CHN); 1.35 – 1.45 (m, 2×2H, (CH3)2CHCH2CH2); 1.49 (2×m, 2×1H, (CH3)2CHCH2CH2); 2.31 – 2.45 (m, 2×1H, H-2′a); 2.54 and 2.64 (2×bt, 2×2H, JCH2,CH2 = 6.0 Hz, OCH2CH2CN); 2.49 – 2.68 (m, 6H, H-2′b, (CH3)2CHCH2CH2); 3.00, 3.074 and 3.068 (4×s, 4×3H, (CH3)2N); 3.12 – 3.28 (m, 2×2H, H-5′); 3.72 and 3.73 (4×s, 4×3H, CH3O-DMTr); 3.48 – 3.84 (m, 8H, (CH3)2CHN, OCH2CH2CN); 4.04 and 4.08 (2×m, 2×1H, H-4′); 4.59 – 4.78 (m, 2×1H, H-3′); 6.49 (2×bt, 2×1H, J1′,2′b = J1′,2′a = 6.8 Hz, H-1′); 6.62 and 6.63 (2×s, 2×1H, H-6); 6.76 – 6.85 (m, 2×4H, H-m-C6H4OMe-DMTr); 7.13 – 7.33 (m, 14H, H-o,p-C6H5-DMTr, H-o-C6H4OMe-DMTr); 7.37 – 7.45 (m, 2×2H, H-m-C6H5-DMTr); 857 and 8.58 (2×s, 2×1H, NCH=N); 9.36 (bs, 2×1H, NH). **^{13}C NMR** (125.7 MHz, CD_3CN): 20.98 and 21.05 (d, JC,P = 7.1 Hz, OCH2CH2CN); 22.88 ((CH3)2CHCH2CH2); 24.84 ((CH3)2CHCH2CH2); 24.87 – 24.96 (m, (CH3)2CHN); 28.53 ((CH3)2CHCH2CH2); 35.10 ((CH3)2N); 40.09 and 40.19 (d, JC,P = 4.4 and 3.3 Hz, CH2-2′); 40.31 ((CH3)2CHCH2CH2); 41.04 ((CH3)2N); 44.04 (d, JC,P = 12.4 Hz, (CH3)2CHN); 55.86 and 55.88 (CH3O-DMTr); 59.46 and 59.50 (d, JC,P = 19.0 Hz, OCH2CH2CN); 64.78 and 64.93 (CH2-5′); 74.66 and 75.00 (d, JC,P = 16.8 and 17.6 Hz, CH-3′); 83.48 and 83.52 (CH-1′); 85.36 and 85.58 (d, JC,P = 5.9 and 4.4 Hz, CH-4′); 87.14 and 87.17 (C-DMTr); 103.38 and 103.41 (C-4a); 114.00 (CH-m-C6H4OMe-DMTr); 115.42 and 115.46 (CH-6); 119.42 and 119.56 (OCH2CH2CN); 121.99 (C-5); 127.81 and 127.84 (CH-p-C6H5-DMTr); 128.80 and 128.81 (CH-m-

C6H5-DMTr); 129.04 and 129.09 (CH-o-C6H5-DMTr); 131.01 and 131.04 (CH-o-C6H4OMe-DMTr); 136.78, 136.84, 136.87 and 136.91 (C-i-C6H4OMe-DMTr); 146.01 (C-i-C6H5-DMTr); 151.03 and 151.07 (C-7a); 156.80 (C-2); 158.49 and 158.53 (NCH=N); 159.64 (C-p-C6H4OMe-DMTr); 160.96 (C-4).

^{31}P NMR (202.4 MHz, CD_3CN): 149.14 and 149.11 (2×s, 2×1P).

HR ESI-MS calculated m/z: 894.46776 $[M+H]^+$, 916.44970 $[M+Na]^+$, found m/z: 894.46814 $[M+H]^+$, 916.44922 $[M+Na]^+$.

2 Synthesis of Modified Oligonucleotides

2.1 Solid-phase Synthesis

Solid-phase synthesis of partially and hyper-modified oligonucleotides **ON1$^{\mathbf{AIn}}$–ON12$^{\mathbf{A*}}$** using the phosphoramidites **2a**, **2b**, **2c** and **2d** was performed in a 1 μmole scale. The trityl-off mode was used to prevent potential loss of the products in another purification round, moreover to avoid the risk of a strong attachment to the reverse phase, and also possible problems with the elution due to the enhanced hydrophobicity of the modified ONs. Standard solid-phase columns were used for partially modified **ON1$^{\mathbf{AIn}}$–ON8$^{\mathbf{A*}}$**, and universal solid-phase columns were used for hyper-modified **ON9$^{\mathbf{A*}}$**, **ON10$^{\mathbf{A*}}$**. Each phosphoramidite, non-modified or modified, was diluted to a 0.1 M solution. As an activator, 0.3 M 5-(benzylthio)-1*H*-tetrazole (BTT) solution in MeCN was used and as in the oxidation step iodine solution (0.02 M) in THF/pyridine/water (ratio 70:20:10) was used. For the unmodified as well as for the modified phosphoramidites standard cycle procedures provided by BioAutomation Corporation were applied. The duration and coupling volume for the natural phosphoramidites were 1 minute 30 seconds and 220 μL, however, for the modified phosphoramidite the coupling time was increased to 6 minutes to maximize the incorporation efficiency, keeping the volume the same. Cleavage from the solid-phase was done by 30% aqueous NH_3 for 45 minutes two times (2×1 mL). Subsequent deprotection step was carried out by incubation of the oligonucleotide solutions at 65 C for 6 hours.

2.2 Purification and Characterization

The purification of the oligonucleotides was performed using HPLC with a linear gradient of MeCN (0–100%) in 0.1 M triethylammonium bicarbonate (TEAB) buffer (pH 7.6). The chromatograms are provided in chapter 3 and retention times of the collected peaks are in **Table S1**.

Table S1 Retention times of oligonucleotides during HPLC purification (1 h gradient).

Code	Retention time
**ON1**$^{\mathbf{AIn}}$	19.191
**ON2**$^{\mathbf{AiPr}}$	18.899
**ON3**$^{\mathbf{AiPr}}$	26.265
**ON4**$^{\mathbf{APr}}$	18.059
**ON5**$^{\mathbf{APr}}$	22.960
**ON6**$^{\mathbf{APh}}$	18.101
**ON7**$^{\mathbf{APh}}$	25.613
**ON8**$^{\mathbf{A*}}$	27.398
**ON9**$^{\mathbf{A*}}$	65.713 (2 h grad.)
**ON10**$^{\mathbf{A*}}$	65.126 (2 h grad.)

Subsequent lyofilization from H_2O provided pure products. The approximate concentrations were measured by UV/VIS Spectrophotometer at 260 nm, mass and purity were measured on UHPLC-MS. Most of the modified ONs are >90% pure, except **ON10**$^{\mathbf{A*}}$ showing 88% purity. The exact concentrations and yields were measured by the elemental analysis of pure modified ONs based on the phosphorus content. The sequence, calculated and measured mass, purity and isolated yield of each synthesized oligonucleotide are shown in **Table S2**.

Table S2 List of the chemically synthesized modified oligonucleotides **ON1**$^{\mathbf{AIn}}$**–ON10**$^{\mathbf{A*}}$, their sequences, calculated and measured masses [Da], their purities [%] and isolated yields [%] measured by elemental analysis.

Code	Sequence (5′⟶3′)	Mass calc. [Da]	Mass found [Da]	Purity [%]	Yield[a] [%]
**ON1**$^{\mathbf{AIn}}$	ATCTCAG**A**$^{\mathbf{AIn}}$GAACTGC	4703.2	4702.7	98	27 (53)[a]
**ON2**$^{\mathbf{AiPr}}$	ATCTCAG**G**$^{\mathbf{AiPr}}$AAGCTGC	4646.2	4645.6	96	45
**ON3**$^{\mathbf{AiPr}}$	ATGTCA**G**$^{\mathbf{AiPr}}$**G**$^{\mathbf{AiPr}}$**G**$^{\mathbf{AiPr}}$AGCTGC	4840.5	4839.9	97	22 (41)[a]
**ON4**$^{\mathbf{APr}}$	GCTCCGT**C**$^{\mathbf{APr}}$GATTGAA	4638.1	4637.6	99	85
**ON5**$^{\mathbf{APr}}$	GCTCC**C**$^{\mathbf{APr}}$**C**$^{\mathbf{APr}}$**C**$^{\mathbf{APr}}$GATTGAA	4723.4	4722.9	>99	56
**ON6**$^{\mathbf{APh}}$	GCTCCGT**U**$^{\mathbf{APh}}$GATTGAA	4673.2	4671.9	91	76
**ON7**$^{\mathbf{APh}}$	GCTCCG**U**$^{\mathbf{APh}}$**U**$^{\mathbf{APh}}$**U**$^{\mathbf{APh}}$ATTGAA	4828.4	4827.0	>99	14 (80)[a]
**ON8**$^{\mathbf{A*}}$	GCTCG**C**$^{\mathbf{APr}}$**U**$^{\mathbf{APh}}$**C**$^{\mathbf{APr}}$**U**$^{\mathbf{APh}}$ATTGAA	4863.5	4862.9	>99	76
**ON9**$^{\mathbf{A*}}$	**G**$^{\mathbf{AiPr}}$**U**$^{\mathbf{APh}}$**A**$^{\mathbf{AIn}}$**G**$^{\mathbf{AiPr}}$**A**$^{\mathbf{AIn}}$**U**$^{\mathbf{APh}}$**G**$^{\mathbf{AiPr}}$**C**$^{\mathbf{Apr}}$ **A**$^{\mathbf{AIn}}$**C**$^{\mathbf{APr}}$**U**$^{\mathbf{APh}}$**C**$^{\mathbf{APr}}$**G**$^{\mathbf{AiPr}}$**U**$^{\mathbf{APh}}$**C**$^{\mathbf{APr}}$	5912.2	5910.1	96	8 (17)[a]
**ON10**$^{\mathbf{A*}}$	**G**$^{\mathbf{AiPr}}$**A**$^{\mathbf{AIn}}$**C**$^{\mathbf{APr}}$**G**$^{\mathbf{AiPr}}$**A**$^{\mathbf{AIn}}$**G**$^{\mathbf{AiPr}}$**U**$^{\mathbf{APh}}$**G**$^{\mathbf{AiPr}}$ **C**$^{\mathbf{APr}}$**A**$^{\mathbf{AIn}}$**U**$^{\mathbf{APh}}$**C**$^{\mathbf{APr}}$**U**$^{\mathbf{APh}}$**A**$^{\mathbf{AIn}}$**C**$^{\mathbf{APr}}$	5973.3	5971.2	88	7 (13)[a]

[a] HPLC yields given in parenthesis in case of lower isolated yield

2.3 Hybridization Experiments

To anneal modified **ON1$^{\mathbf{AIn}}$–ON10$^{\mathbf{A*}}$** with the complementary strands **cON1–cON9** (shown in **Table S3**) a solution of complementary single stranded ONs (50 μL, 5 μM each) with the annealing buffer 2X (20 mM Tris, pH 7.5–8.0, 100 mM NaCl, 2 mM EDTA) were heated on a thermo-block to 95°C for 5 min and then let cool down at RT. The formed **DNA1$^{\mathbf{AIn}}$–DNA11$^{\mathbf{A*}}$** (**Table 1**) were visualised on an agarose gel (3%) stained with GelRed and analyzed in 0.5X TBE buffer. The resulting gel can be seen on **Figure 2**.

Table S3 List of annealed double-stranded non-modified (DNA) prepared by annealing ON# with complementary **cON#** (# = 1–9).

Code		DNA
**DNA1**	ON1<br>cON1	5′-ATCTCAGAGAACTGC-3′<br>3′-TAGAGTCTCTTGACG-5′
**DNA2**	ON2<br>cON2	5′-ATCTCAGGAAGCTGC-3′<br>3′-TAGAGTC C TTCGACG-5′
**DNA3**	ON3<br>cON3	5′-ATGTCAGGGAGCTGC-3′<br>TACAGTCCCTCGACG-5′
**DNA4**	ON4<br>cON4	5′-GCTCCGTCGATTGAA-3′<br>3′-CGAGGCAGCTAACTT-5′
**DNA5**	ON5<br>cON5	5′-GCTCCCCCGATTGAA-3′<br>3′-CGAGGGGGCTAACTT-5′
**DNA6**	ON6<br>cON6	5′-GCTCCGTUGATTGAA-3′<br>3′-CGAGGCAACTAACTT-5′
**DNA7**	ON7<br>cON7	5′-GCTCCGUUUATTGAA-3′<br>3′-CGAGGCAAATAACTT-5′
**DNA8**	ON8<br>cON8	5′-GCTCGCUCUATTGAA-3′<br>3′-CGAGCGAGATAACTT-5′
**DNA9**	ON9<br>cON9	5′-GUAGAUGCACUCGUC-3′<br>3′-CATCTACGTGAGCAG-5′

2.4 Stability Analysis

The melting (T_m) and annealing temperatures (T_a), were measured by UV-vis spectroscopy and moreover with CD spectroscopy.

The results from UV-vis measurements, depicted in **Table S4** and **Table S5**, were recorded in 1 mm rectangular quartz cell in UV-vis Spectrophotometer, in a temperature ranging from 25 C till 95 C with the temperature increment 1 °C/min with detection at 260 nm and were obtained from three cycles (6 ramps in total). T_m and T_a values (in °C) were calculated using the first negative derivative of the intensity over the temperature.

Table S4 Melting (T_m, [°C]) and annealing (T_a, [°C]) temperatures of non-modified duplexes (DNA) and modified duplexes (DNA^{A*}) determined by UV-vis spectroscopy in 3 replicates with their standard deviations. ΔT_m and ΔT_a - the difference of T_m and T_a between modified DNA^{A*} and native DNA, and ΔT_m/modification - the difference per one modification.

**Code**	**T_a (260 nm) [°C]**				**T_m (260 nm) [°C]**			
	**DNA**	**DNA^{A*}**	**ΔT_a**	**ΔT_a/modification**	**DNA**	**DNA^{A*}**	**ΔT_m**	**ΔT_m/modification**
**$DNA1^{AIn}$**	46.3±0.42	45.3±0.67	-1.0	-1.0	51.8±0.53	46.3±1.22	-5.5	-5.5
**$DNA2^{AiPr}$**	50.9±0.61	49.0±0.52	-1.9	-1.9	55.9±0.48	51.5±0.31	-4.4	-4.4
**$DNA3^{AiPr}$**	55.7±0.48	50.0±0.58	-5.7	-1.9	59.8±0.53	51.6±0.19	-8.1	-2.7
**$DNA4^{APr}$**	52.0±0.43	51.5±0.10	-0.5	-0.5	57.3±0.58	52.1±0.57	-5.3	-5.3
**$DNA5^{APr}$**	53.3±0.44	51.1±0.31	-2.2	-0.7	57.4±0.36	52.8±0.46	-4.6	-1.5
**$DNA6^{APh}$**	48.6±0.59	45.1±0.18	-3.5	-3.5	53.3±0.15	45.8±0.67	-7.5	-7.5
**$DNA7^{APh}$**	44.5±0.45	36.5±0.35	-8.0	-2.7	48.5±0.95	37.3±0.07	-11.2	-3.7
**$DNA8^{A*}$**	47.8±0.13	39.0±0.48	-8.7	-2.2	52.3±0.61	41.5±0.39	-10.8	-2.7
**$DNA9^{A*}$**	52.5±0.14	ND[a]	ND[a]	ND[a]	54.9±0.60	ND[a]	ND[a]	ND[a]
**$DNA10^{A*}$**		ND[a]	ND[a]	ND[a]		ND[a]	ND[a]	ND[a]
**$DNA11^{A*}$**		ND[a]	ND[a]	ND[a]		ND[a]	ND[a]	ND[a]

[a] ND: not determined

Table S5 Comparison of melting temperatures (T_m, [°C]) of non-modified (DNA) and modified DNA duplexes (**$DNA^{A/E*}$**, ^{E} stands for ethynyl linker, ^{A} stands for alkyl/ethyl linker) determined by UV-vis spectroscopy at 260 nm and the difference of T_m between modified DNAs and non-modified DNAs per modification (**$DNA^{A/E*}$** ΔT_m/modification, $DNA^{A/E*}$ ΔT_m/mod.).

**Code**	**T_m (260 nm) [°C]**				
	**DNA**	**DNA^{A*}**	**DNA^{E*b}**	**DNA^{A*} ΔT_m/modification**	**DNA^{E*} ΔT_m/modification[b]**
**$DNA1^{A/EIn}$**	51.8±0.53	46.3±1.22	49.6±0.24	-5.5	-2.2
**$DNA2^{A/EiPr}$**	55.9±0.48	51.5±0.31	54.7±0.44	-4.4	-1.2
**$DNA3^{A/EiPr}$**	59.8±0.53	51.6±0.19	57.9±0.59	-2.7	-0.6
**$DNA4^{A/EPr}$**	57.3±0.58	52.1±0.57	57.5±0.99	-5.3	+0.2
**$DNA5^{A/EPr}$**	57.4±0.36	52.8±1.80	64.6±0.39	-1.5	+2.4
**$DNA6^{A/EPh}$**	53.3±0.15	45.8±0.67	52.2±0.13	-8.0	-1.1
**$DNA7^{A/EPh}$**	48.5±0.95	37.3±0.07	47.5±1.00	-3.8	-0.3
**$DNA8^{A/E*}$**	52.3±0.61	41.5±1.29	58.6±1.02	-2.7	+1.6
**$DNA9^{A/E*}$**	54.9±0.60	ND[a]	62.0±0.05	ND[a]	+0.5
**$DNA10^{A/E*}$**		ND[a]	62.3±0.05	ND[a]	+0.5
**$DNA11^{A/E*}$**		ND[a]	61.5±0.03	ND[a]	+0.2

[a] ND: not determined; [b] taken from reference[2]

Melting temperatures determined by CD spectroscopy (**Table S6**) were obtained from the negative maxima in the spectral region from 245 to 254 nm and from positive

maxima in the spectral region from 275 to 282 nm. According to the results, the most destabilizing effect have three pentylC modifications included in **DNA5^{APr}** with the T_m decrease of -4.3 °C which is in contrast with T_m measured by UV-vis spectroscopy, where the **DNA5^{APr}** has the lowest destabilization only by -1.5 °C. Similarly to the data from UV-vis, **DNA1^{Ain}**, **DNA6^{APh}** and **DNA2^{AiPr}** with one incorporated indole, phenyl and isopropyl modification, respectively, have higher destabilizing effect (by -3.7 °C, -3.7 °C and -3.4 °C) than the more modified **DNA3^{AiPr}**, **DNA7^{APh}** and **DNA8^{A*}** (-2.6 °C, -2.3 °C, -1.6 °C, respsectively). On the contrary, one incorporated pentylC modification (**DNA4^{APr}**) has relatively low effect on the duplex destabilization with the decrease of T_m only by -1.3 °C.

Table S6 Melting temperature (T_m, [°C]) of non-modified duplexes (DNA) and modified duplexes (DNA^{A*}) determined by CD spectroscopy at two ranges of absorbing wavelenghts. ΔT_m - the difference of T_m between modified DNA^{A*} and native DNA, and ΔT_m/modification - the difference per one modification.

Code	T_m (275–282 nm - CD) [°C]				T_m (245–254 nm - CD) [°C]			
	DNA	DNA^{A*}	ΔT_m	ΔT_m/modification	DNA	DNA^{A*}	ΔT_m	ΔT_m/modification
**DNA1^{AIn}**	53.1	50.8	-2.3	-2.3	51.8	48.1	-3.7	-3.7
**DNA2^{AiPr}**	57.6	54.7	-2.9	-2.9	56.2	52.8	-3.4	-3.4
**DNA3^{AiPr}**	61.9	52.9	-9.0	-3.0	56.8	49.0	-7.8	-2.6
**DNA4^{APr}**	56.1	53.3	-2.8	-2.8	55.3	54.0	-1.3	-1.3
**DNA5^{APr}**	59.4	46.5	-12.9	-4.3	58.0	45.2	-12.8	-4.3
**DNA6^{APh}**	52.2	51.1	-1.1	-1.1	52.8	49.1	-3.7	-3.7
**DNA7^{APh}**	50.2	45.1	-5.1	-1.7	46.5	39.7	-6.8	-2.3
**DNA8^{A*}**	53.6	46.7	-6.9	-1.7	51.3	45.1	-6.2	-1.6
**DNA9^{A*}**		36.3	-19.3	-1.3		22.0	-31.4	-2.0
**DNA10^{A*}**	55.6	36.8	-18.8	-1.3	53.4	31.8	-21.6	-1.4
**DNA11^{A*}**		ND[a]	ND[a]	ND[a]		ND[a]	ND[a]	ND[a]

[a] ND: not determined

2.5 Dynamic Light Scattering

Table S7 presents results acquired by dynamic light scattering (DLS) measurements. Solution of ONs in water were concentrated to 0.5 mg/mL and measured at 25°C.

Table S7 Results of dynamic light scattering (DLS) measurements of hyper-modified ONs ($ON9^{A}*$, $ON10^{A}*$), non-modified ONs of the same sequence (ON9, ON0), and blank (UPLC-grade H_2O). Hydrodynamic diameter (Z-average; [d.nm]), polydispersity index (PDI), and derived count rate [kcps] are shown. Results were acquired as triplicates.

Code	Z-Average [d.nm]	PDI	Derived count rate [kcps]
**Blank**	0	0.03±0.045	242±59.6
**ON9**	340±3.5	0.57±0.136	297±40.2
**ON10**	481±104.0	0.55±0.110	568±51.8
**ON9A***	561±30.3	0.60±0.048	2555±107.1
**ON10A***	349±38.6	0.53±0.053	6139±278.7

3 Copies of Chromatograms, Spectra and Graphs

3.1 HPLC Chromatograms of Synthesized Oligonucleotides

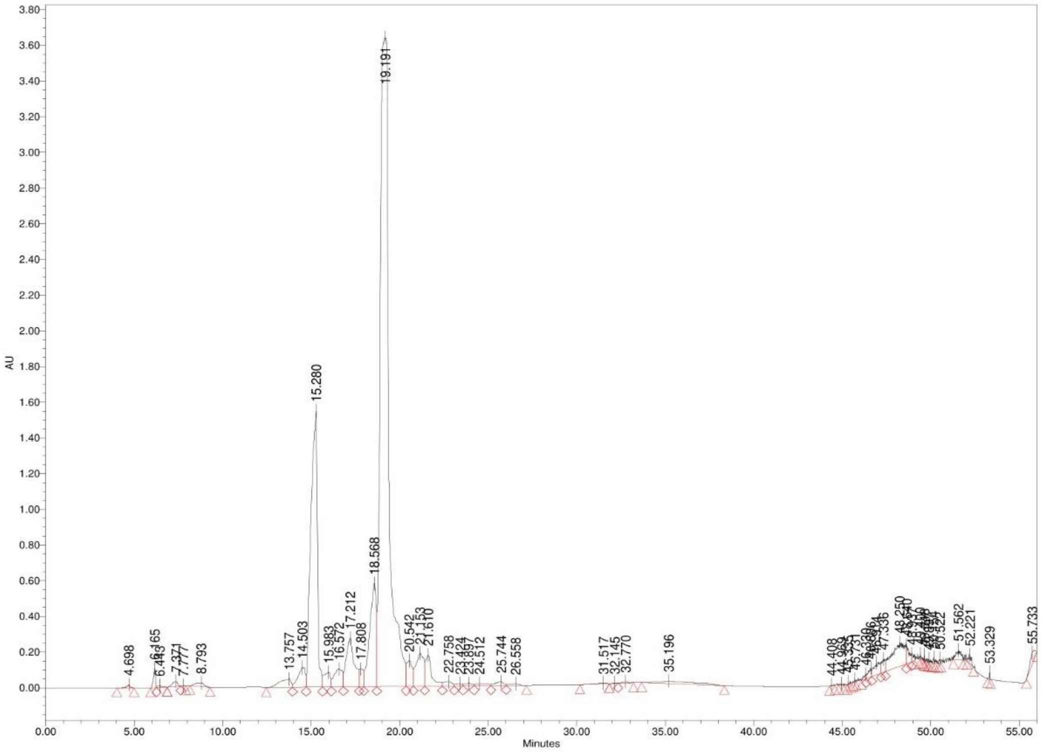

Figure S1 Chromatogram from HPLC purification of ON1^{AIn}. Peak colected at 19.191 min.

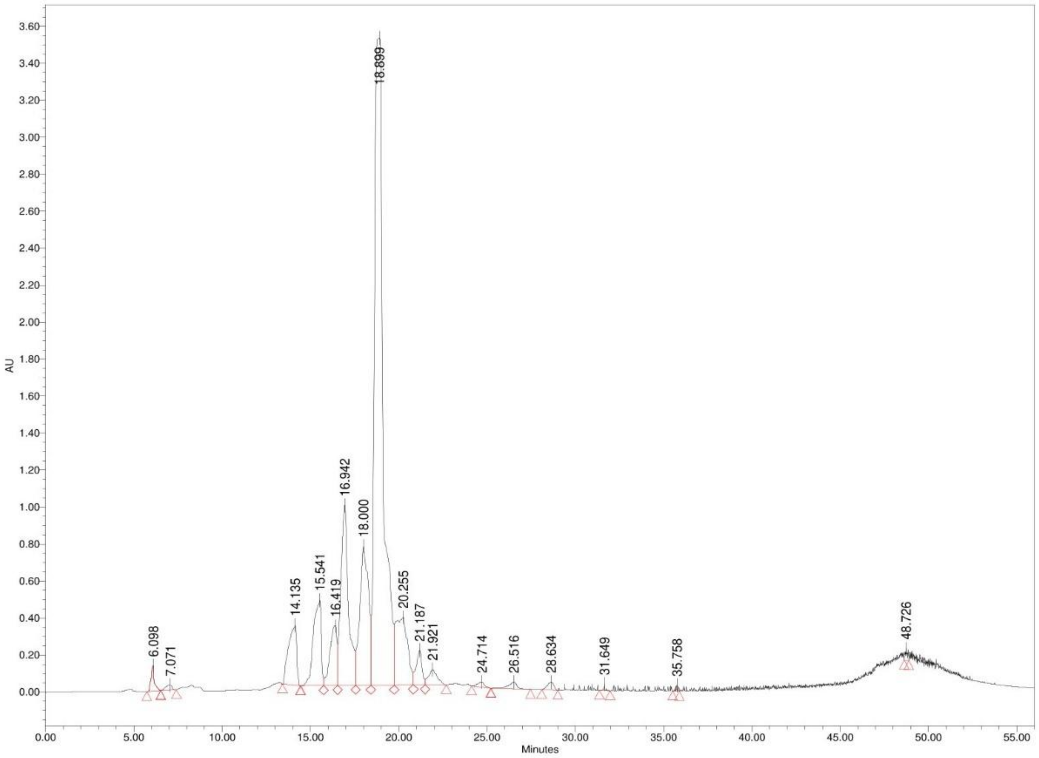

Figure S2 Chromatogram from HPLC purification of ON2^{AiPr}. Time of peak collected: 18.899 min.

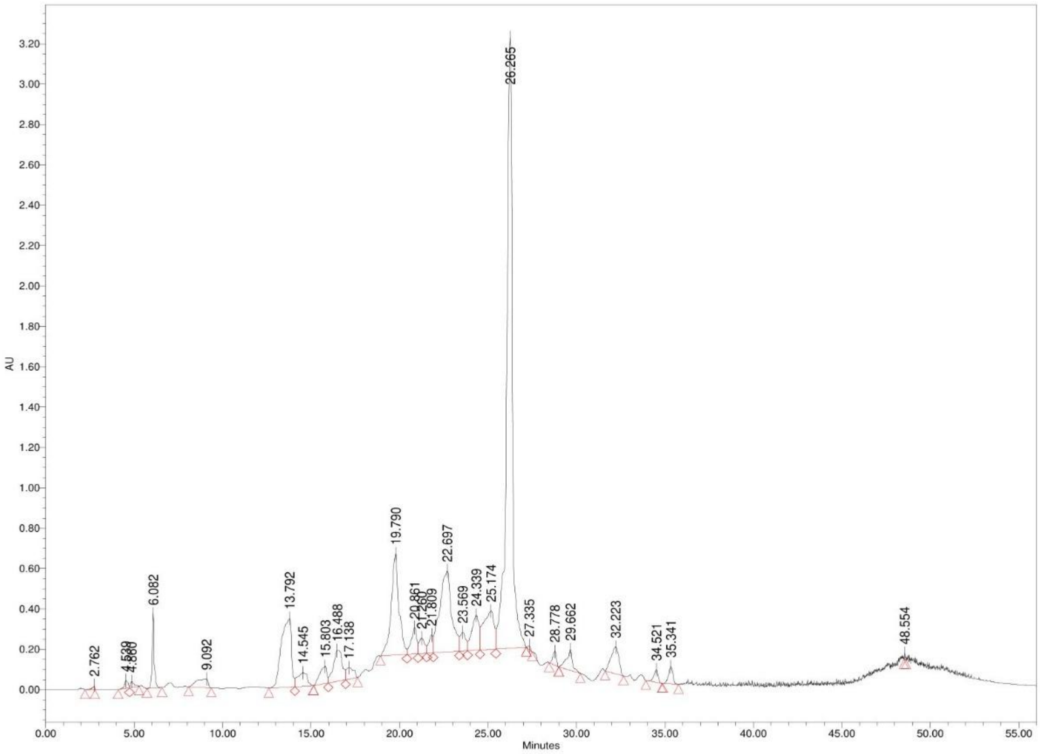

Figure S3 Chromatogram from HPLC purification of ON3^{AiPr}. Time of peak collected: 26.265 min.

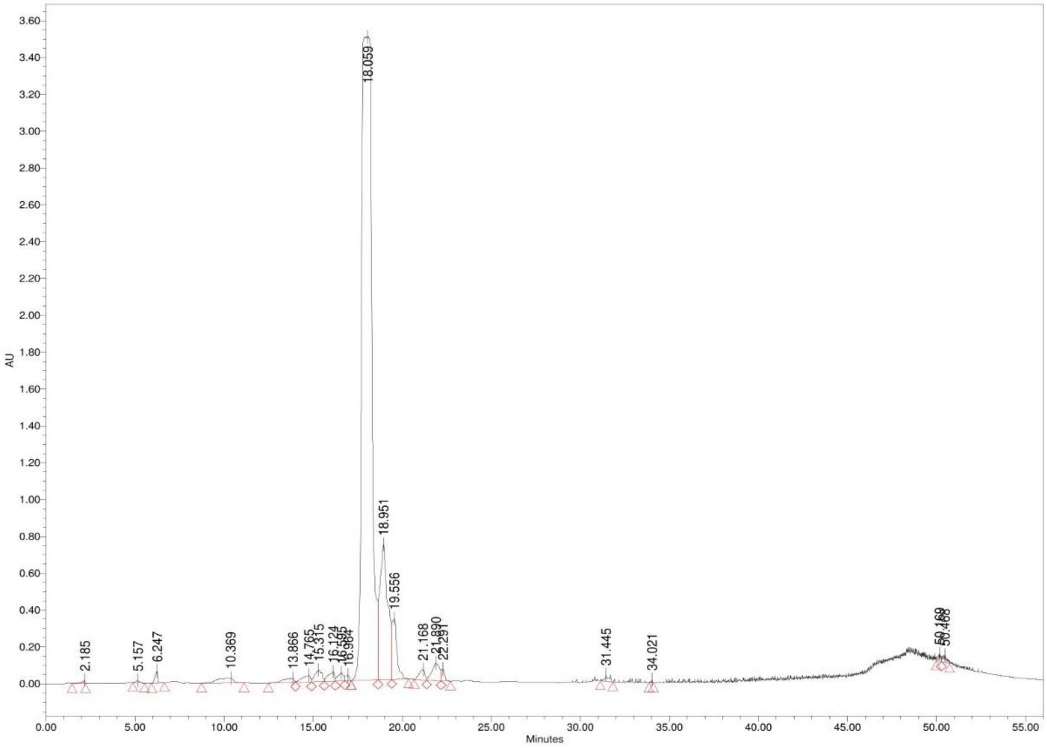

Figure S4 Chromatogram from HPLC purification of ON4^{AiPr}. Time of peak collected: 18.059 min.

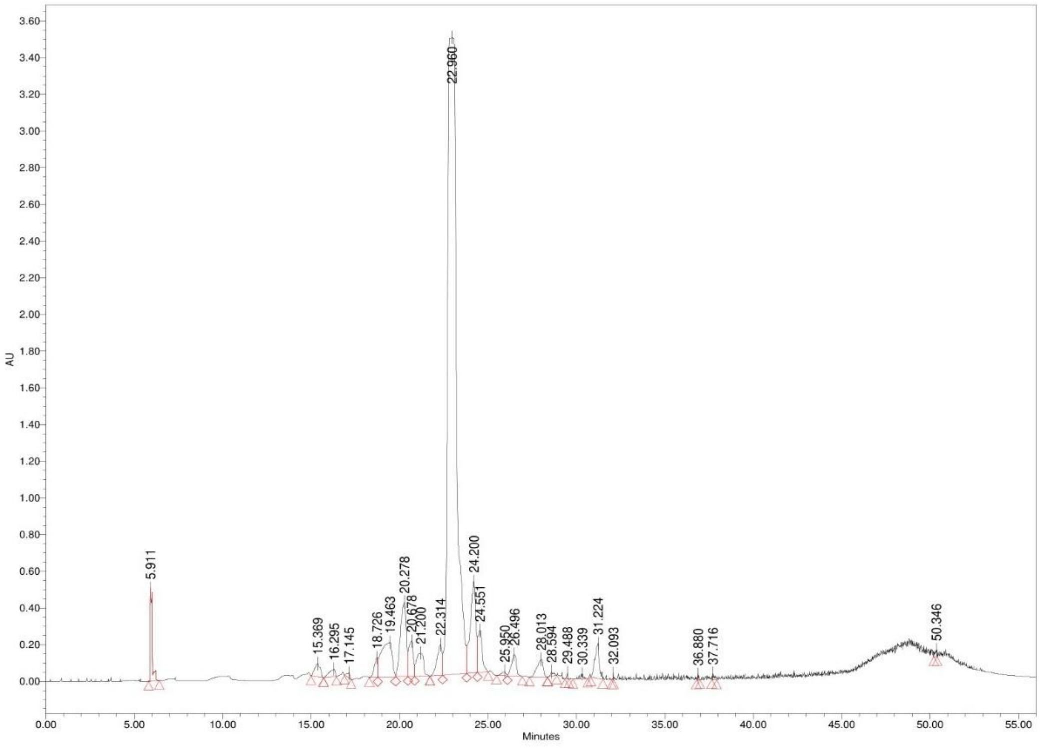

Figure S5 Chromatogram from HPLC purification of ON5^{AiPr}. Time of peak collected: 22.960 min.

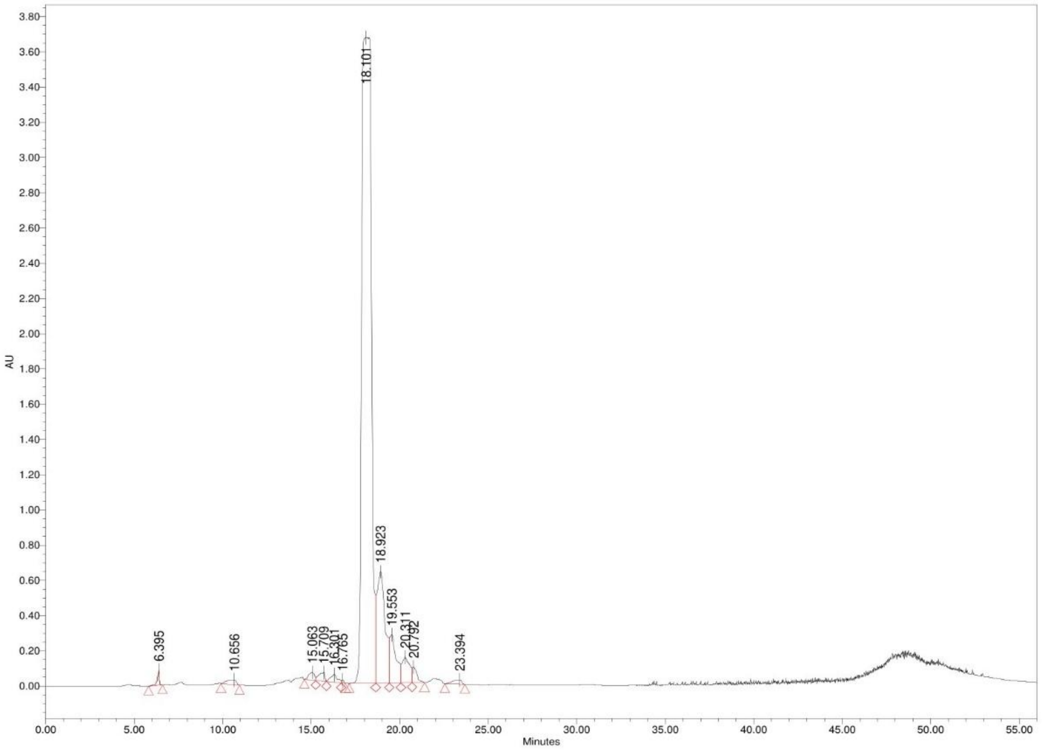

Figure S6 Chromatogram from HPLC purification of ON6^{APh}. Time of peak collected: 18.101 min.

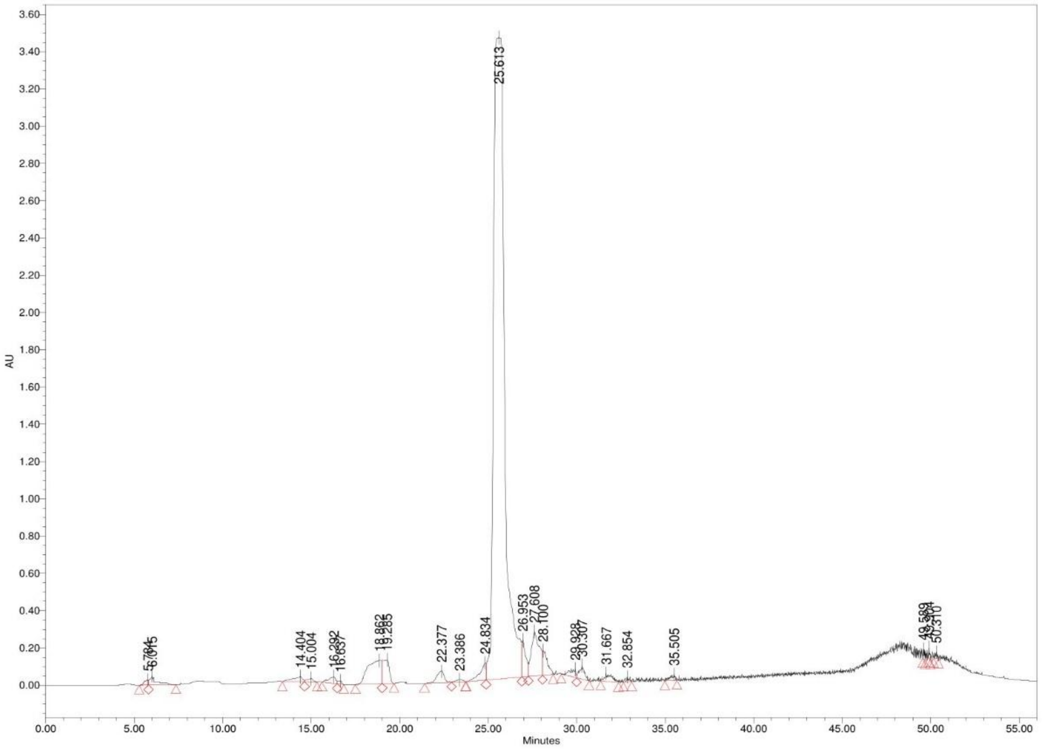

Figure S7 Chromatogram from HPLC purification of ON7^{APh}. Time of peak collected: 25.613 min.

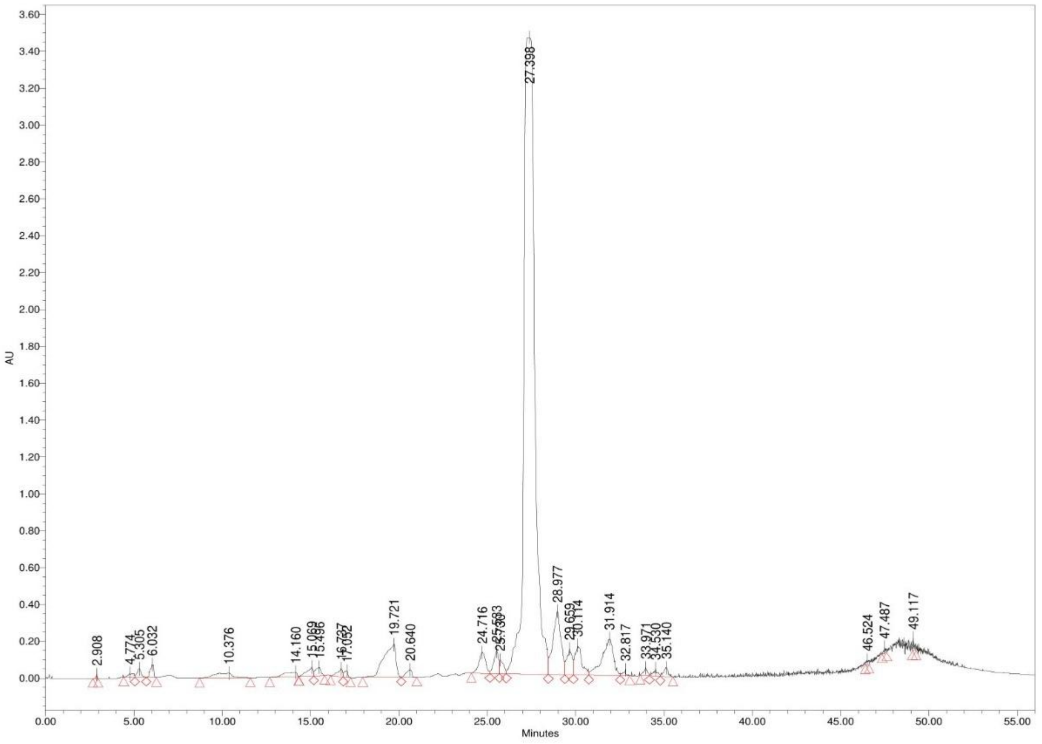

Figure S8 Chromatogram from HPLC purification of ON8^{A*}. Time of peak collected: 27.398 min.

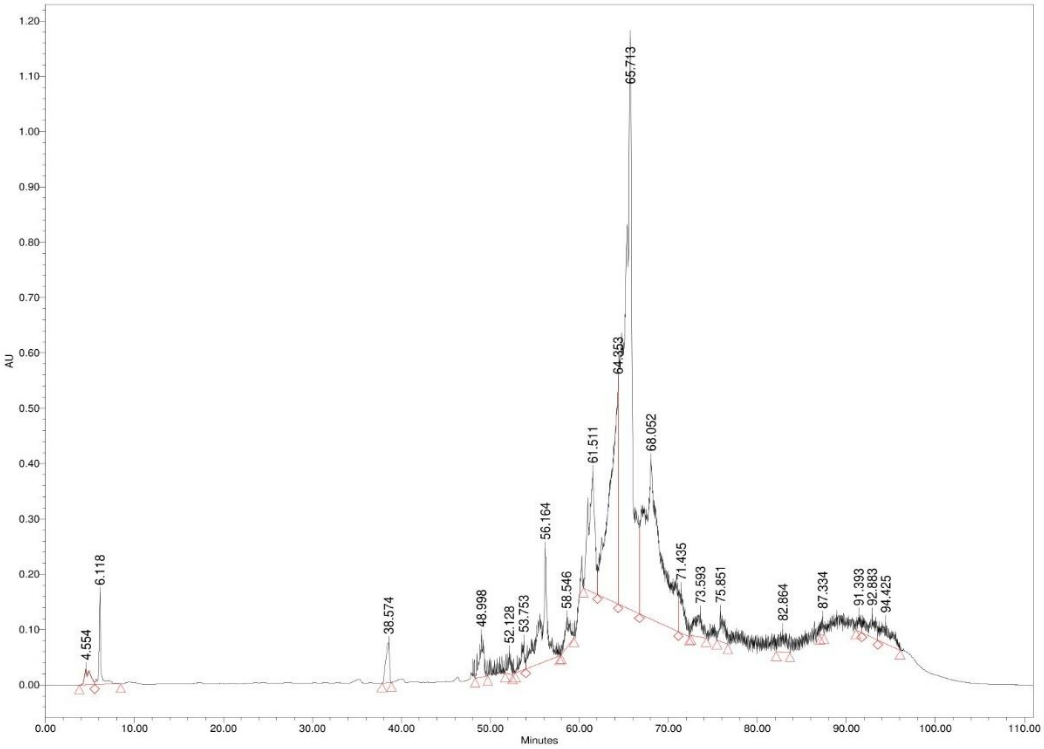

Figure S9 Chromatogram from HPLC purification of ON9^{A*}. Time of peak collected: 65.713 min.

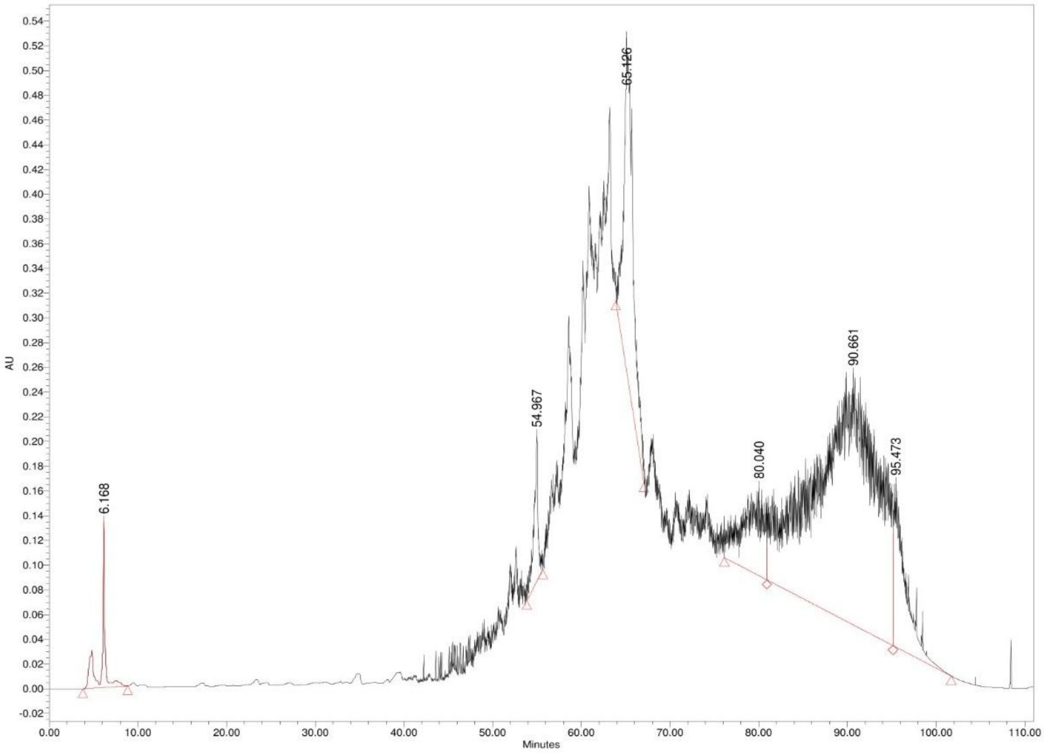

Figure S10 Chromatogram from HPLC purification of ON10^{A*}. Time of peak collected: 65.126 min.

3.2 Absorbance Chromatograms of Purified Oligonucleotides

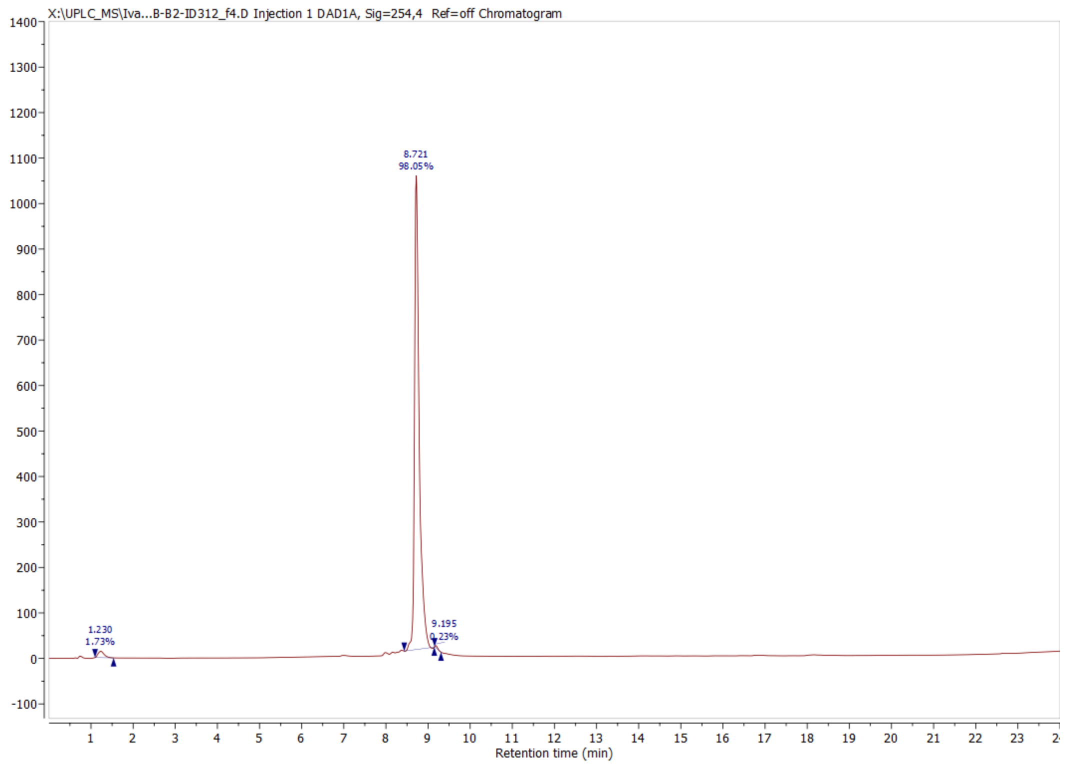

Figure S11 Absorbance chromatogram at 254 nm of ON1^{AIn}.

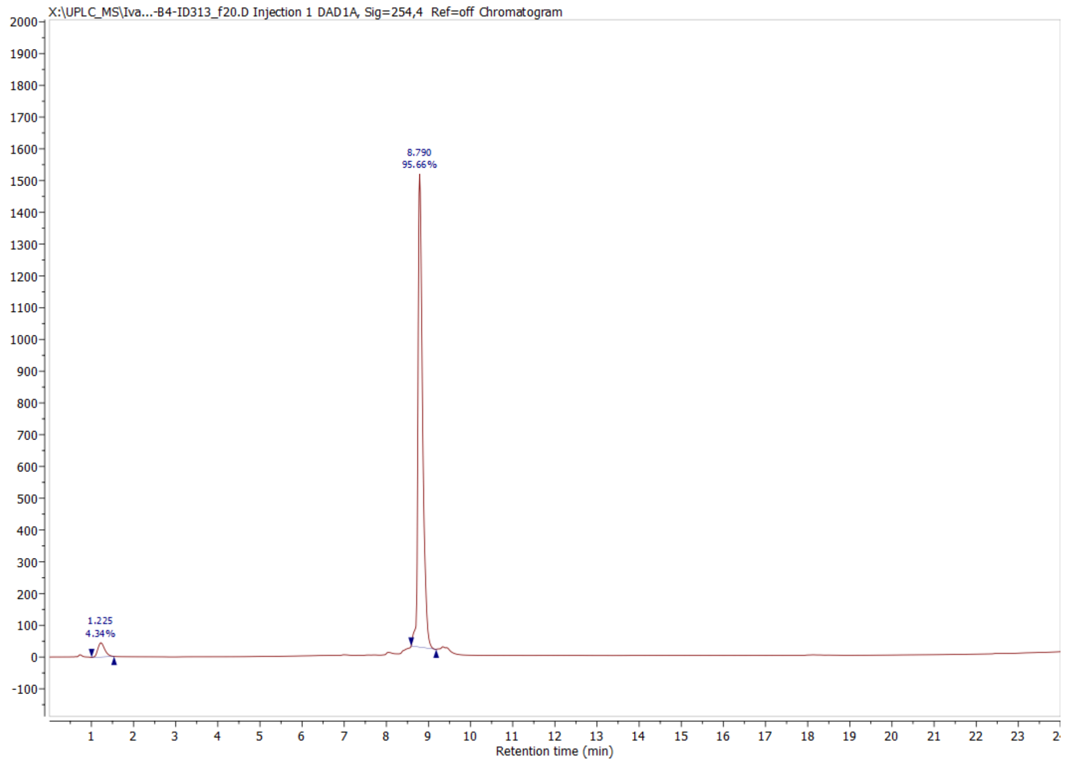

Figure S12 Absorbance chromatogram at 254 nm of ON2^{AiPr}.

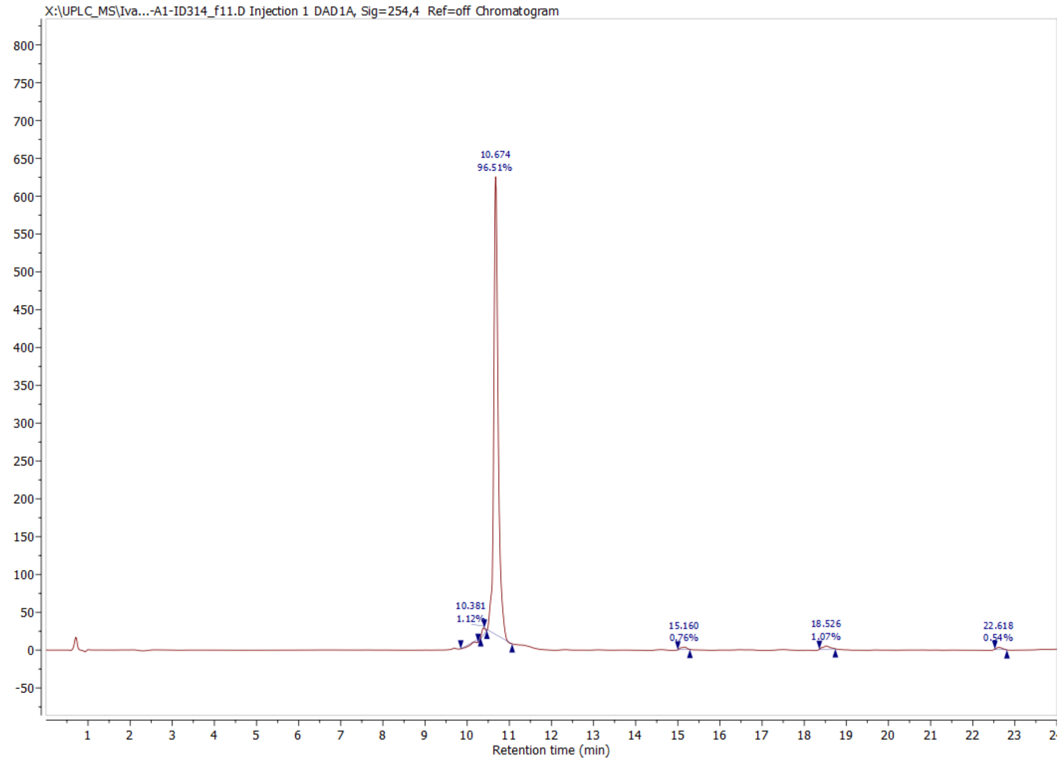

Figure S13 Absorbance chromatogram at 254 nm of ON3^{AiPr}.

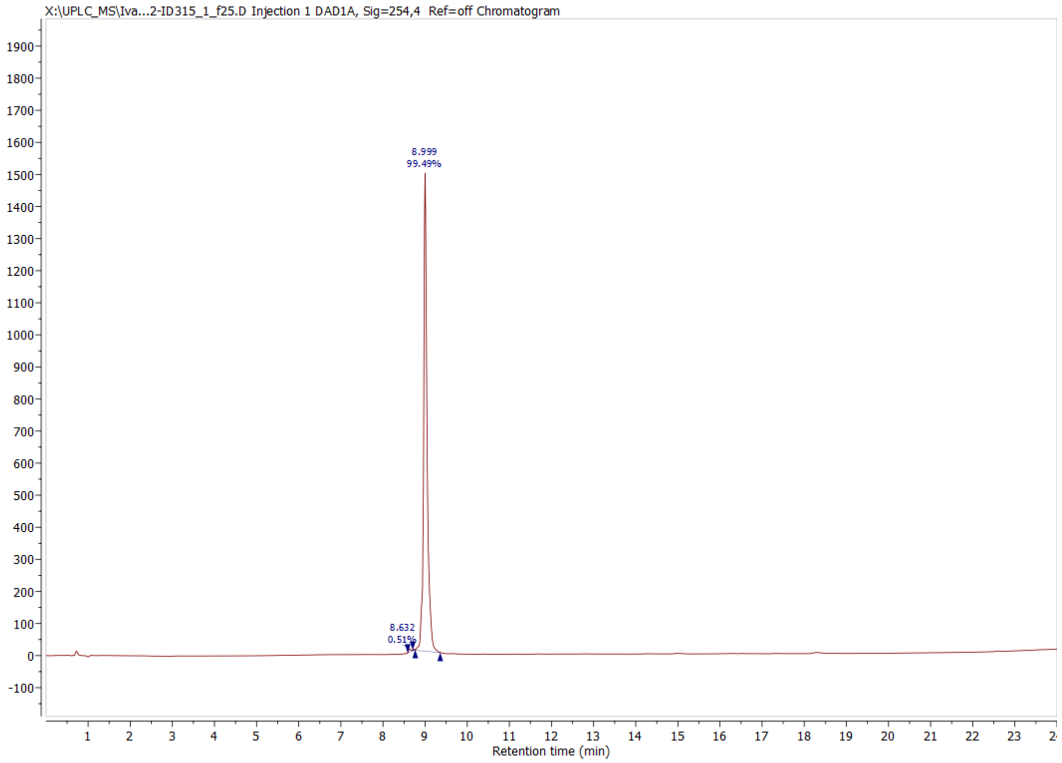

Figure S14 Absorbance chromatogram at 254 nm of ON4^{APr}.

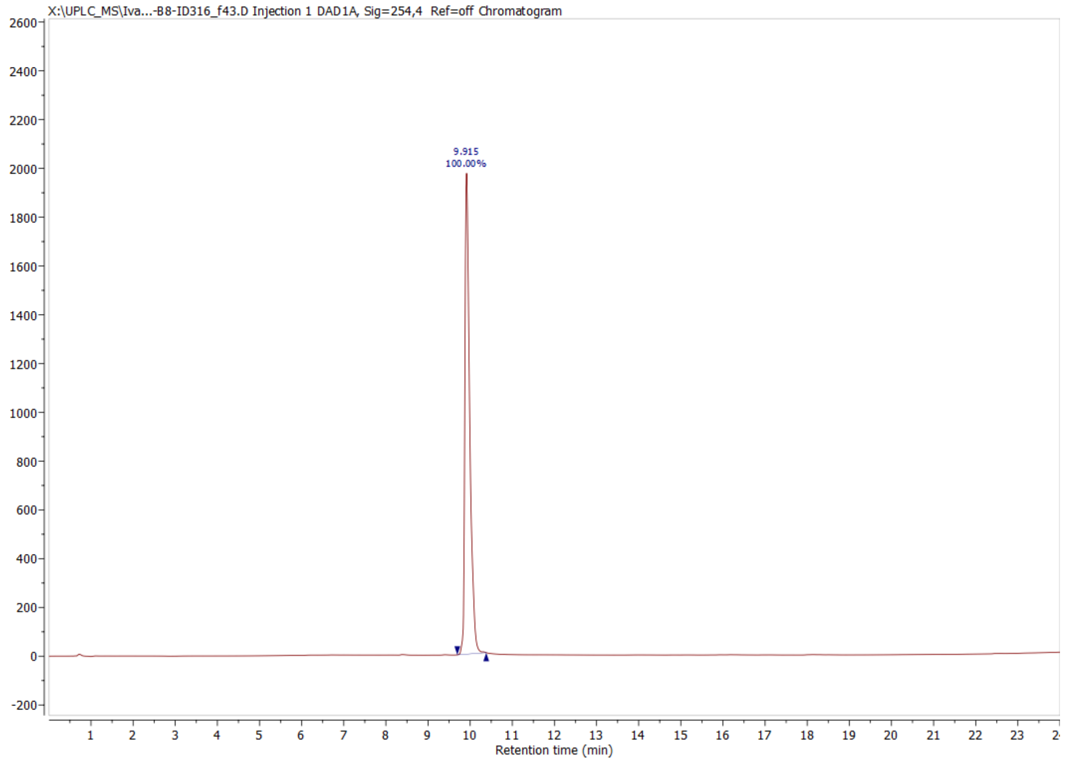

Figure S15 Absorbance chromatogram at 254 nm of ON5^{APr}.

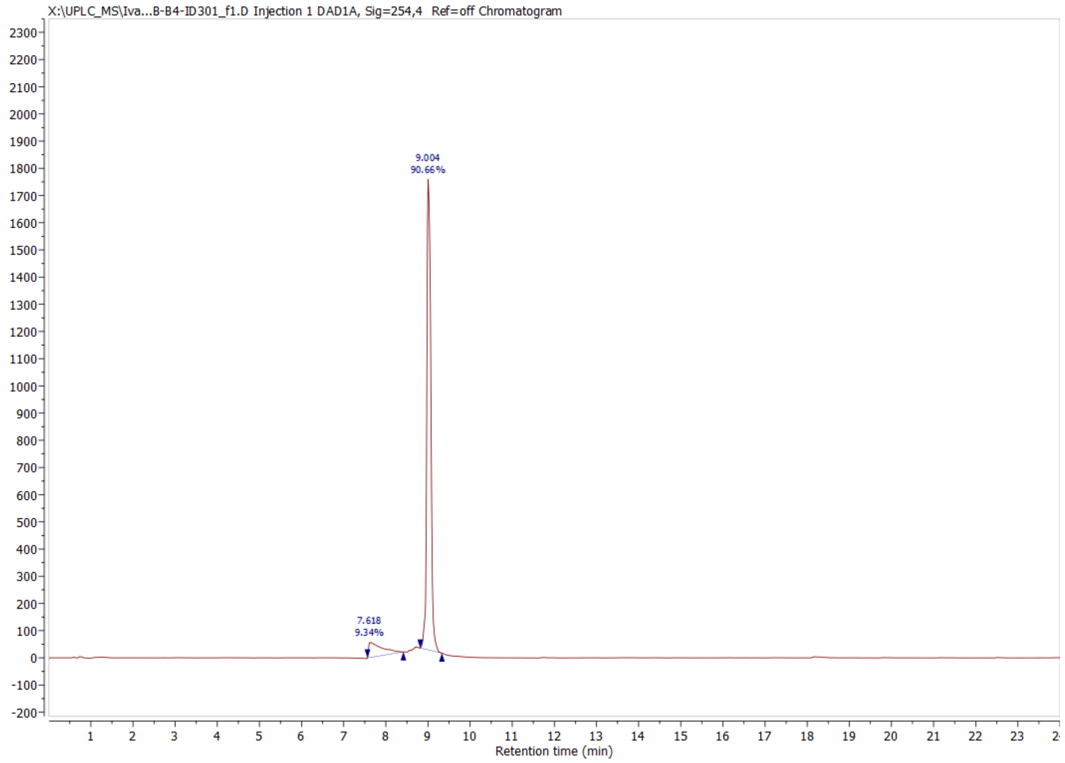

Figure S16 Absorbance chromatogram at 254 nm of ON6^{APh}.

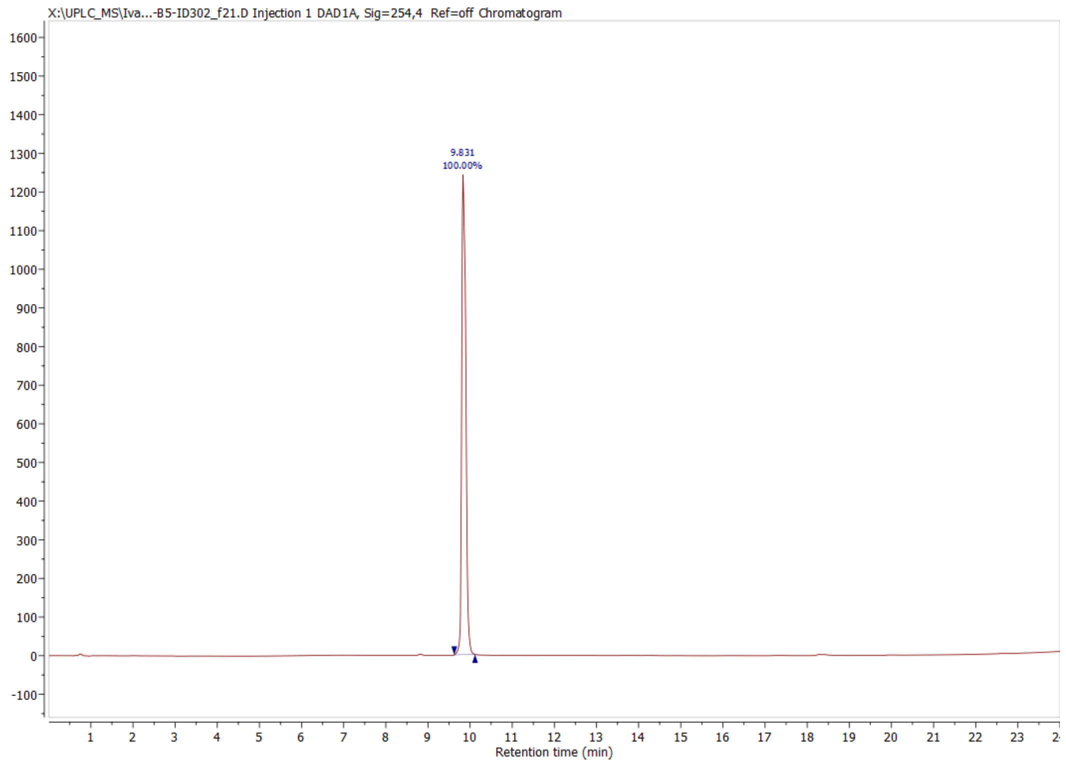

Figure S17 Absorbance chromatogram at 254 nm of ON7^{APh}.

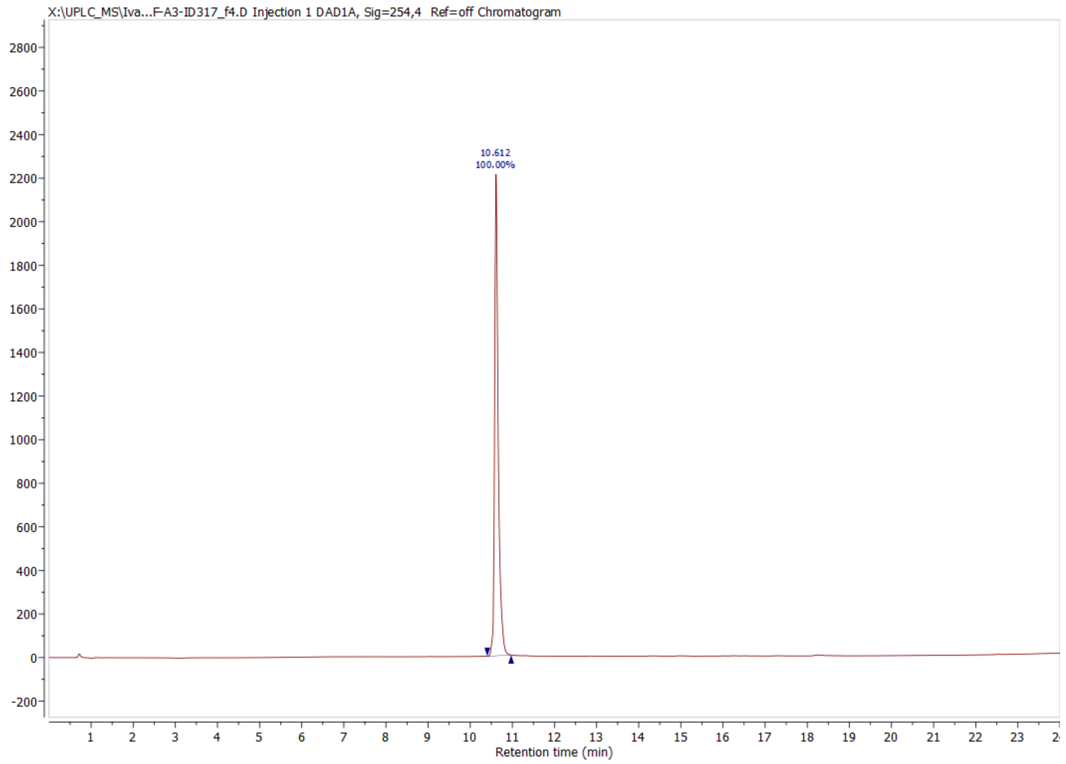

Figure S18 Absorbance chromatogram at 254 nm of ON8^{A*}.

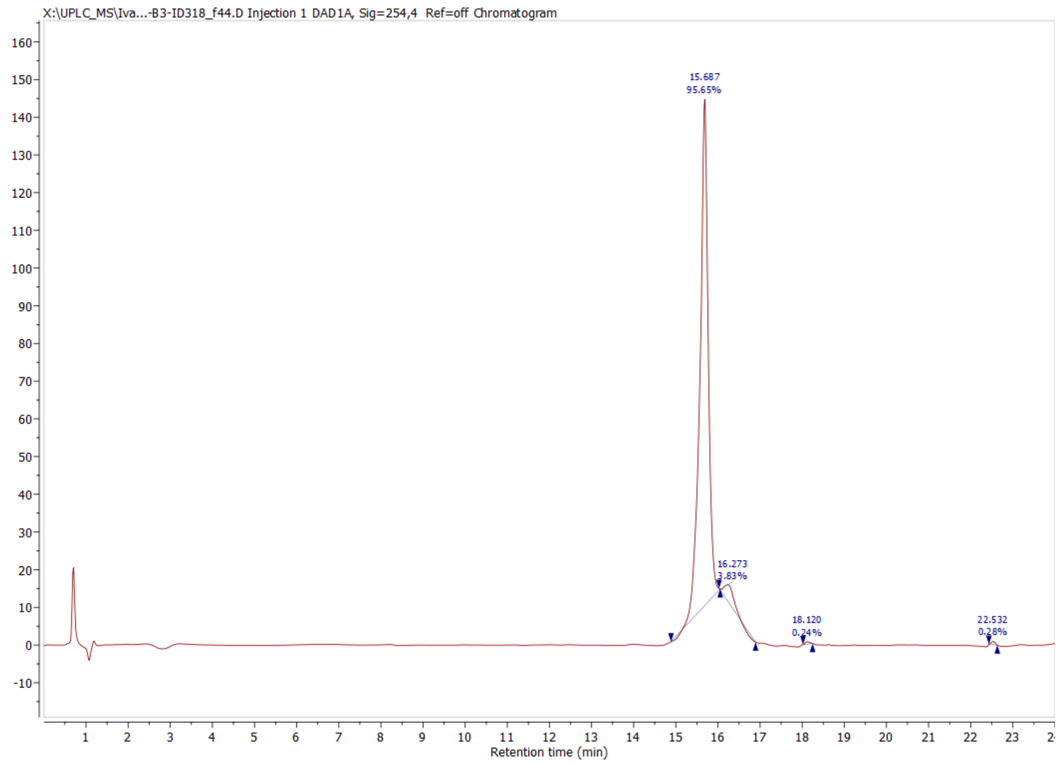

Figure S19 Absorbance chromatogram at 254 nm of ON9[A*].

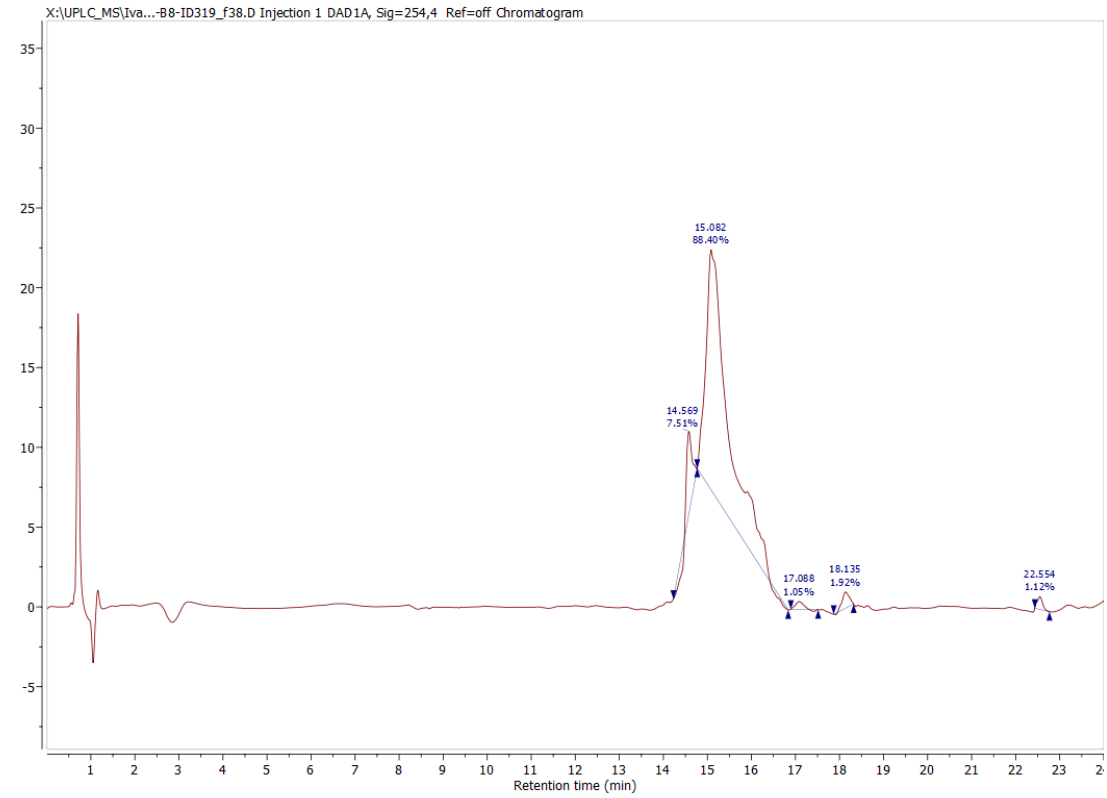

Figure S20 Absorbance chromatogram at 254 nm of ON10[A*].

3.3 ESI Spectra of Oligonucletotides

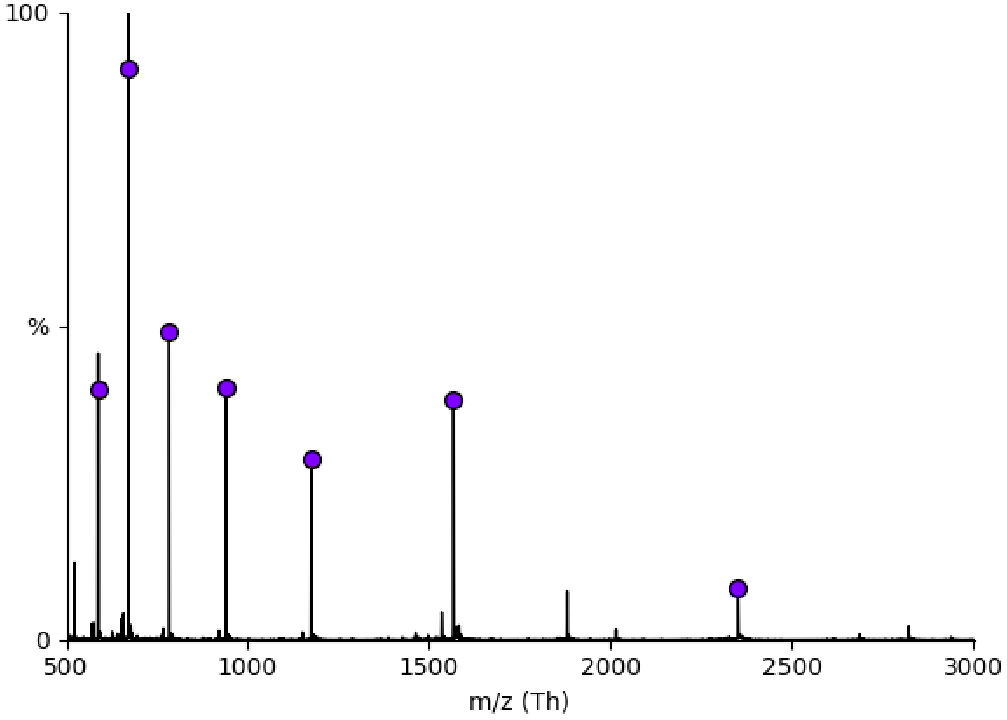

Figure S21 Raw MS spectrum of ON1^{AIn}.

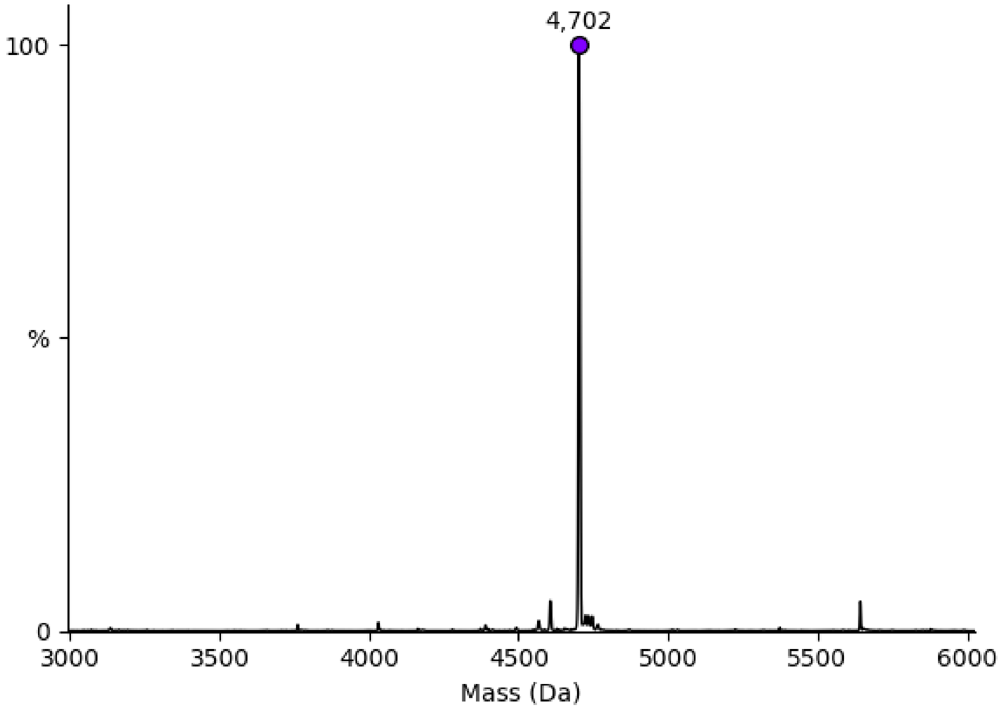

Figure S22 Deconvoluted MS spectrum of ON1^{AIn}: calculated: 4703.20 Da; found: 4702 Da, Δ = 1.20 Da.

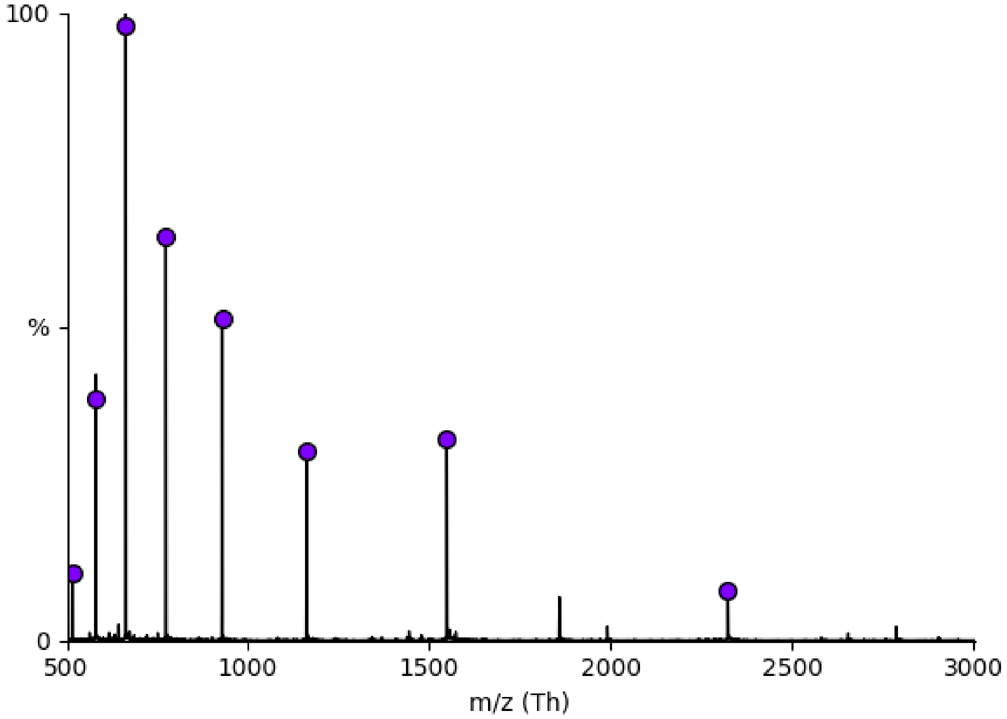

Figure S23 Raw MS spectrum of ON2^{AiPr}.

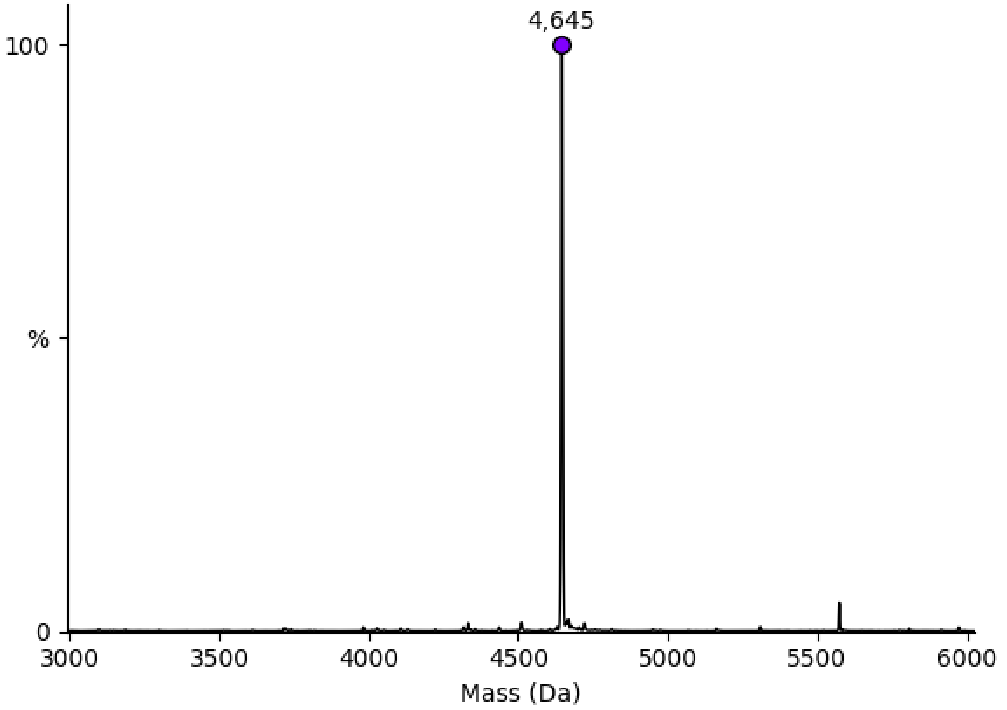

Figure S24 Deconvoluted MS spectrum of ON2^{AiPr}: calculated: 4646.15 Da; found: 4645 Da, Δ = 1.15 Da.

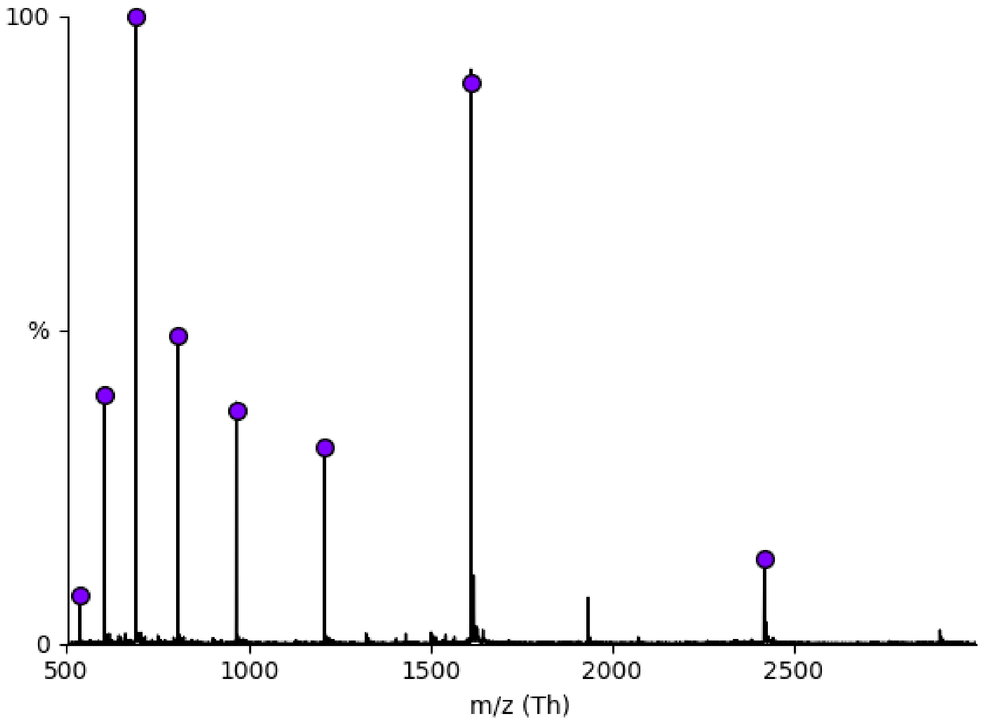

Figure S25 Raw MS spectrum of $ON3^{AiPr}$.

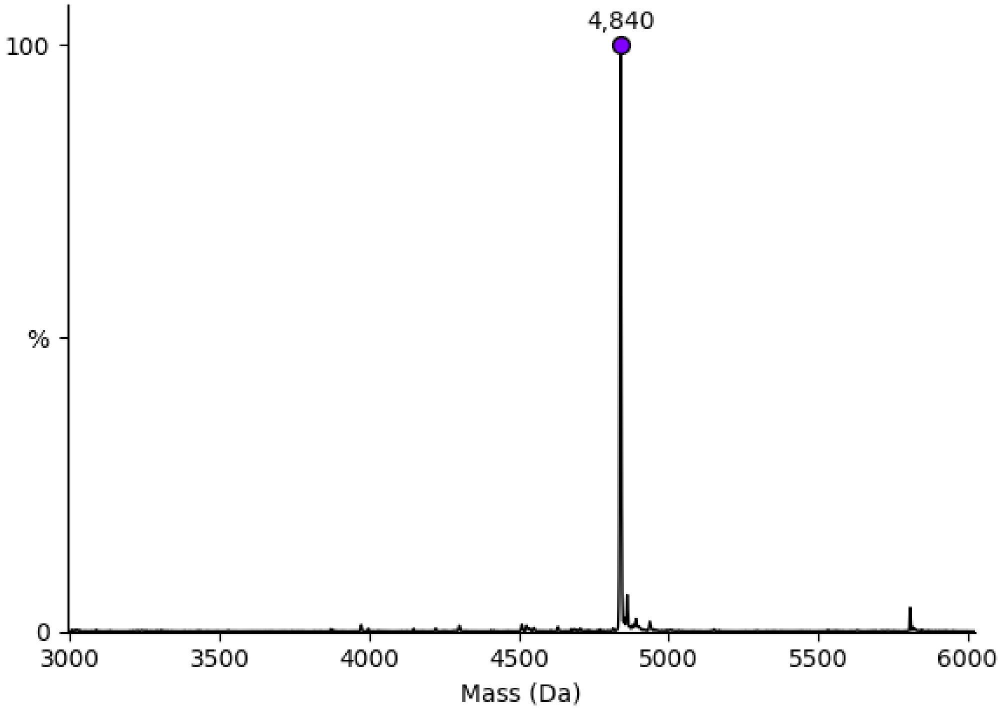

Figure S26 Deconvoluted MS spectrum of $ON3^{AiPr}$: calculated: 4840.54 Da; found: 4840 Da, $\Delta = 0.54$ Da.

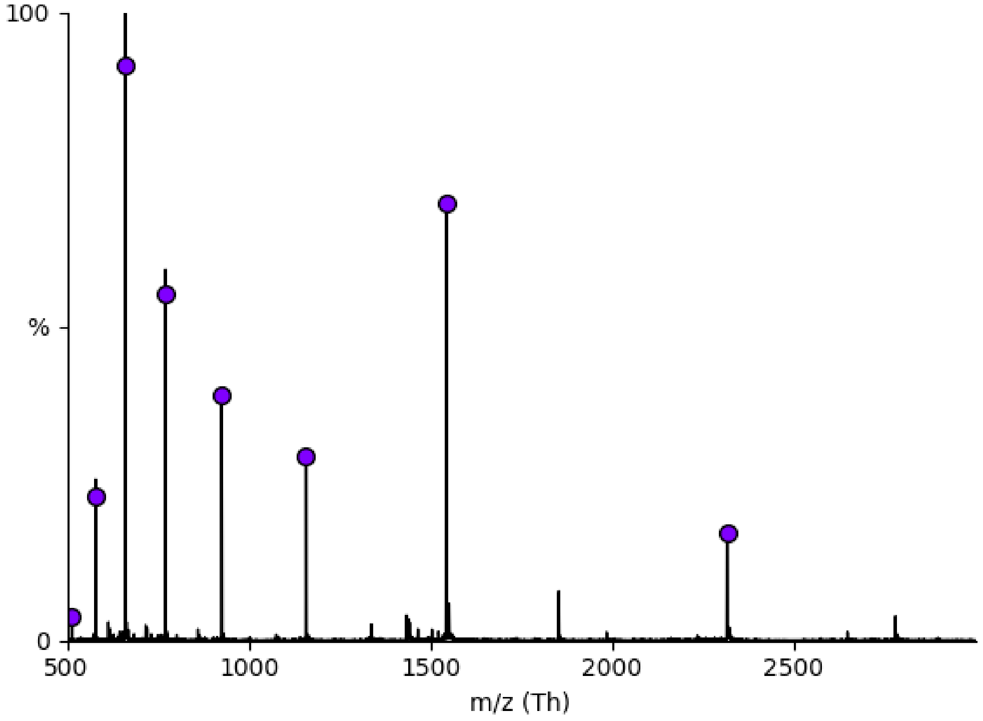

Figure S27 Raw MS spectrum of ON4^{APr}.

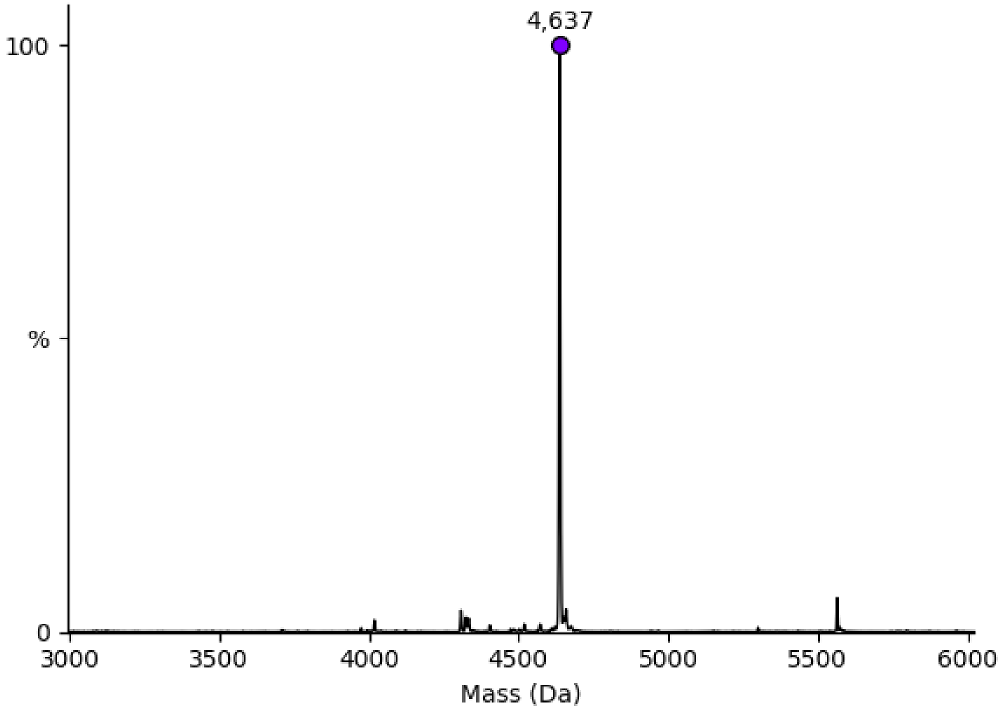

Figure S28 Deconvoluted MS spectrum of ON4^{APr}: calculated: 4638.14 Da; found: 4637 Da, Δ = 1.14 Da.

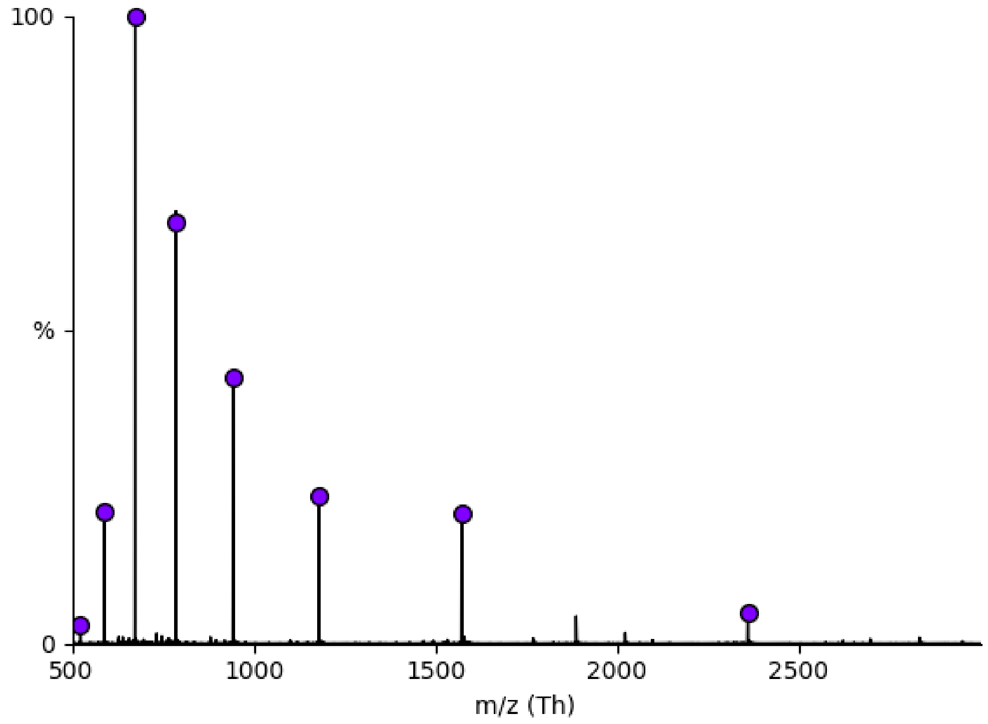

Figure S29 Raw MS spectrum of ON5^{APr}.

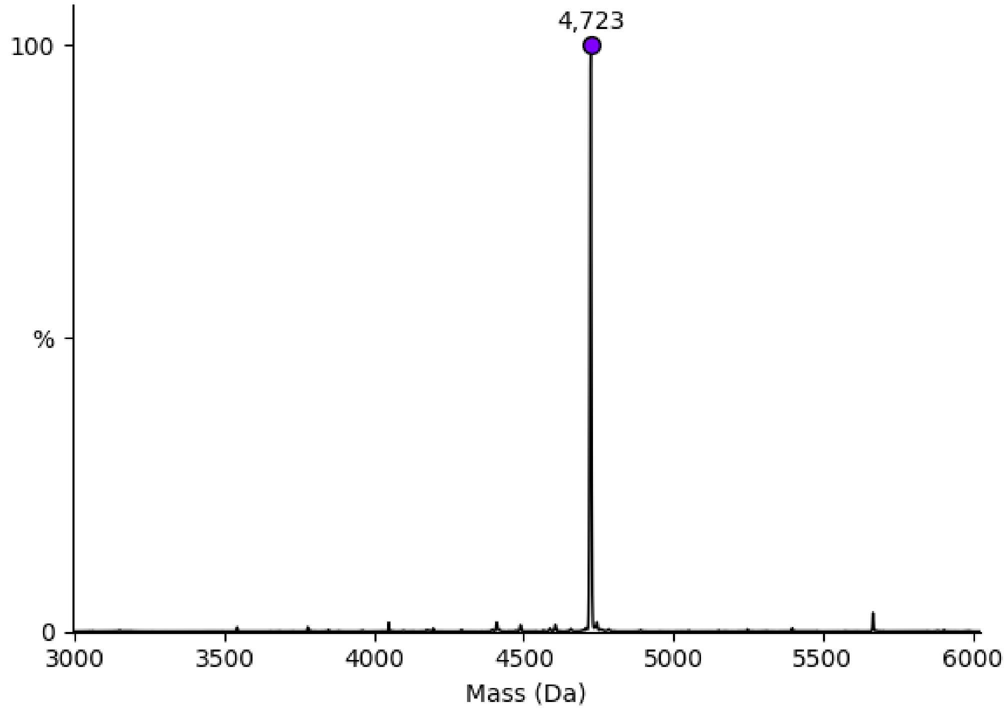

Figure S30 Deconvoluted MS spectrum of ON5^{APr}: calculated: 4723.41 Da; found: 4723 Da, Δ = 0.41 Da.

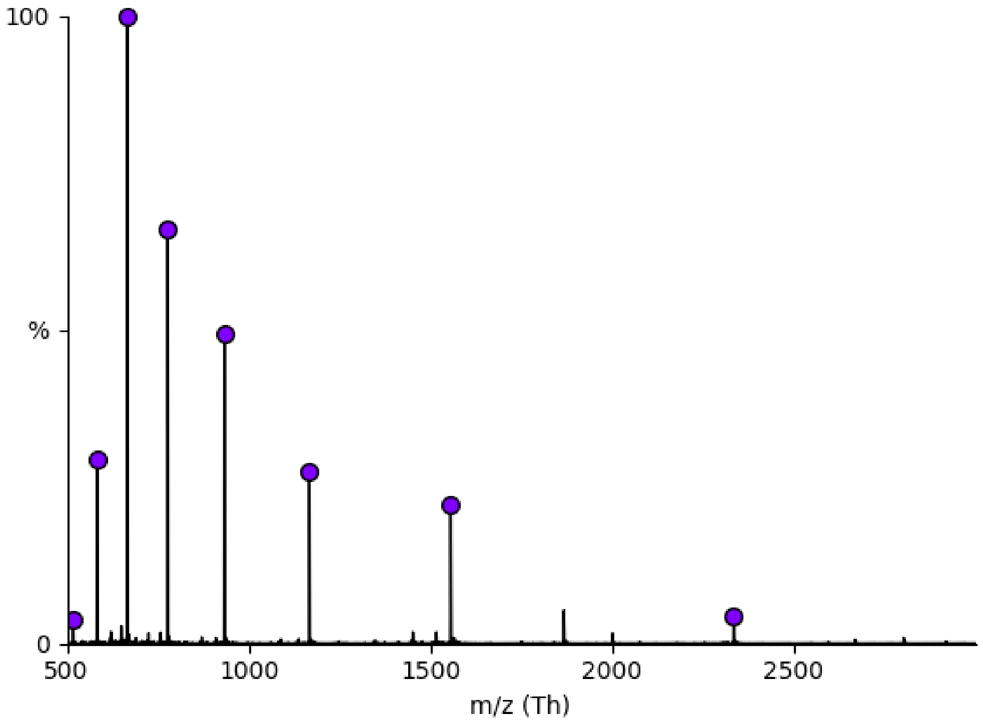

Figure S31 Raw MS spectrum of ON6^{APh}.

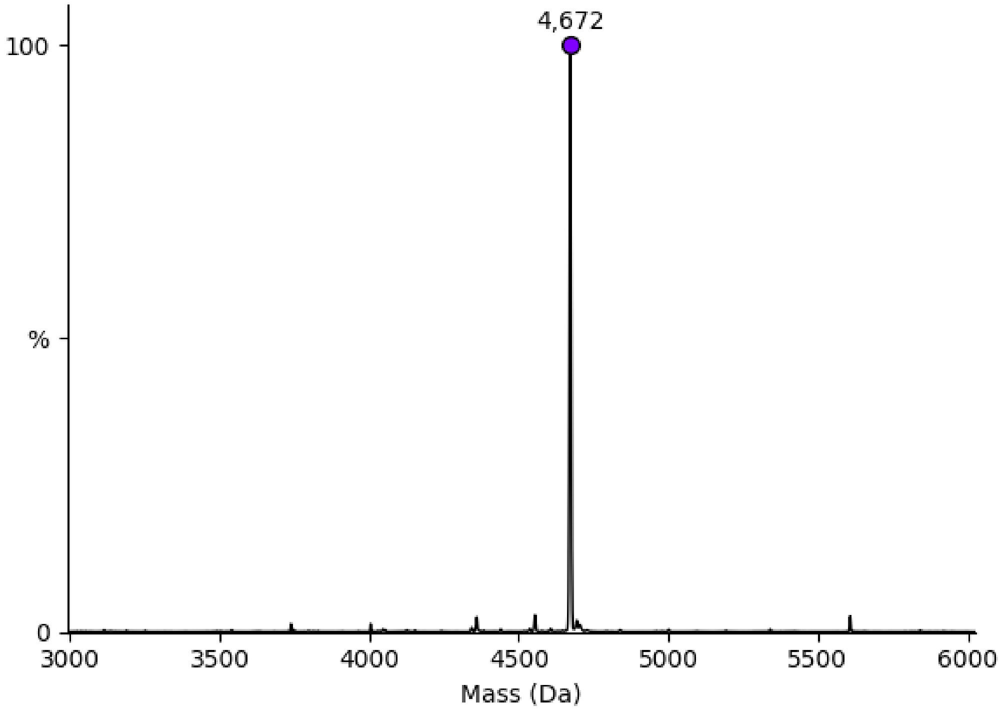

Figure S32 Deconvoluted MS spectrum of ON6^{APh}: calculated: 4673.23 Da; found: 4672 Da, Δ = 1.23 Da.

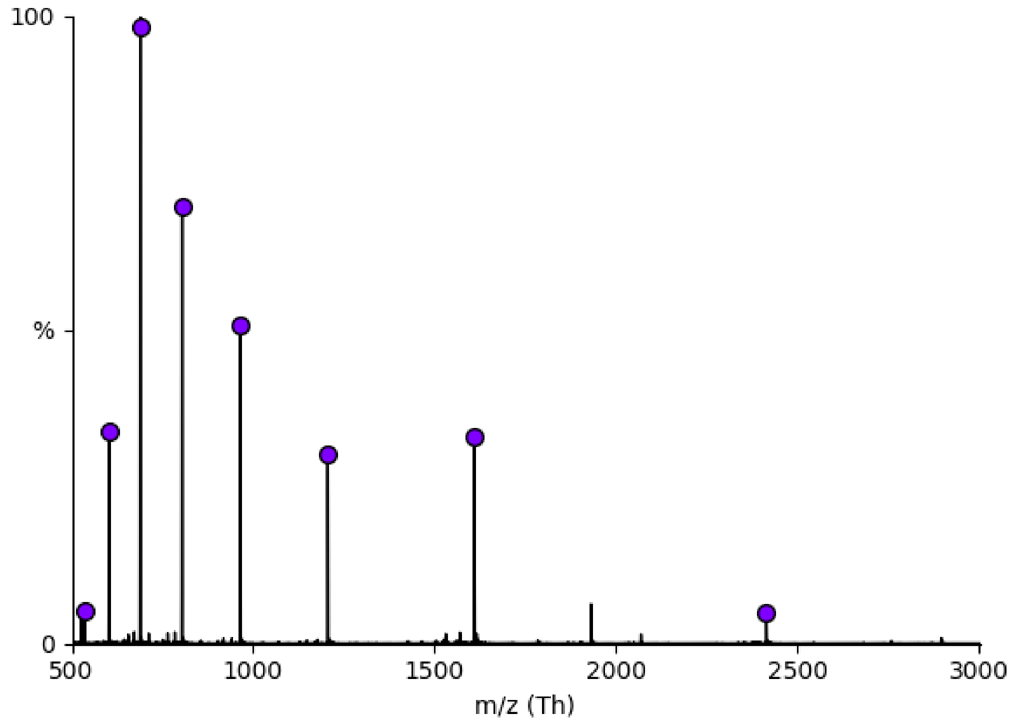

Figure S33 Raw MS spectrum of ON7^{APh}.

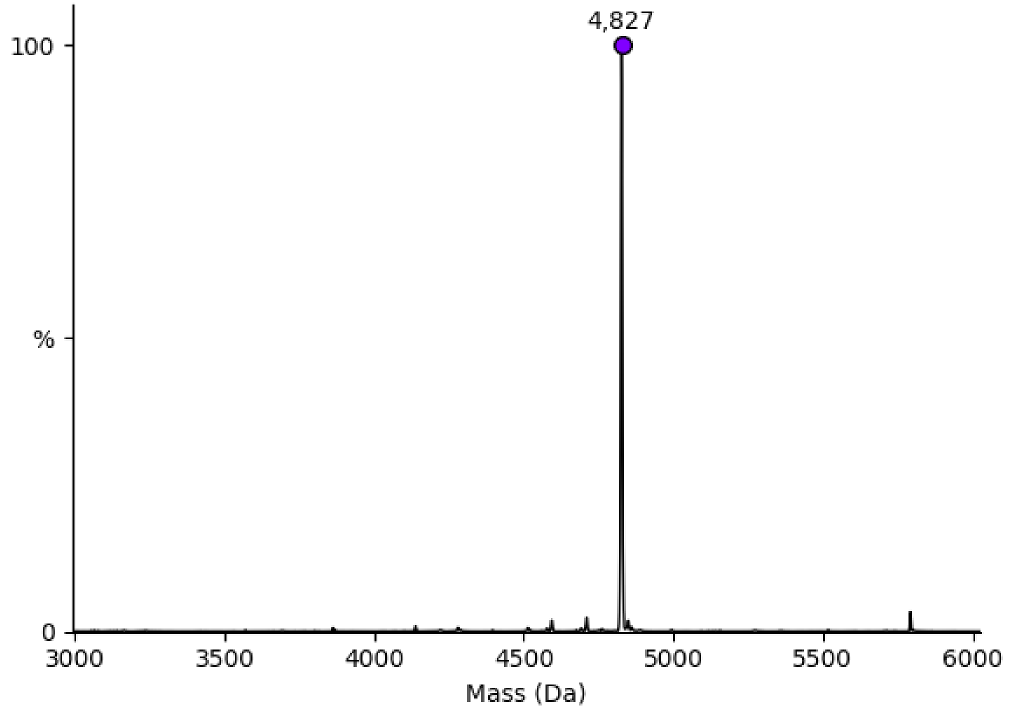

Figure S34 Deconvoluted MS spectrum of ON7^{APh}: calculated: 4828.38 Da; found: 4827 Da, Δ = 0.38 Da.

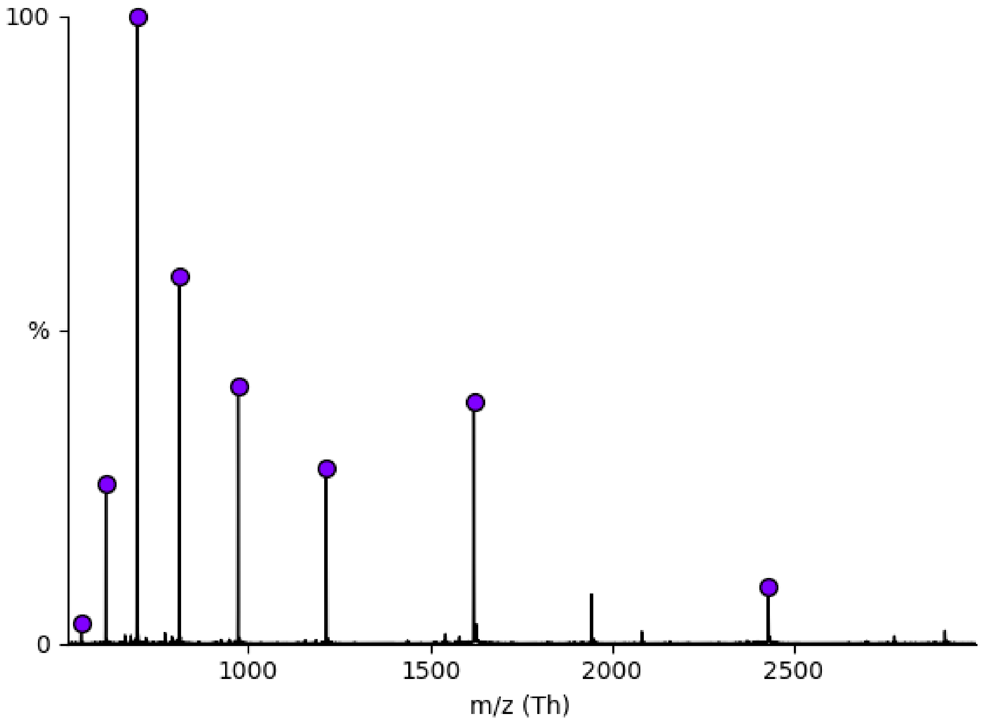

Figure S35 Raw MS spectrum of ON8^{A*}.

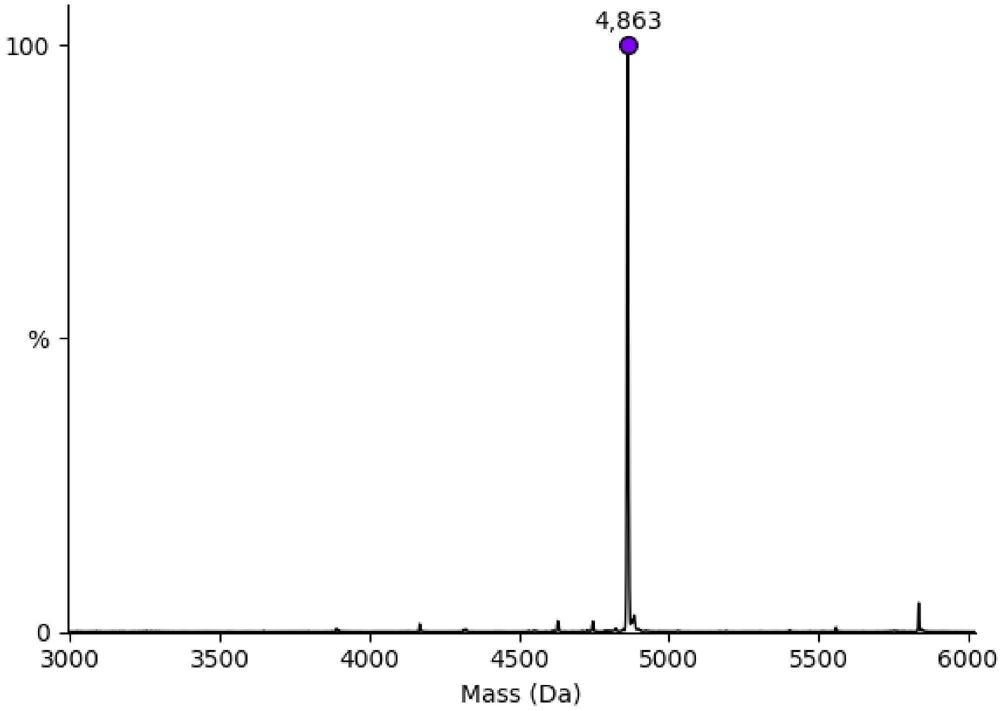

Figure S36 Deconvoluted MS spectrum of ON8^{A*}: calculated: 4863.52 Da; found: 4863 Da, $\Delta = 0.52$ Da.

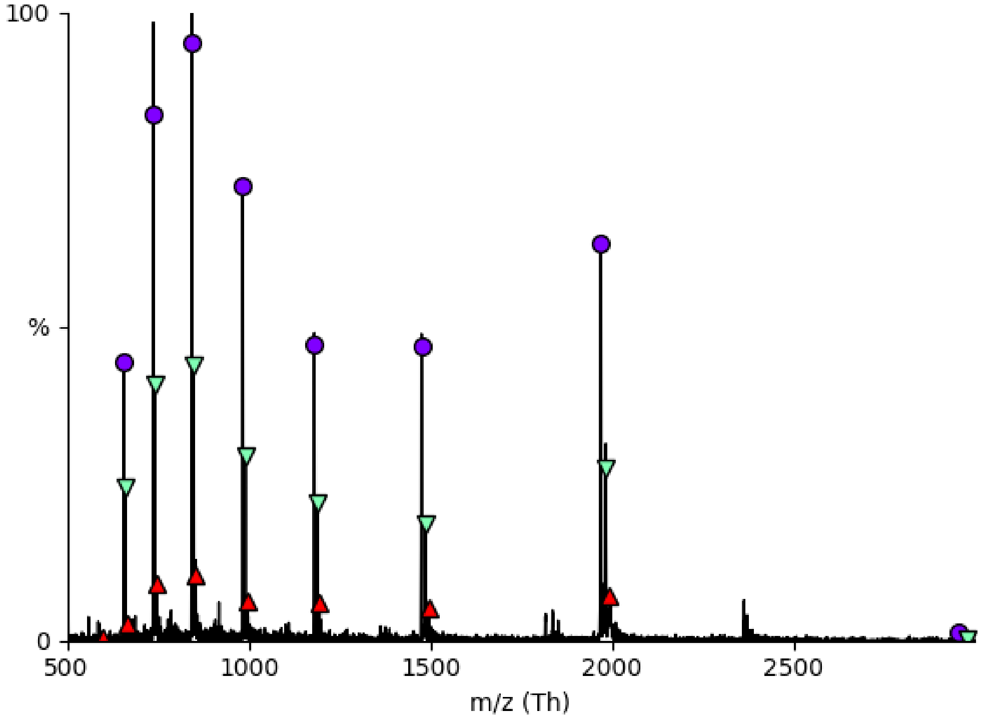

Figure S37 Raw MS spectrum of ON9^{A*}.

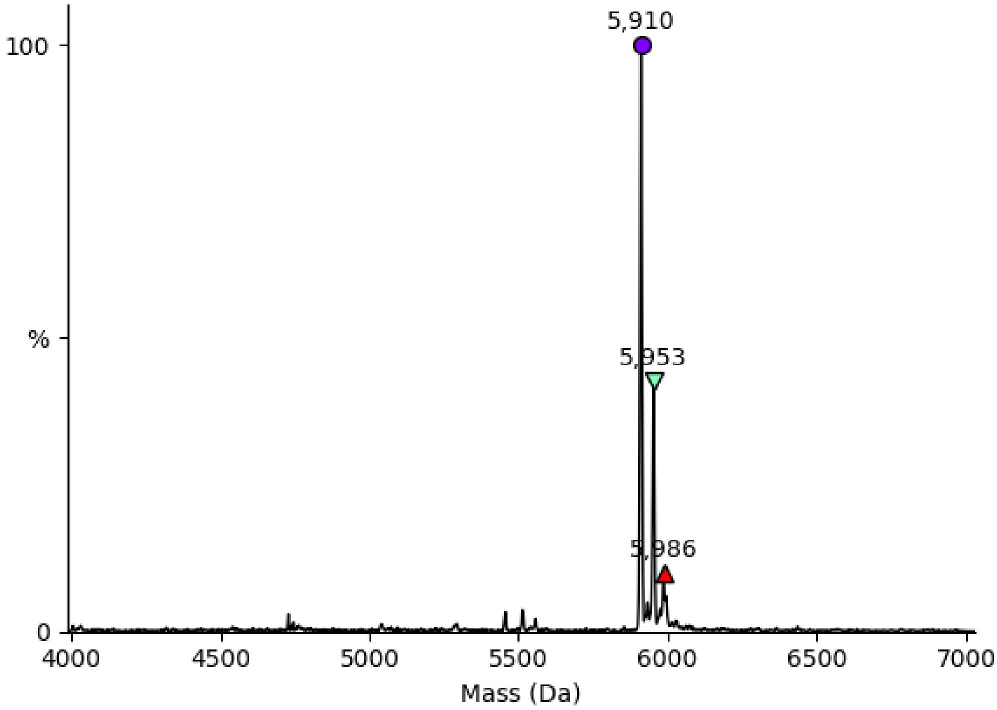

Figure S38 Deconvoluted MS spectrum of ON9^{A*}: calculated: 5912.23 Da; found: 5910 Da, Δ = 2.23 Da.

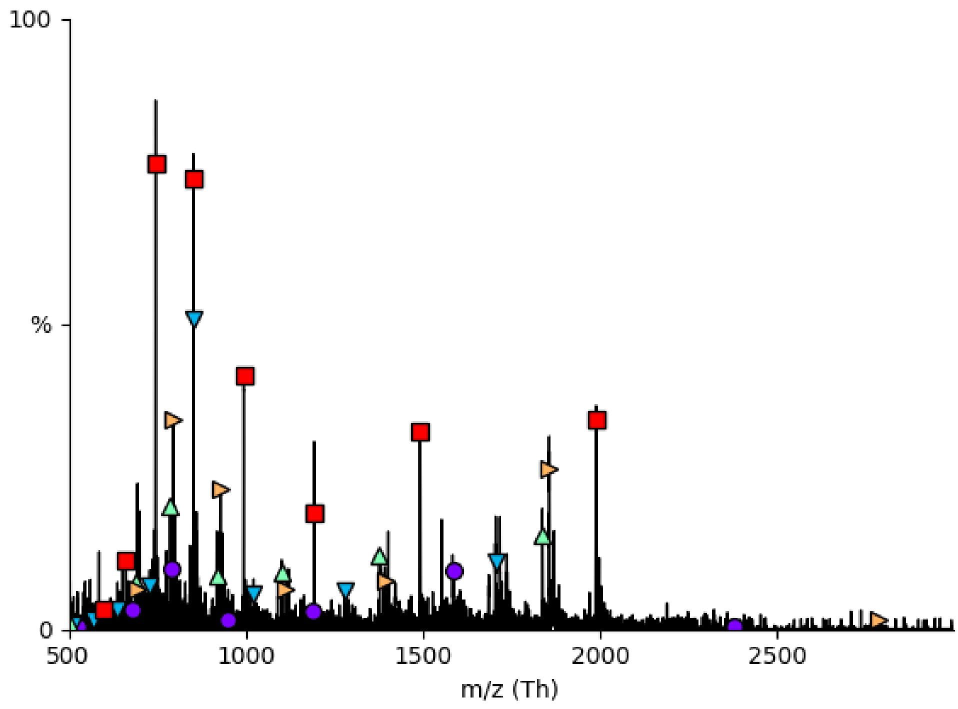

Figure S39 Raw MS spectrum of ON10^{A*}.

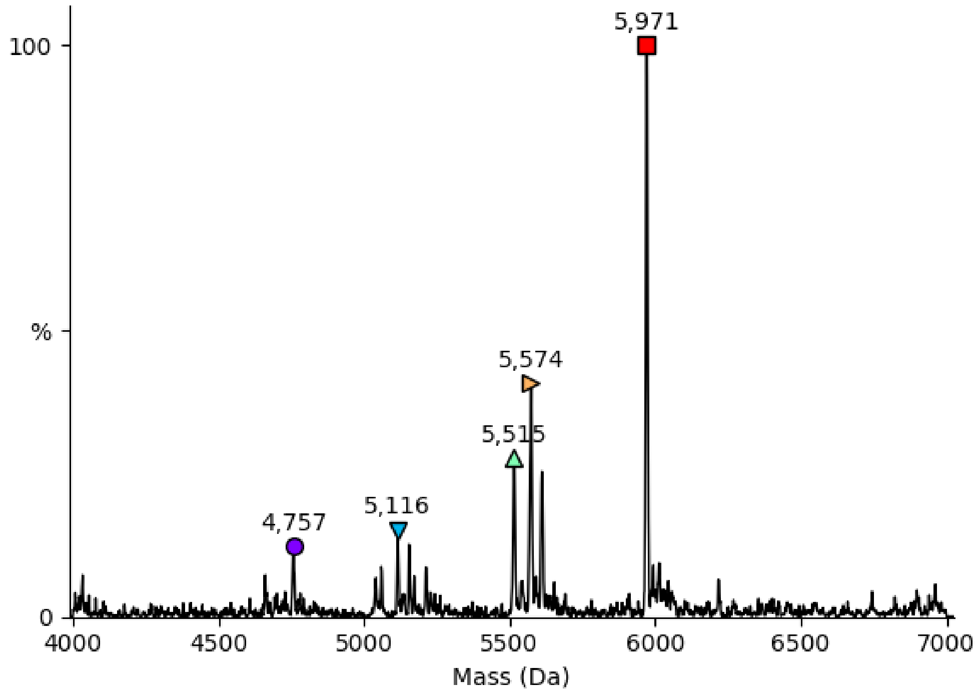

Figure S40 Deconvoluted MS spectrum of ON10^{A*}: calculated: 5973.31 Da; found: 5971 Da, $\Delta = 2.31$ Da. Mass 5574 Da corresponding to full length sequence minus modified G or U phosphate, mass 5115 Da corresponding to minus modified G or U phosphate and minus modified A phosphate.

3.4 Melting and Annealing Curves Measured by UV-vis Absorption

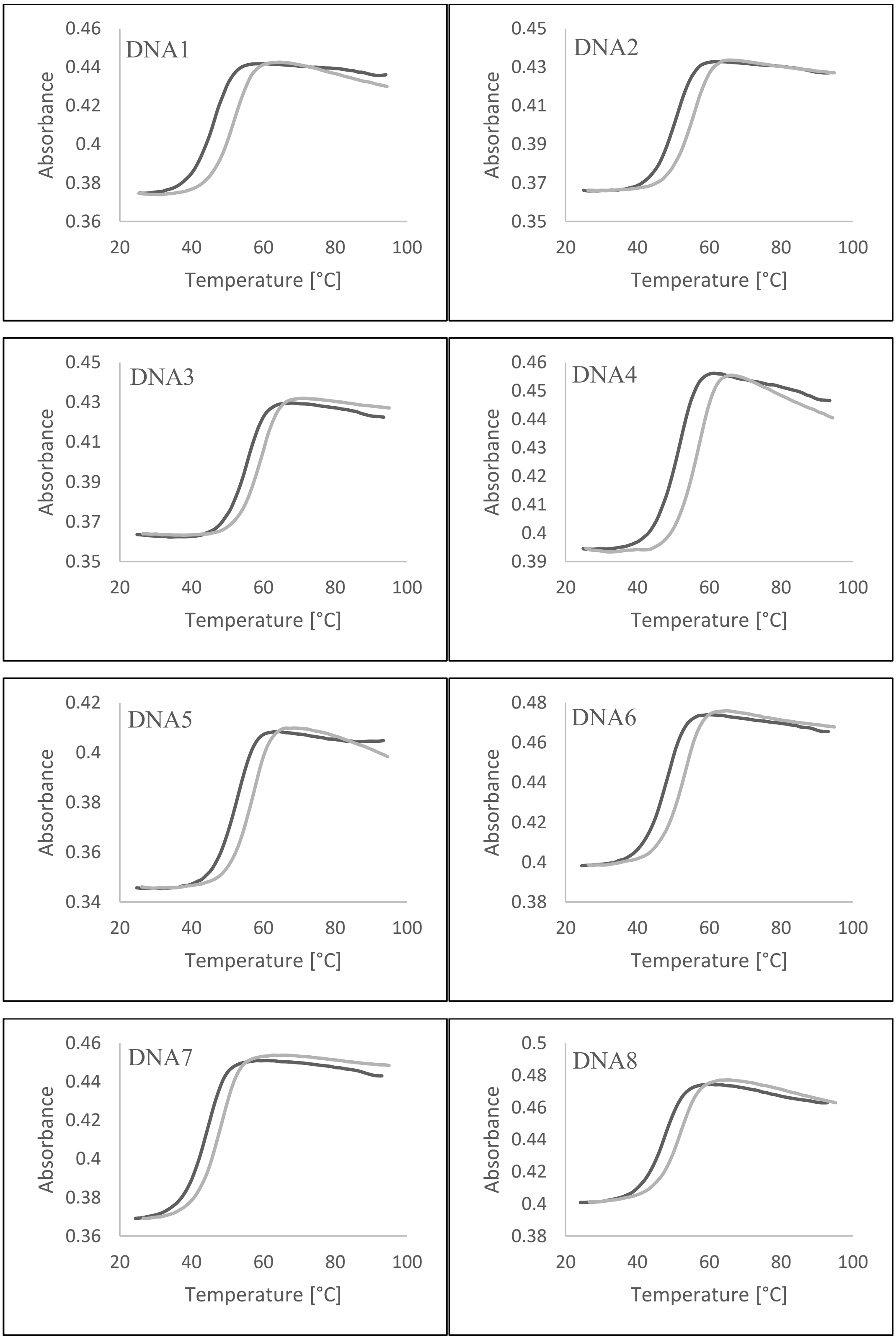

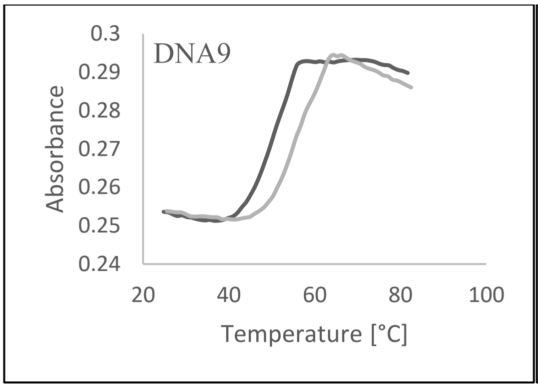
DNA9
Absorbance
0.3
0.29
0.28
0.27
0.26
0.25
0.24
20
40
60
80
100
Temperature [°C]

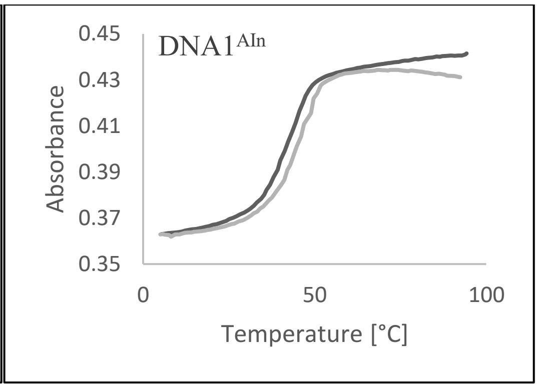
DNA1^{AIn}
Absorbance
0.45
0.43
0.41
0.39
0.37
0.35
0
50
100
Temperature [°C]

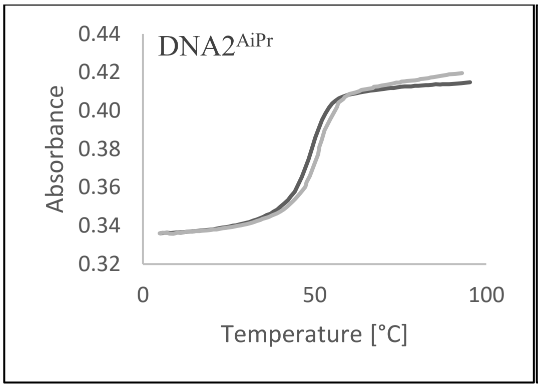
DNA2^{AiPr}
Absorbance
0.44
0.42
0.40
0.38
0.36
0.34
0.32
0
50
100
Temperature [°C]

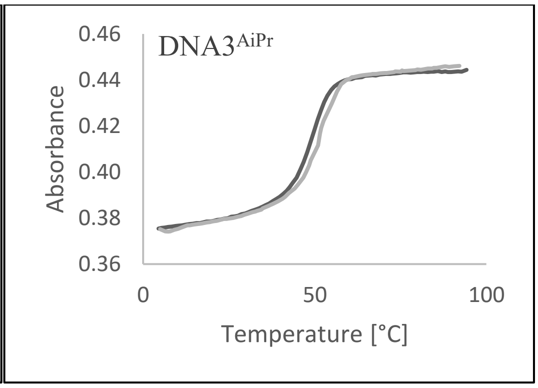
DNA3^{AiPr}
Absorbance
0.46
0.44
0.42
0.40
0.38
0.36
0
50
100
Temperature [°C]

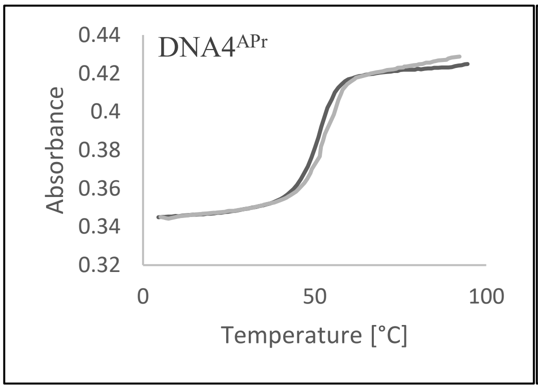
DNA4^{APr}
Absorbance
0.44
0.42
0.4
0.38
0.36
0.34
0.32
0
50
100
Temperature [°C]

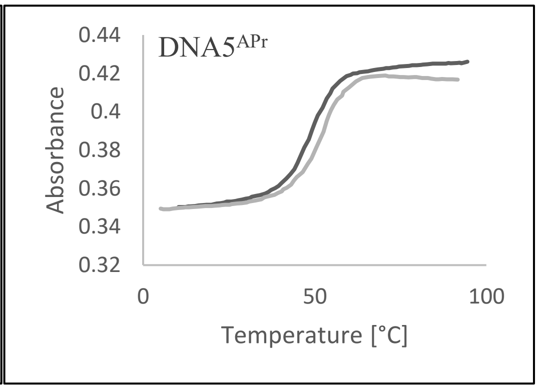
DNA5^{APr}
Absorbance
0.44
0.42
0.4
0.38
0.36
0.34
0.32
0
50
100
Temperature [°C]

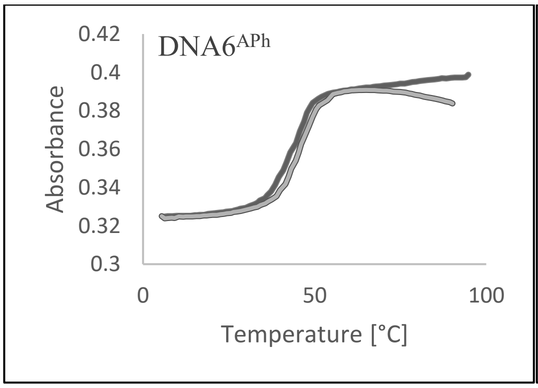
DNA6^{APh}
Absorbance
0.42
0.4
0.38
0.36
0.34
0.32
0.3
0
50
100
Temperature [°C]

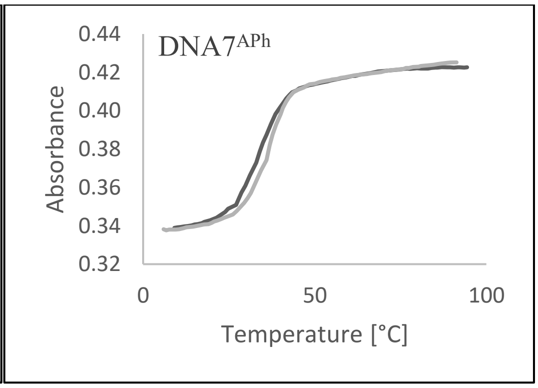
DNA7^{APh}
Absorbance
0.44
0.42
0.40
0.38
0.36
0.34
0.32
0
50
100
Temperature [°C]

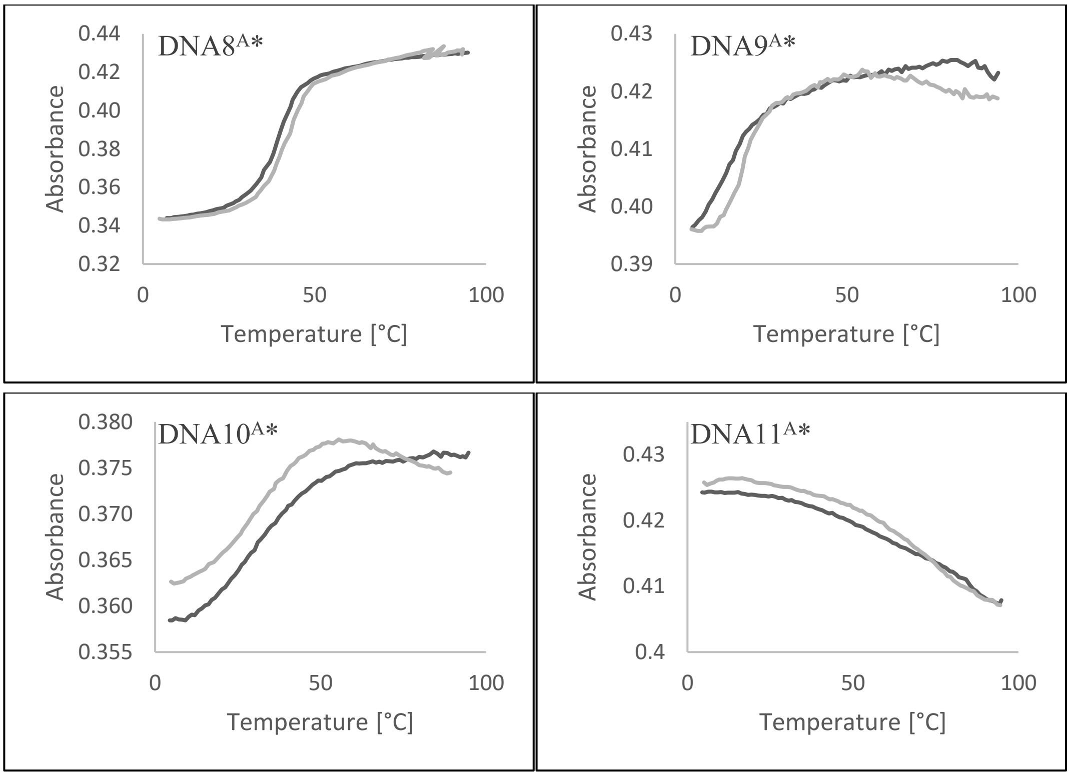
DNA8A*
DNA9A*
DNA10A*
DNA11A*
Absorbance
Temperature [°C]
0.44
0.42
0.40
0.38
0.36
0.34
0.32
0.43
0.41
0.39
0.380
0.375
0.370
0.365
0.360
0.355
0.4
0
50
100

3.5 Melting Temperatures Measured by Circular Dichroism Spectroscopy

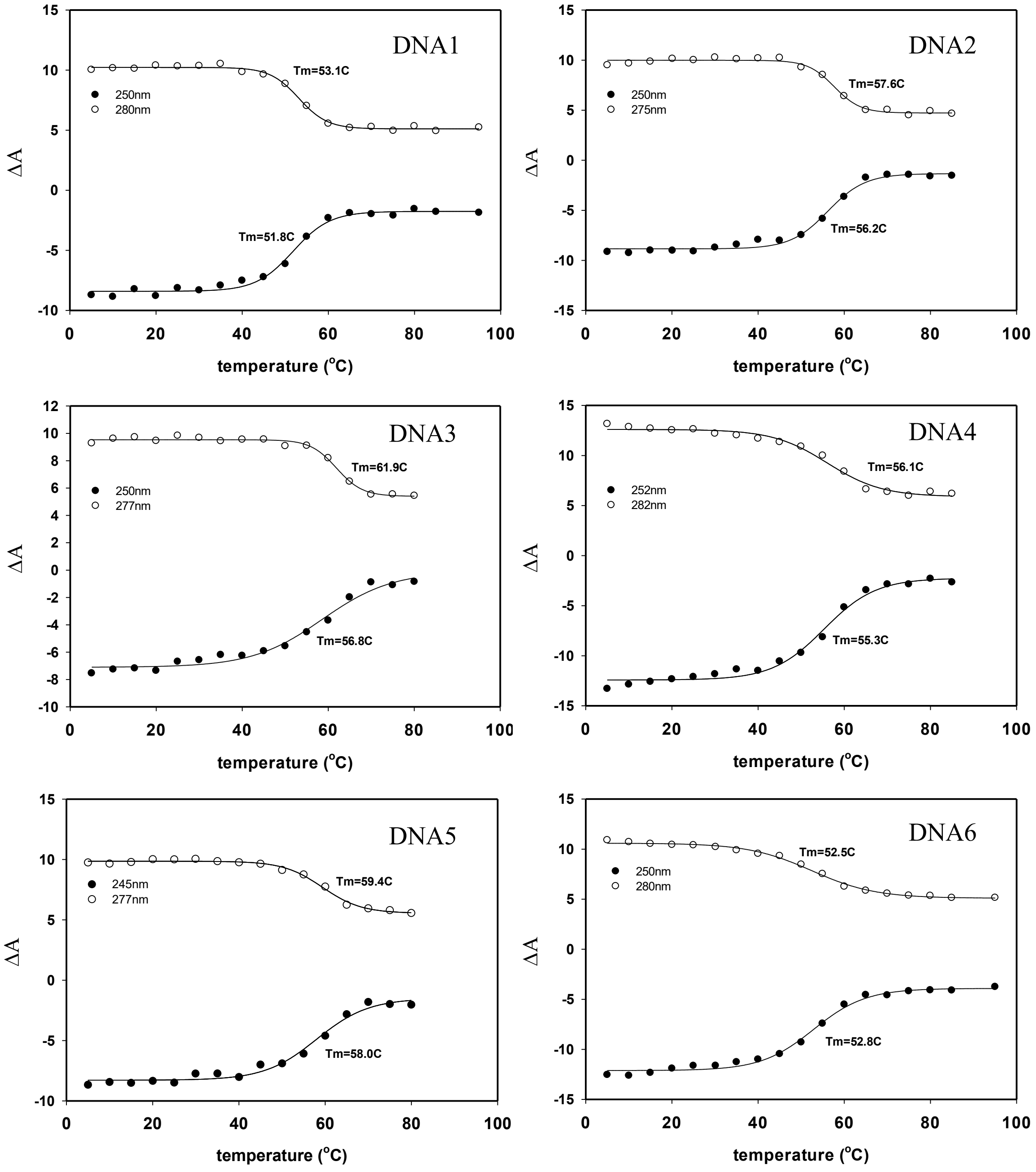

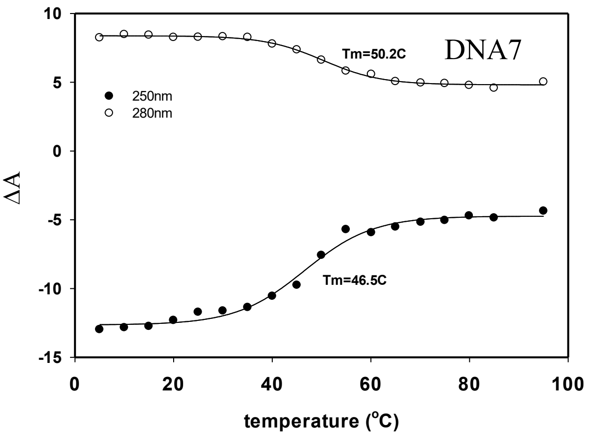
DNA7
Tm=50.2C
Tm=46.5C
250nm
280nm
ΔA
temperature (°C)

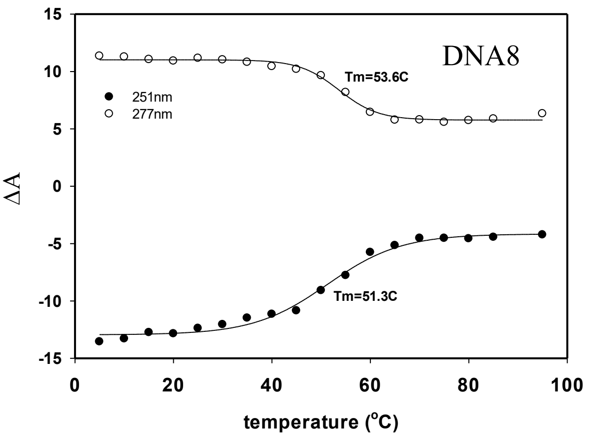
DNA8
Tm=53.6C
Tm=51.3C
251nm
277nm
ΔA
temperature (°C)

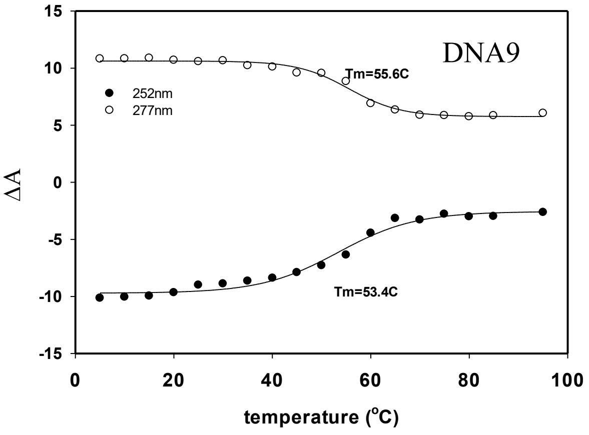
DNA9
Tm=55.6C
Tm=53.4C
252nm
277nm
ΔA
temperature (°C)

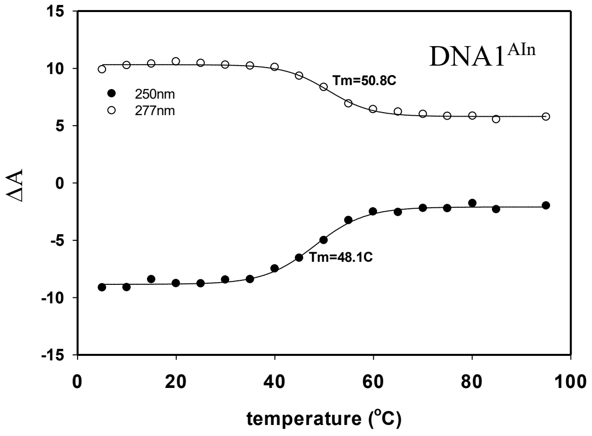
DNA1AIn
Tm=50.8C
Tm=48.1C
250nm
277nm
ΔA
temperature (°C)

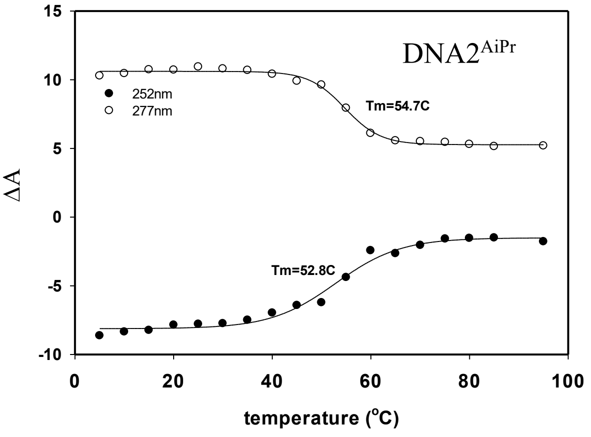
DNA2AiPr
Tm=54.7C
Tm=52.8C
252nm
277nm
ΔA
temperature (°C)

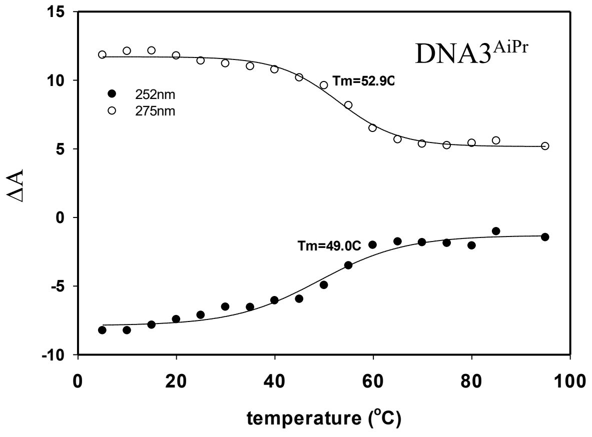
DNA3AiPr
Tm=52.9C
Tm=49.0C
252nm
275nm
ΔA
temperature (°C)

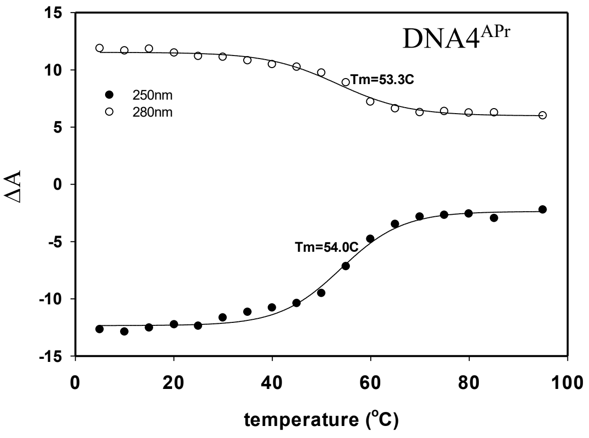
DNA4APr
Tm=53.3C
Tm=54.0C
250nm
280nm
ΔA
temperature (°C)

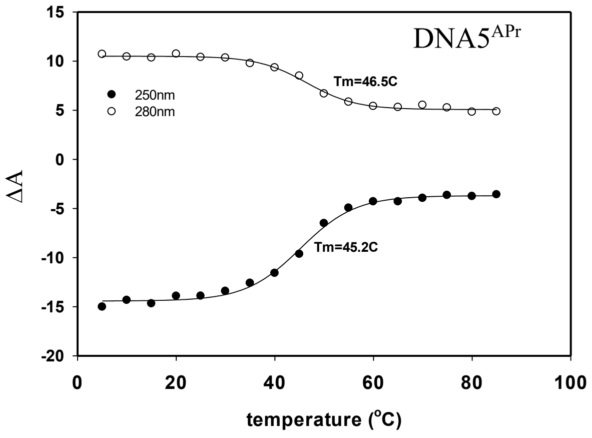
DNA5APr
Tm=46.5C
Tm=45.2C
250nm
280nm
ΔA
temperature (°C)

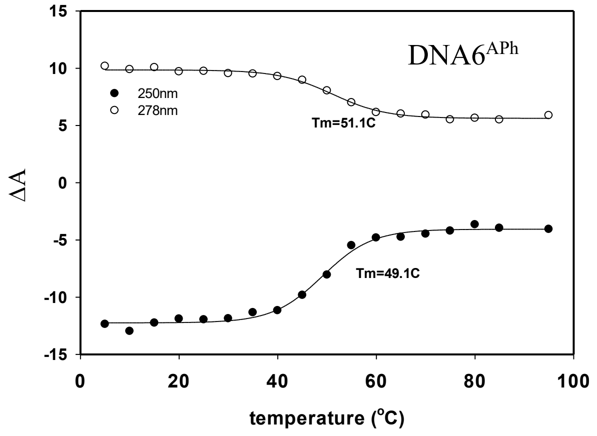
DNA6APh
250nm
278nm
Tm=51.1C
Tm=49.1C
ΔA
temperature (oC)

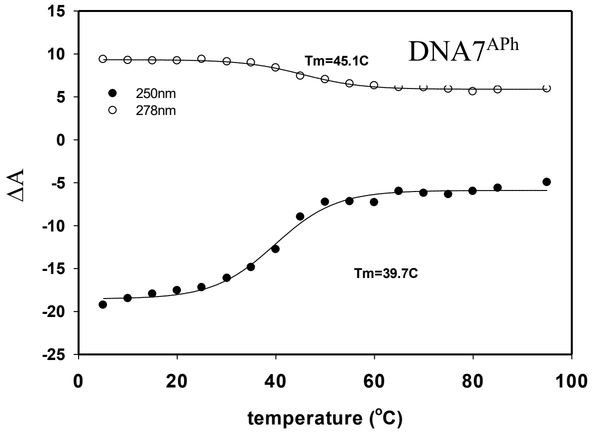
DNA7APh
Tm=45.1C
250nm
278nm
Tm=39.7C
ΔA
temperature (oC)

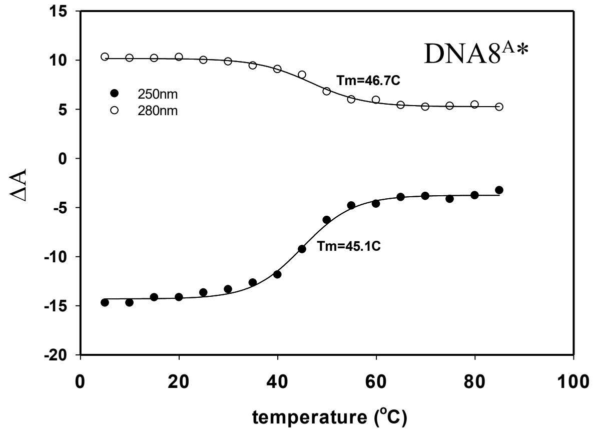
DNA8A*
Tm=46.7C
250nm
280nm
Tm=45.1C
ΔA
temperature (oC)

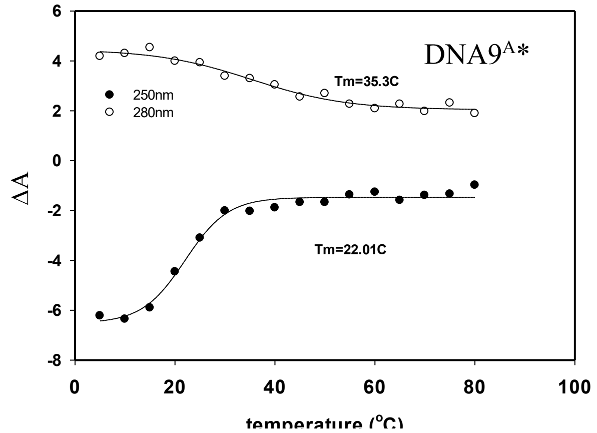
DNA9A*
Tm=35.3C
250nm
280nm
Tm=22.01C
ΔA

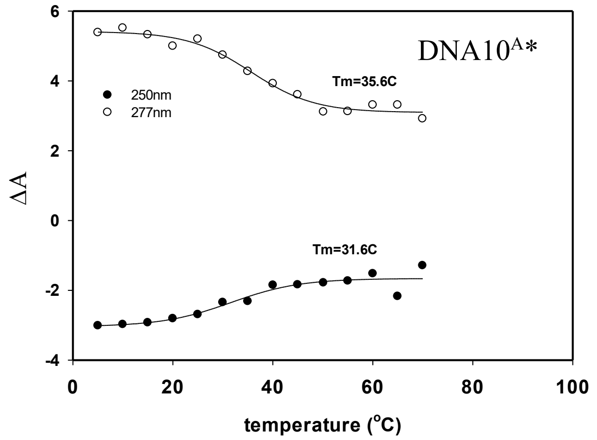
DNA10A*
Tm=35.6C
250nm
277nm
Tm=31.6C
ΔA
temperature (oC)

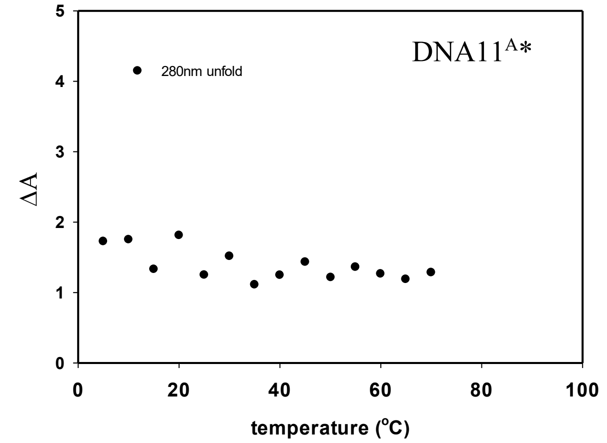
DNA11A*
280nm unfold
ΔA
temperature (oC)

3.6 Circular Dichroism Spectroscopy Spectra

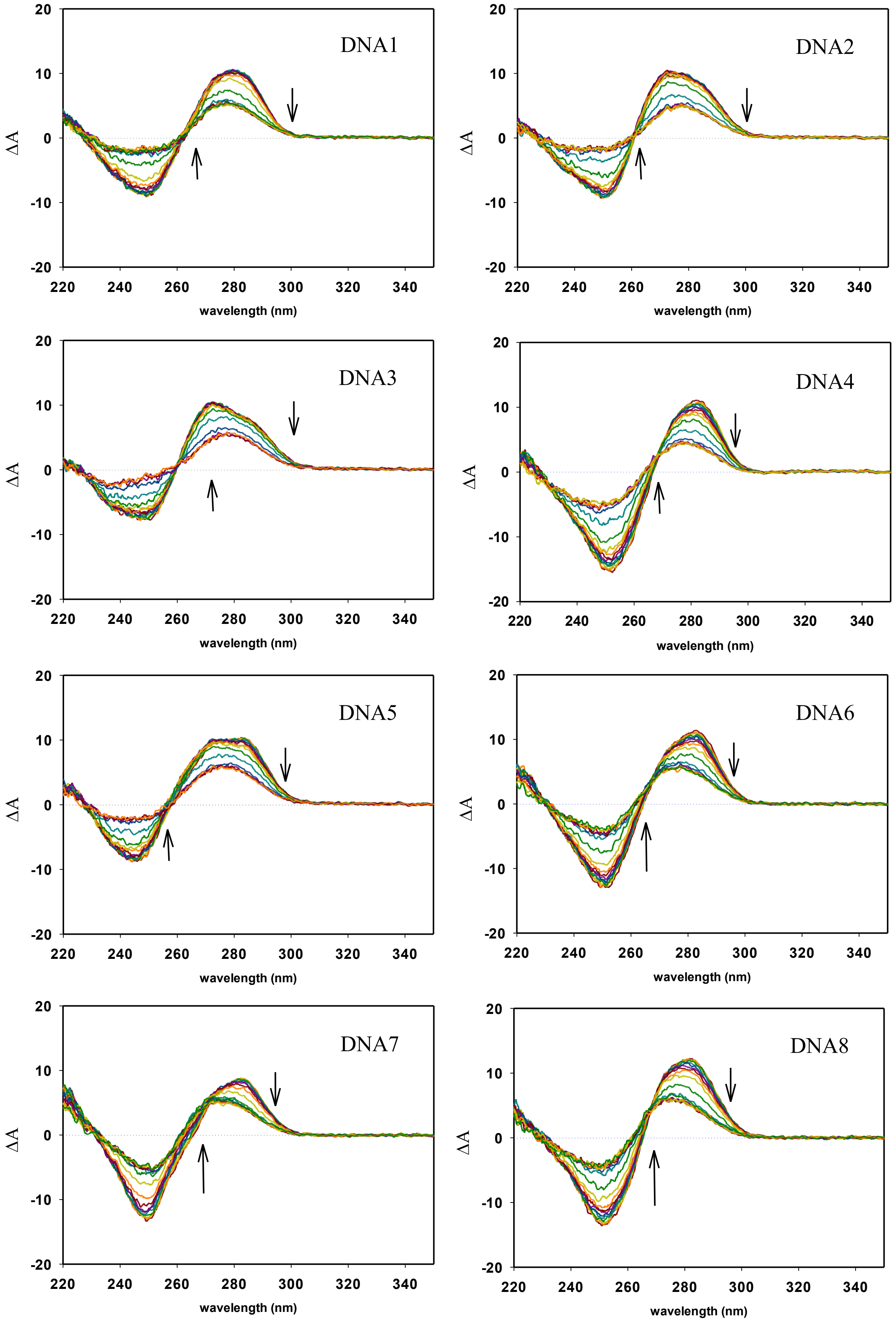

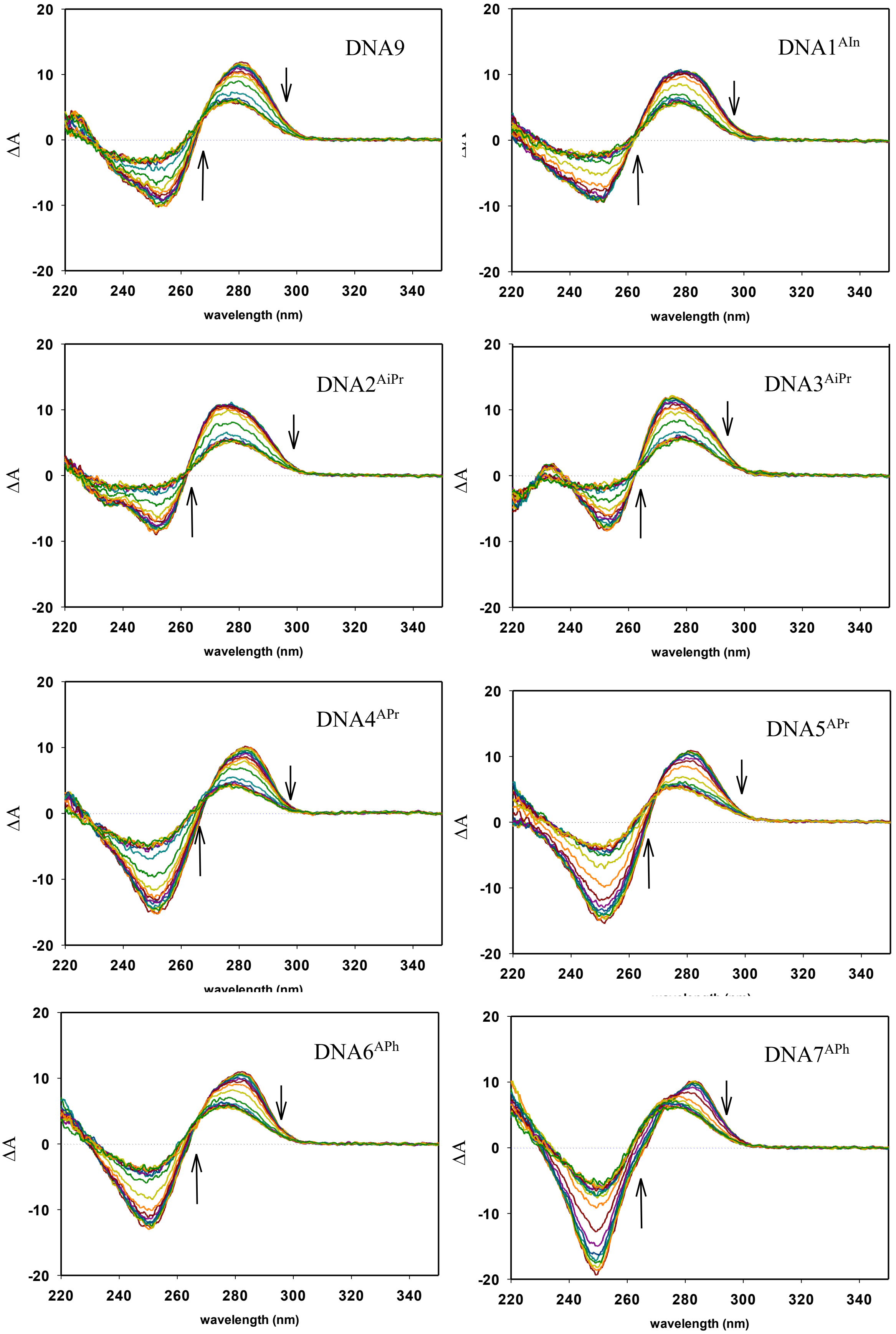
DNA9
DNA1^{AIn}
DNA2^{AiPr}
DNA3^{AiPr}
DNA4^{APr}
DNA5^{APr}
DNA6^{APh}
DNA7^{APh}
ΔA
wavelength (nm)
20
10
0
-10
-20
220
240
260
280
300
320
340

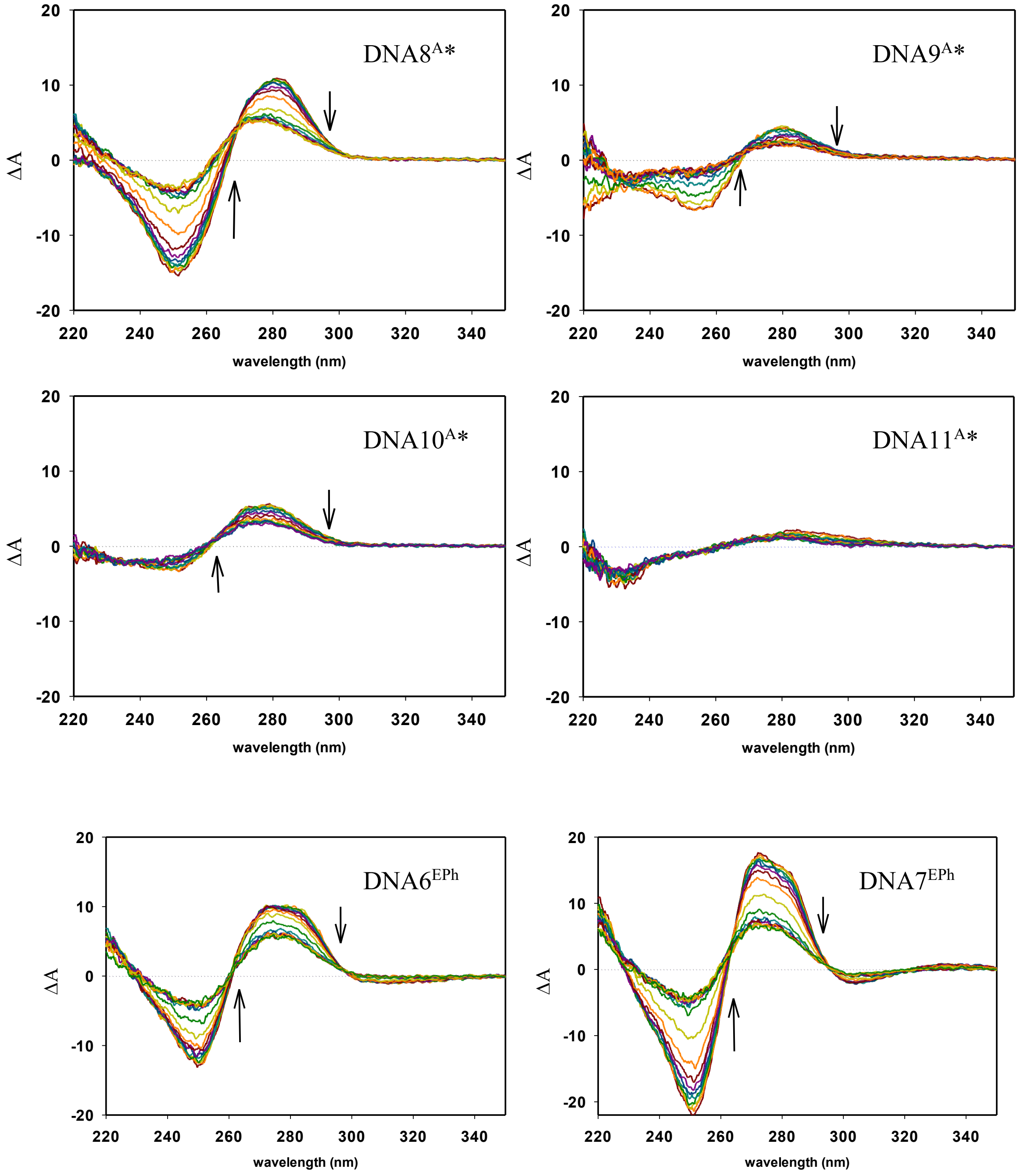
DNA8A*
DNA9A*
DNA10A*
DNA11A*
DNA6EPh
DNA7EPh
ΔA
wavelength (nm)
20
10
0
-10
-20
220
240
260
280
300
320
340

3.7 Copies of NMR Spectra

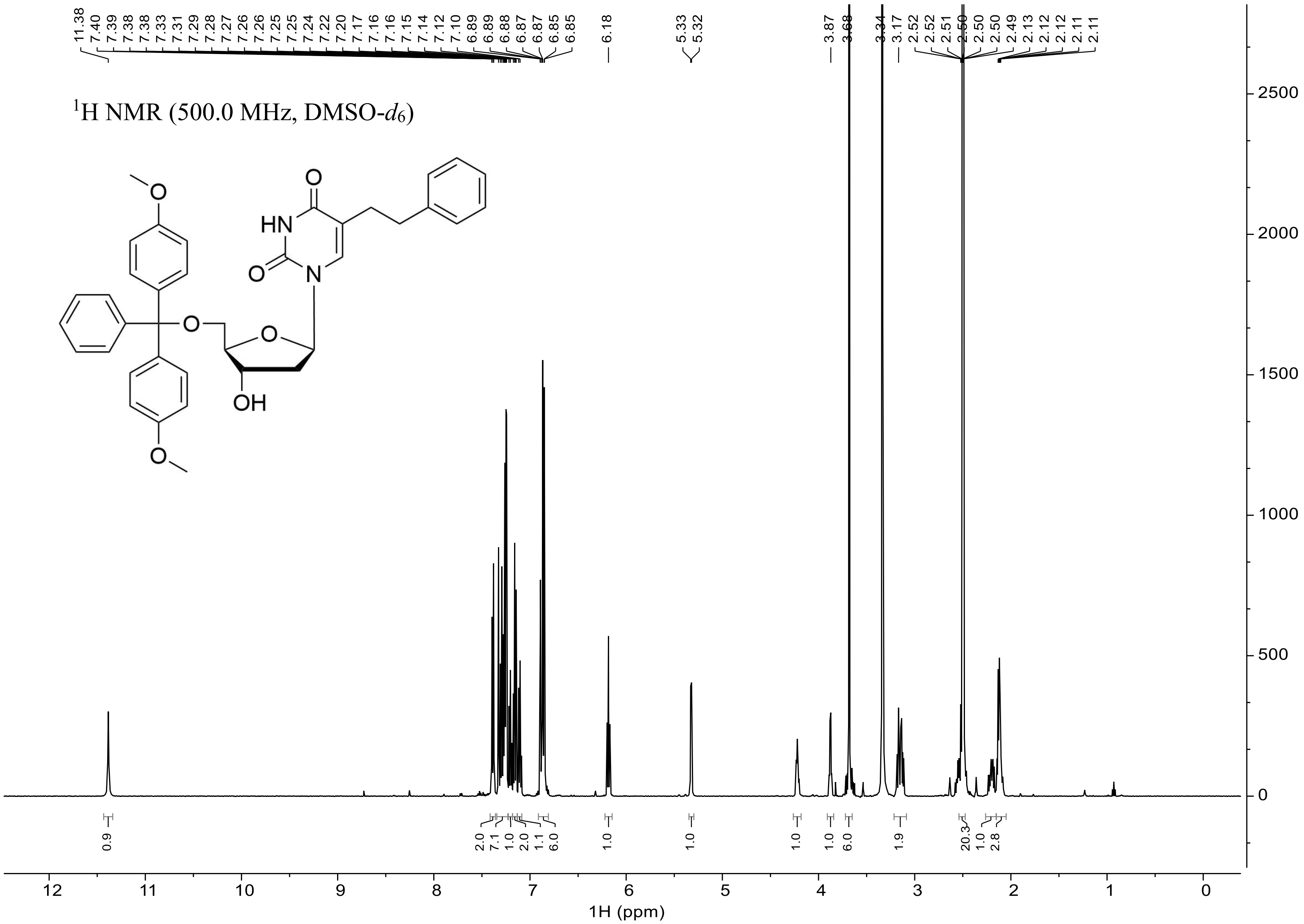

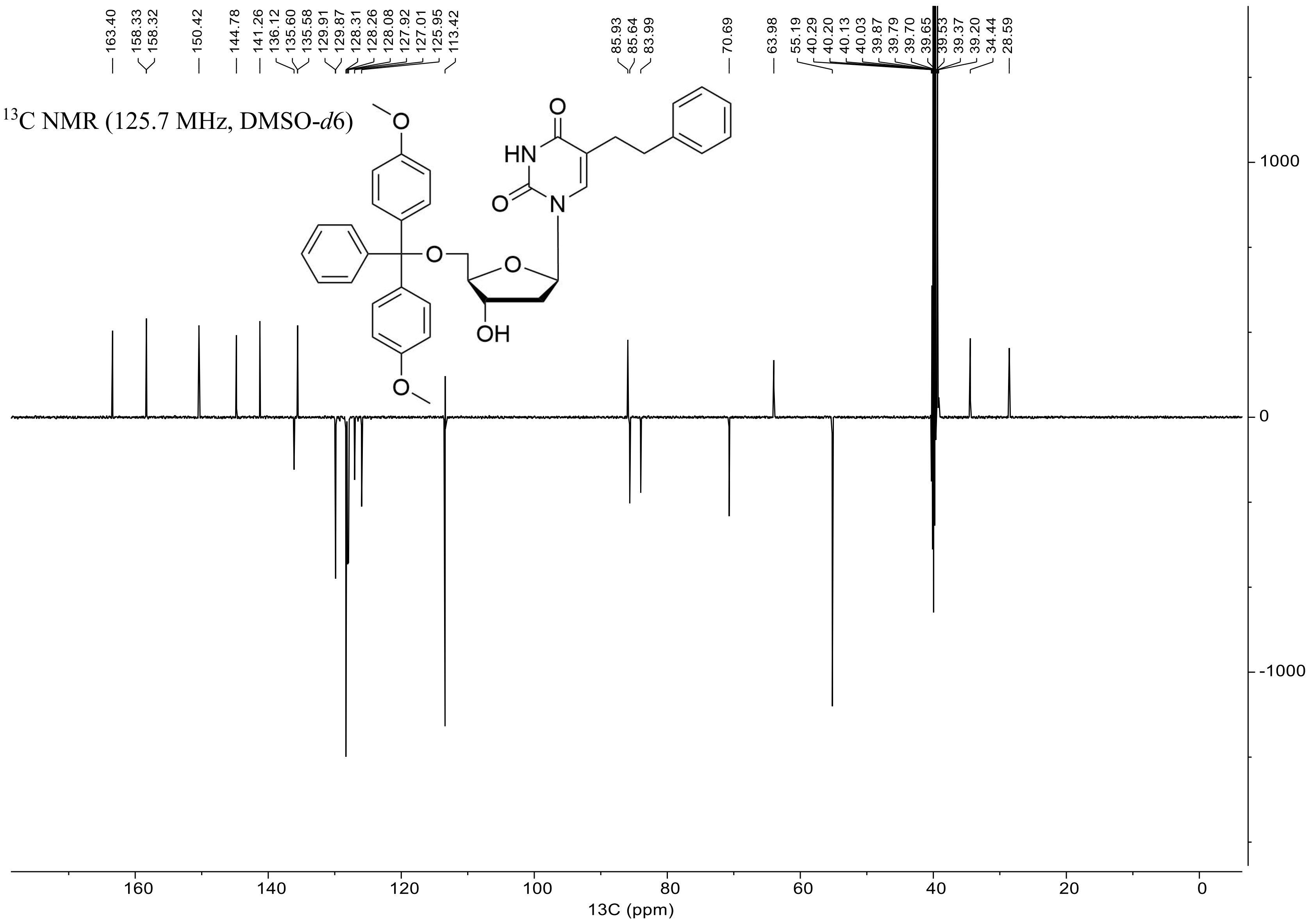
13C NMR (125.7 MHz, DMSO-d6)
163.40
158.33
158.32
150.42
144.78
141.26
136.12
135.60
135.58
129.91
129.87
128.31
128.26
128.08
127.92
127.01
125.95
113.42
85.93
85.64
83.99
70.69
63.98
55.19
40.29
40.20
40.13
40.03
39.87
39.79
39.70
39.65
39.53
39.37
39.20
34.44
28.59
HN
O
N
OH
1000
0
-1000
160
140
120
100
80
60
40
20
0
13C (ppm)

^{1}H NMR (500.0 MHz, CD_3CN)

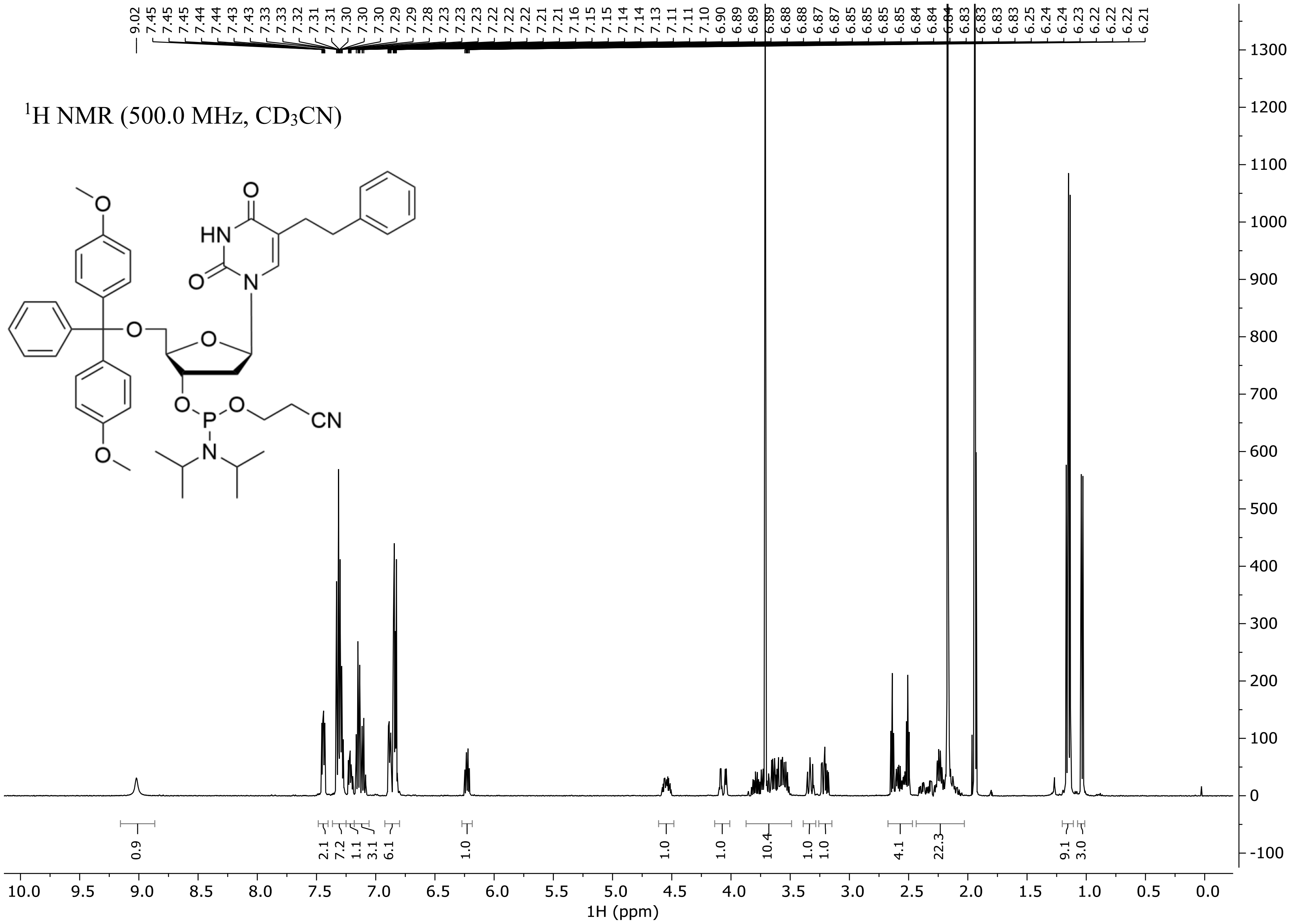

¹³C NMR (125.7 MHz, CD_3CN)

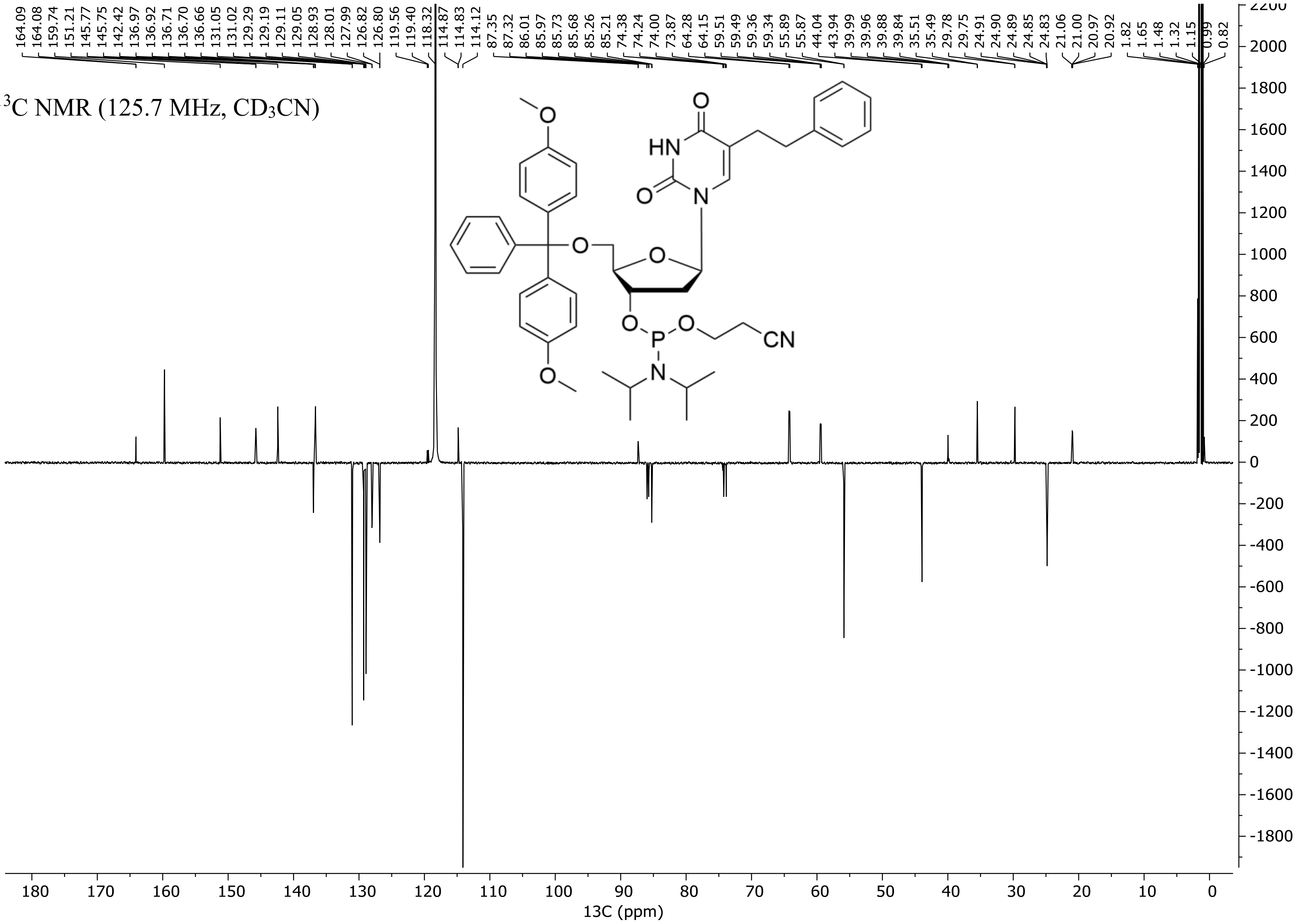

$^{31}P\{^{1}H\}$ NMR (202.4 MHz, CD_3CN)

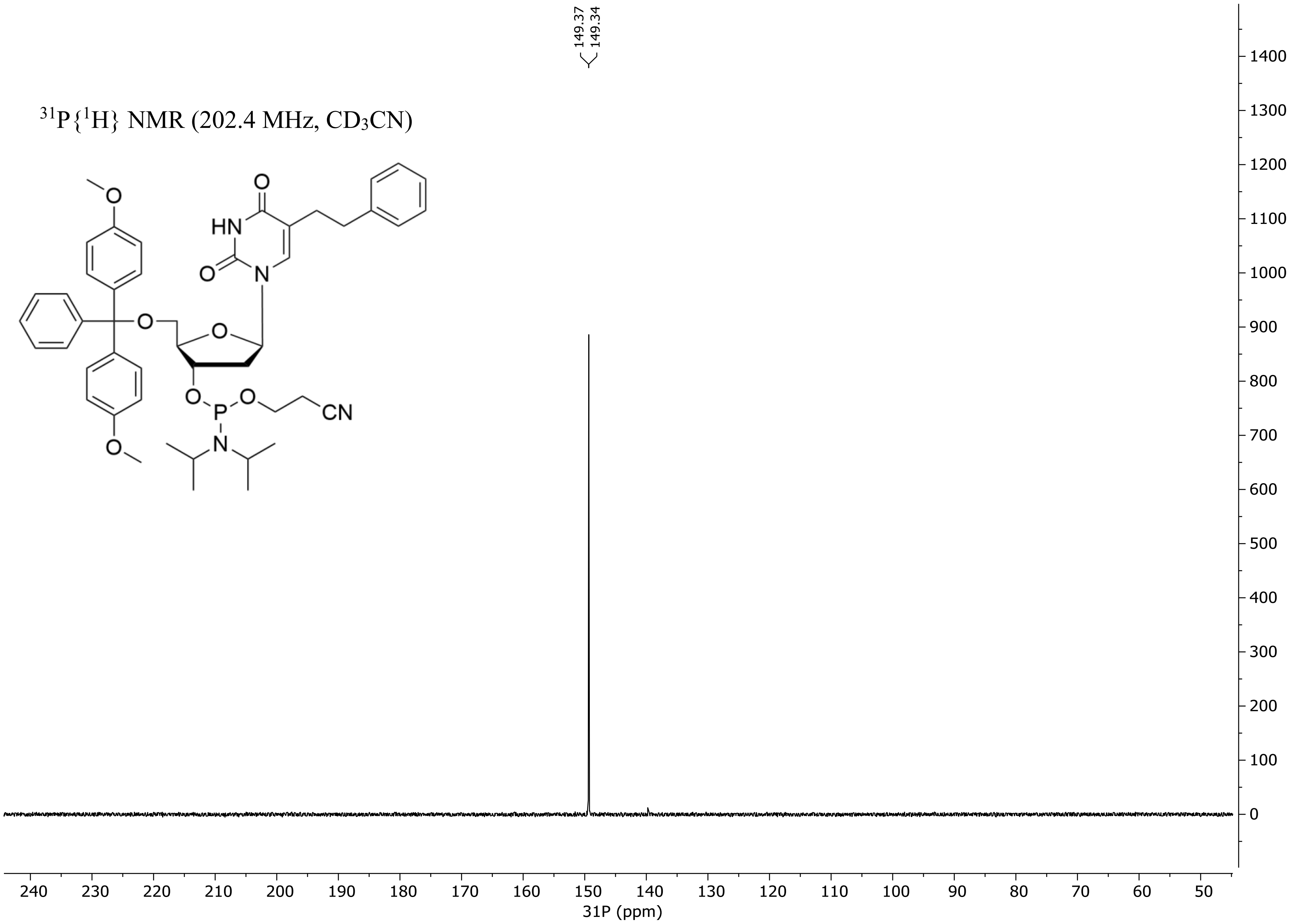

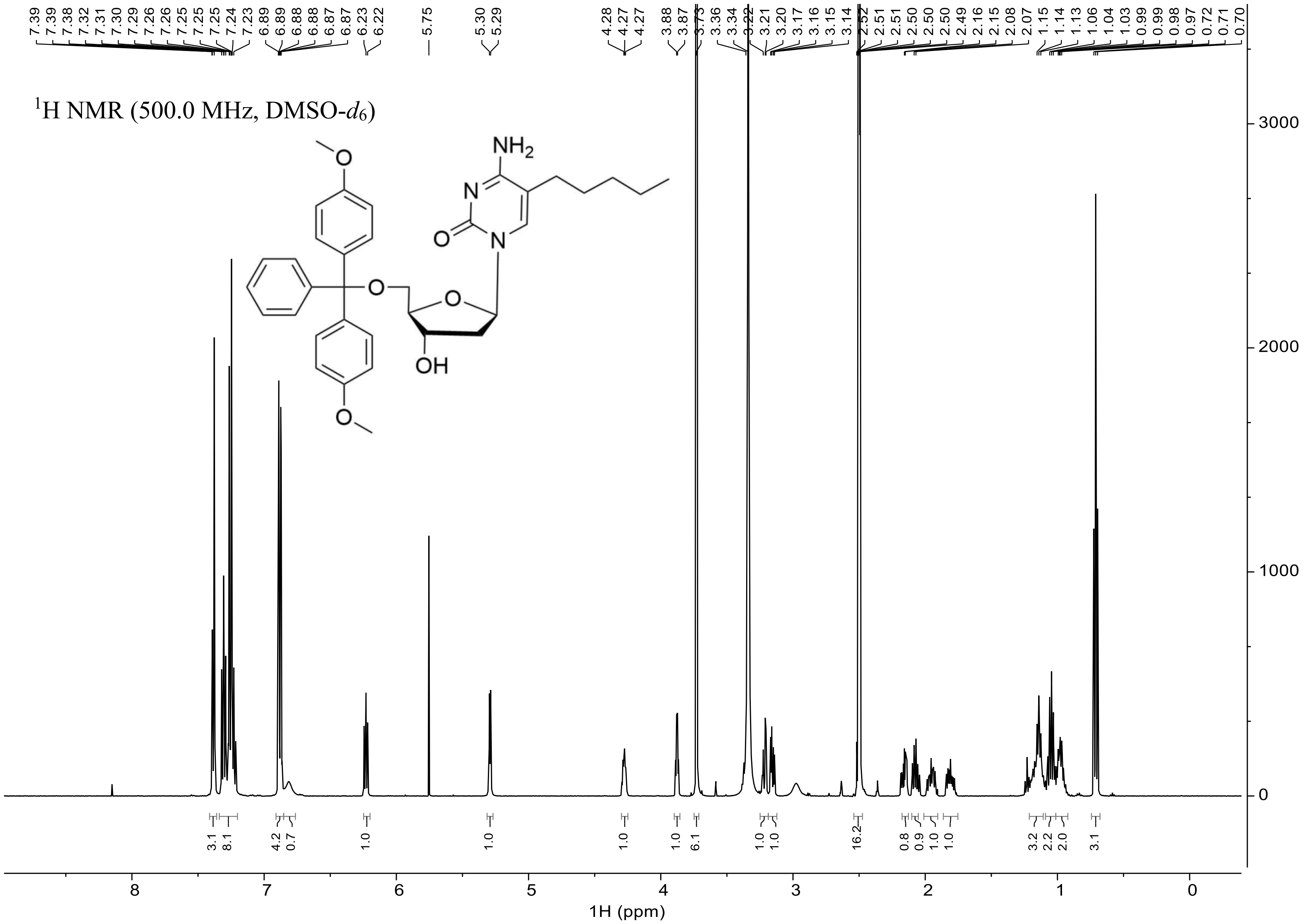

^{1}H NMR (500.0 MHz, DMSO-d_6)
NH2
N
O
N
O
O
OH
O
O
1H (ppm)

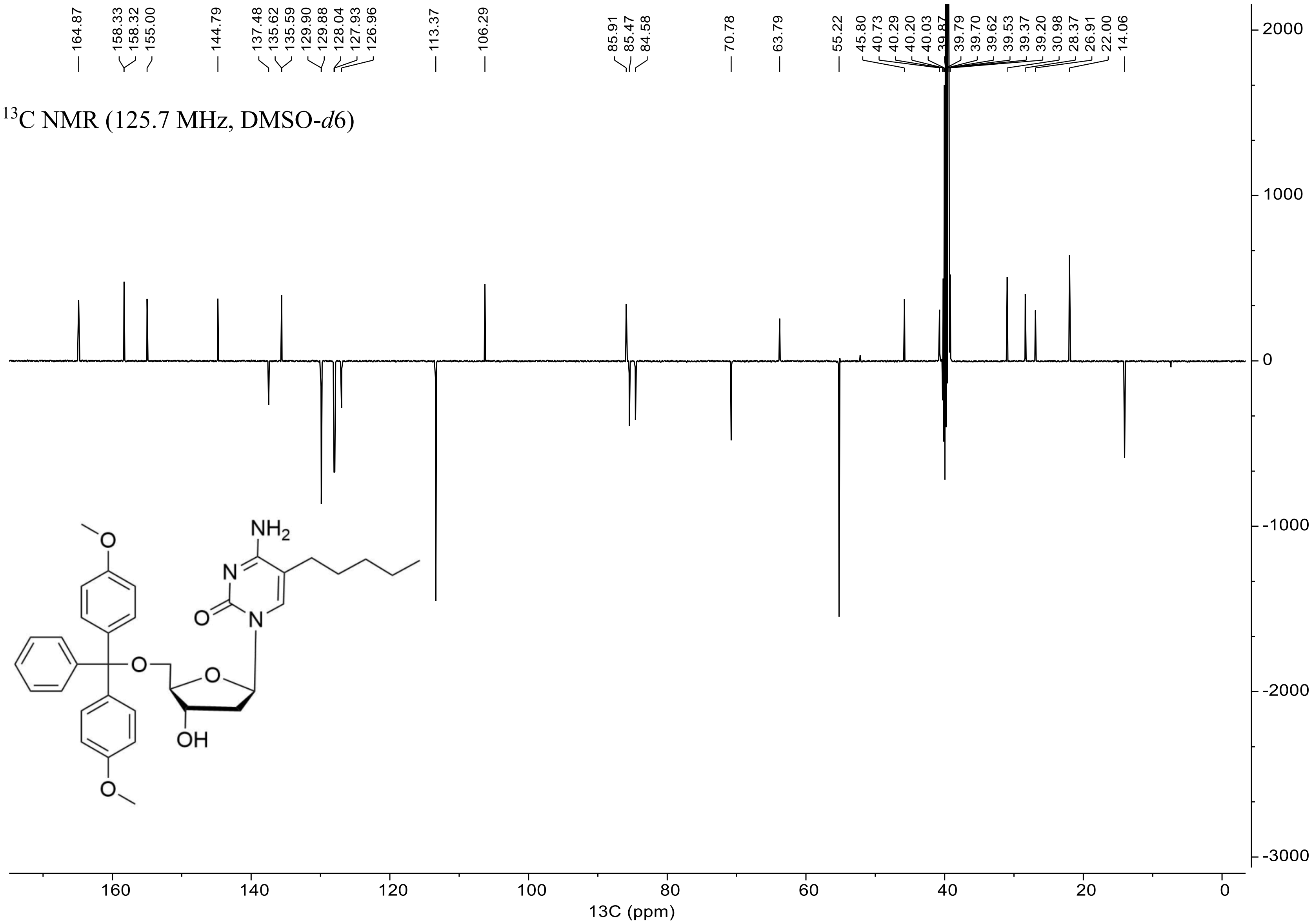
164.87
158.33
158.32
155.00
144.79
137.48
135.62
135.59
129.90
129.88
128.04
127.93
126.96
113.37
106.29
85.91
85.47
84.58
70.78
63.79
55.22
45.80
40.73
40.29
40.20
40.03
39.79
39.70
39.62
39.53
39.37
39.20
30.98
28.37
26.91
22.00
14.06
^{13}C NMR (125.7 MHz, DMSO-*d*6)
NH2
N
O
N
O
O
O
OH
13C (ppm)

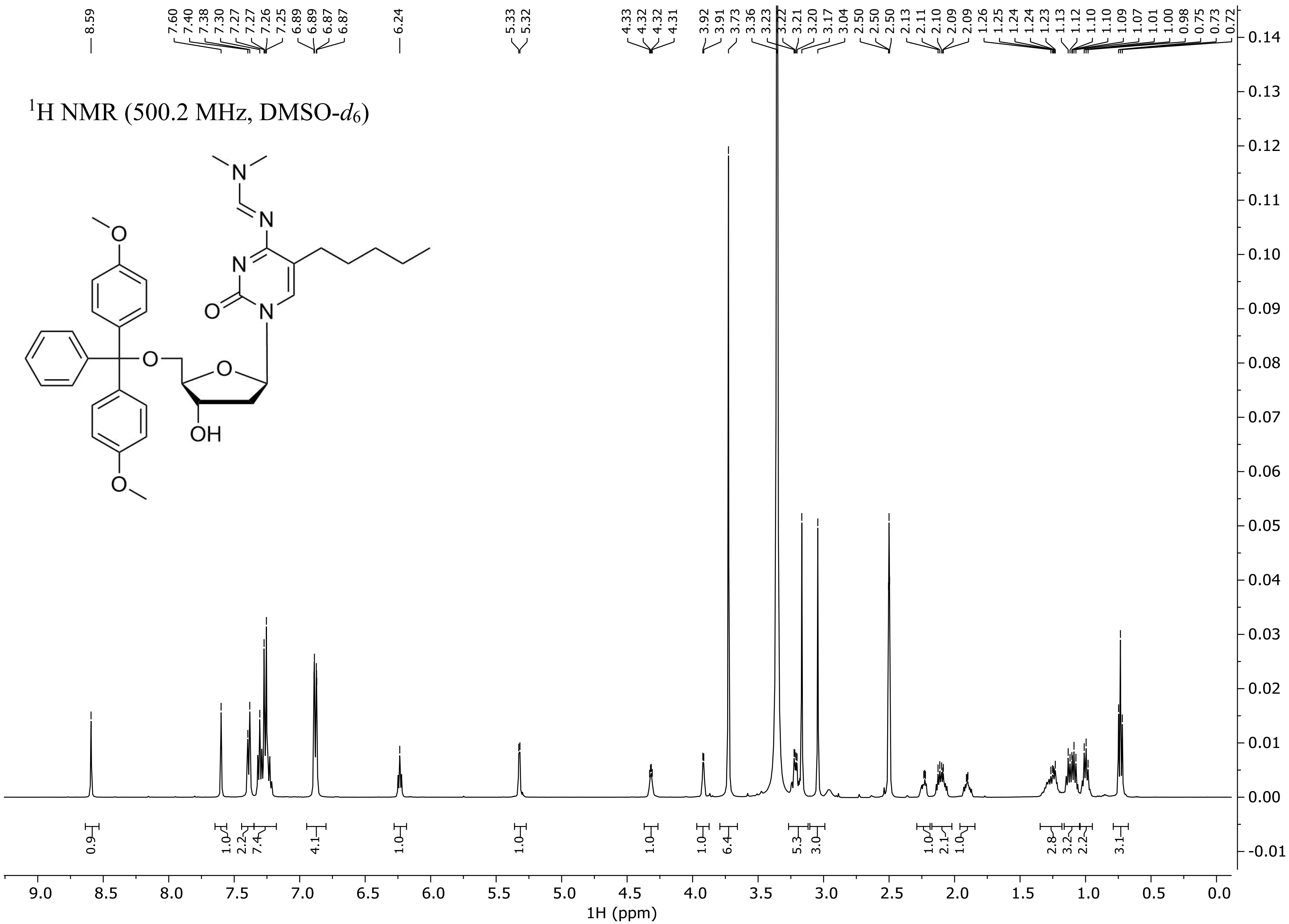

1H NMR (500.2 MHz, DMSO-d6)
1H (ppm)

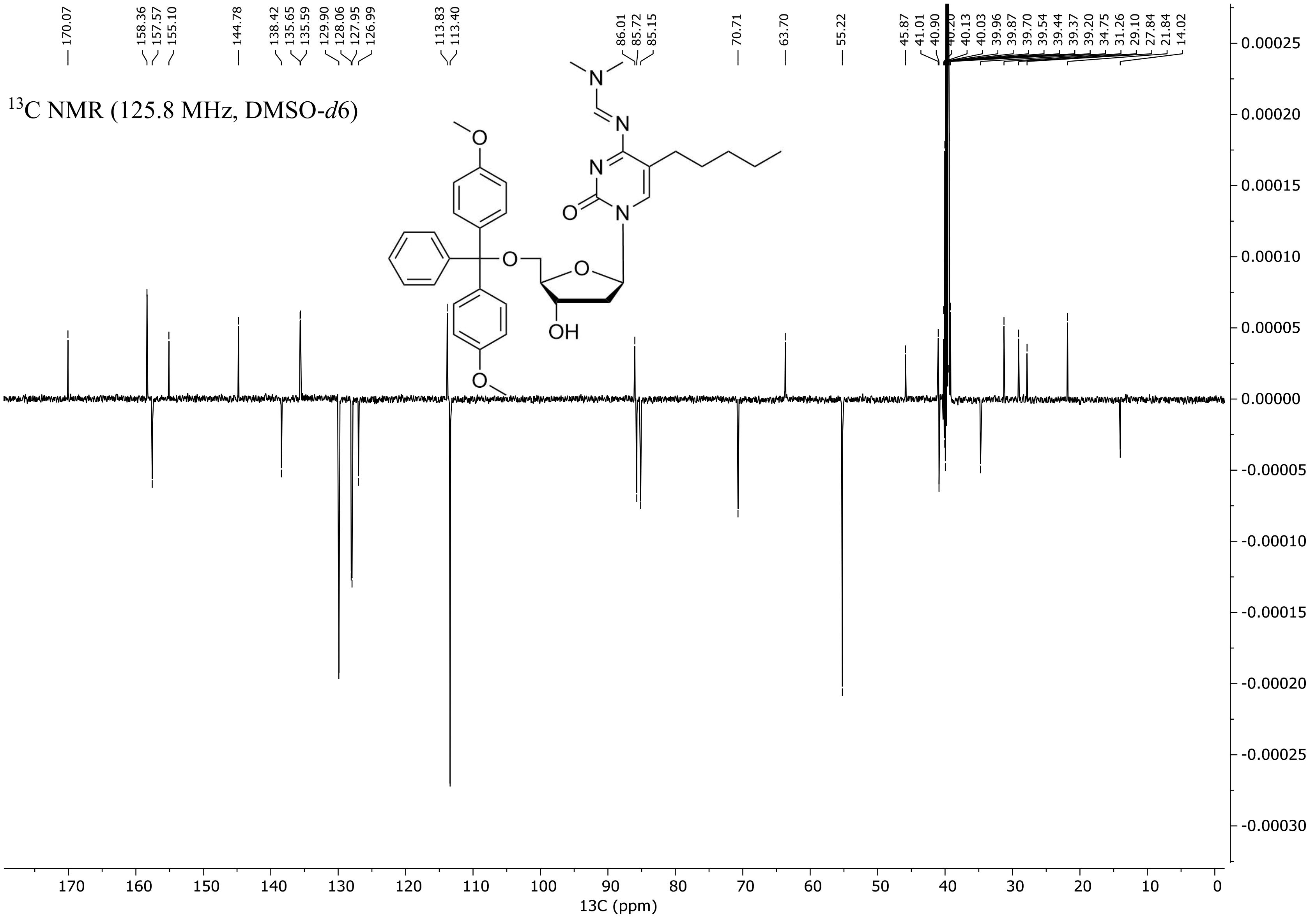

170.07
158.36
157.57
155.10
144.78
138.42
135.65
135.59
129.90
128.06
127.95
126.99
113.83
113.40
86.01
85.72
85.15
70.71
63.70
55.22
45.87
41.01
40.90
40.13
40.03
39.96
39.87
39.70
39.54
39.44
39.37
39.20
34.75
31.26
29.10
27.84
21.84
14.02
^{13}C NMR (125.8 MHz, DMSO-*d*6)
13C (ppm)

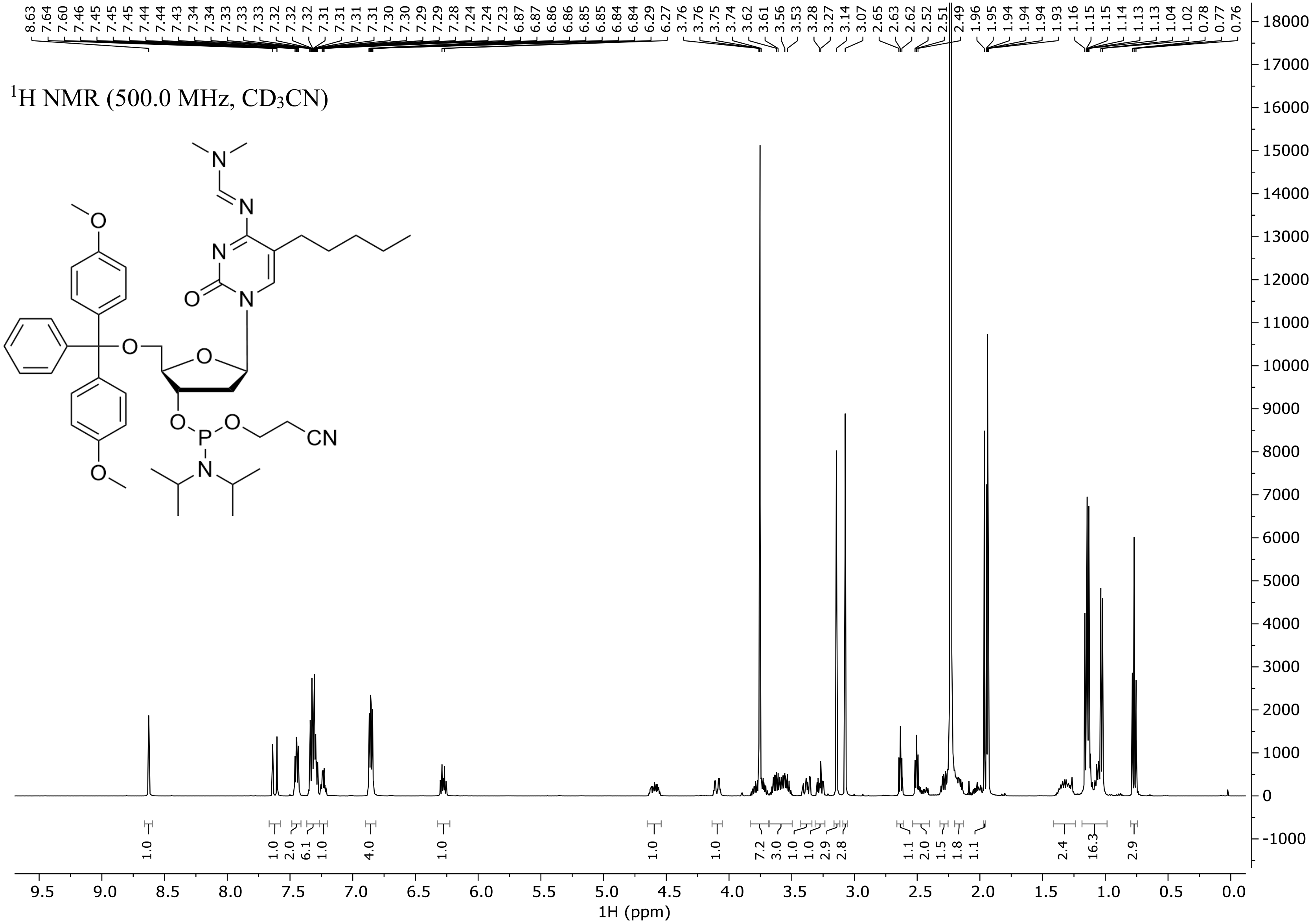

1H NMR (500.0 MHz, CD3CN)
8.63 7.64 7.60 7.46 7.45 7.45 7.45 7.44 7.44 7.43 7.34 7.34 7.33 7.33 7.33 7.32 7.32 7.32 7.31 7.31 7.31 7.31 7.30 7.30 7.29 7.29 7.28 7.24 7.24 7.23 6.87 6.87 6.86 6.86 6.85 6.85 6.84 6.84 6.29 6.27 3.76 3.76 3.75 3.74 3.62 3.61 3.56 3.53 3.28 3.27 3.14 3.07 2.65 2.63 2.62 2.52 2.51 2.49 1.96 1.95 1.94 1.94 1.94 1.93 1.16 1.15 1.15 1.14 1.13 1.13 1.04 1.02 0.78 0.77 0.76
1.0 1.0 2.0 6.1 1.0 4.0 1.0 1.0 1.0 7.2 3.0 1.0 1.0 2.9 2.8 1.1 2.0 1.5 1.8 1.1 2.4 16.3 2.9
1H (ppm)

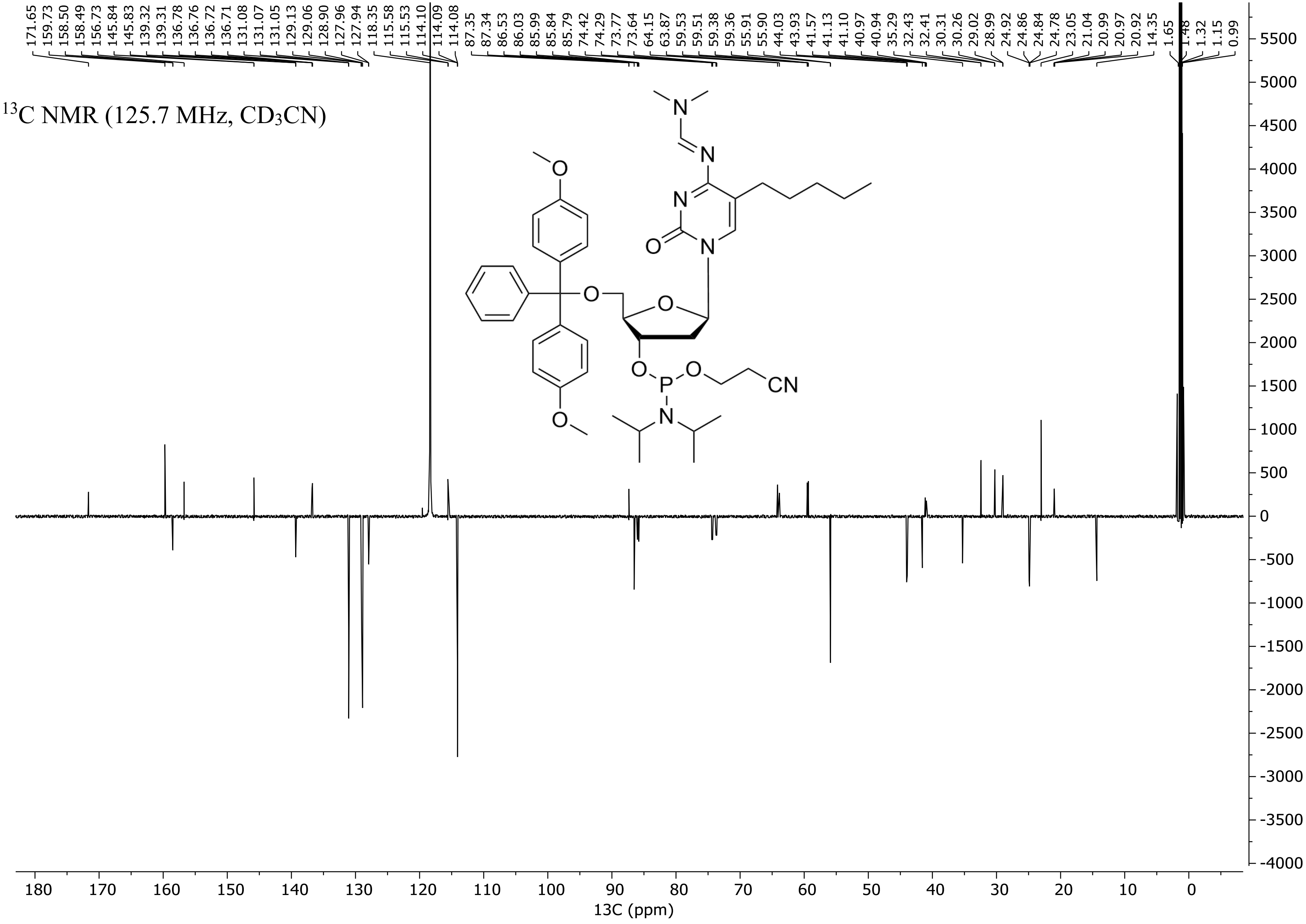
171.65 159.73 158.50 158.49 156.73 145.84 145.83 139.32 139.31 136.78 136.76 136.72 136.71 131.08 131.07 131.05 129.13 129.06 128.90 127.96 127.94 118.35 115.58 115.53 114.10 114.09 114.08 87.35 87.34 86.53 86.03 85.99 85.84 85.79 74.42 74.29 73.77 73.64 64.15 63.87 59.53 59.51 59.38 59.36 55.91 55.90 44.03 43.93 41.57 41.13 41.10 40.97 40.94 35.29 32.43 32.41 30.31 30.26 29.02 28.99 24.92 24.86 24.84 24.78 23.05 21.04 20.99 20.97 20.92 14.35 1.65 1.48 1.32 1.15 0.99
^{13}C NMR (125.7 MHz, CD_3CN)
13C (ppm)

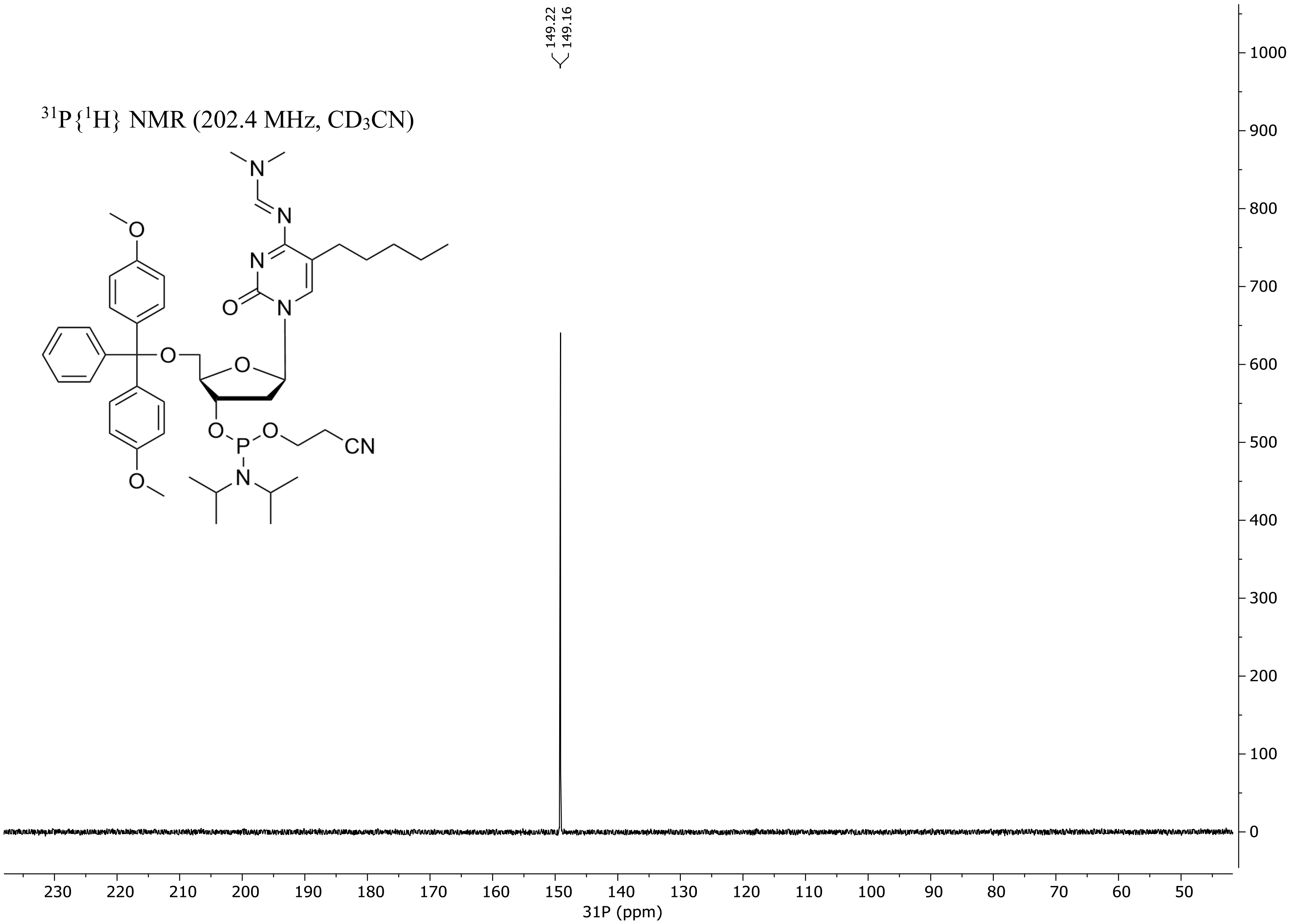
149.22
149.16
$^{31}P\{^{1}H\}$ NMR (202.4 MHz, CD_3CN)
N
N
N
O
N
O
O
O
O
P
O
CN
N
1000
900
800
700
600
500
400
300
200
100
0
230
220
210
200
190
180
170
160
150
140
130
120
110
100
90
80
70
60
50
31P (ppm)

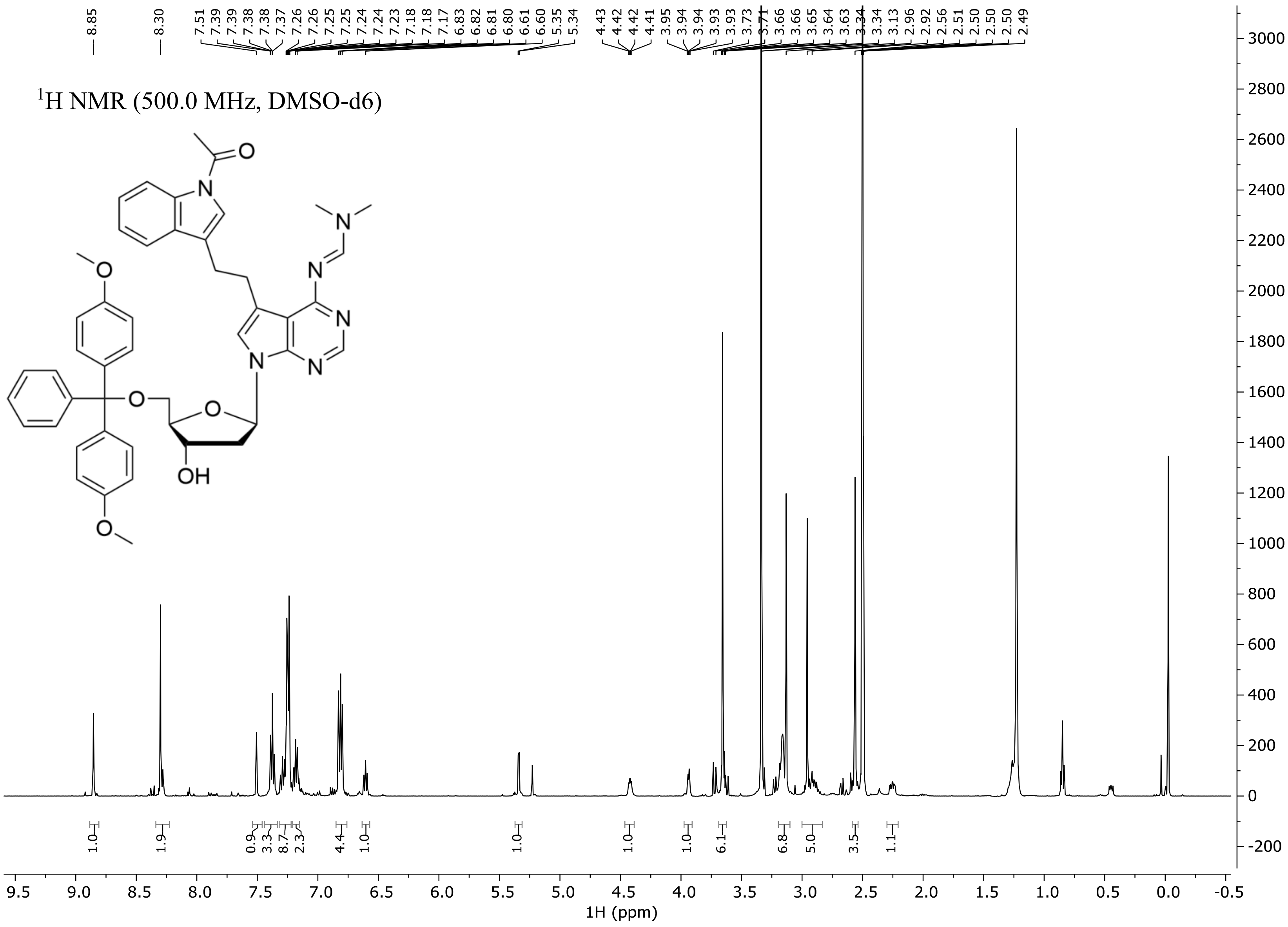

^{1}H NMR (500.0 MHz, DMSO-d6)
8.85
8.30
7.51
7.39
7.39
7.38
7.38
7.37
7.26
7.26
7.25
7.25
7.24
7.24
7.23
7.18
7.18
7.17
6.83
6.82
6.81
6.80
6.61
6.60
5.35
5.34
4.43
4.42
4.42
4.41
3.95
3.94
3.94
3.93
3.93
3.73
3.71
3.66
3.66
3.65
3.64
3.63
3.34
3.34
3.13
2.96
2.92
2.56
2.51
2.50
2.50
2.50
2.49
1H (ppm)

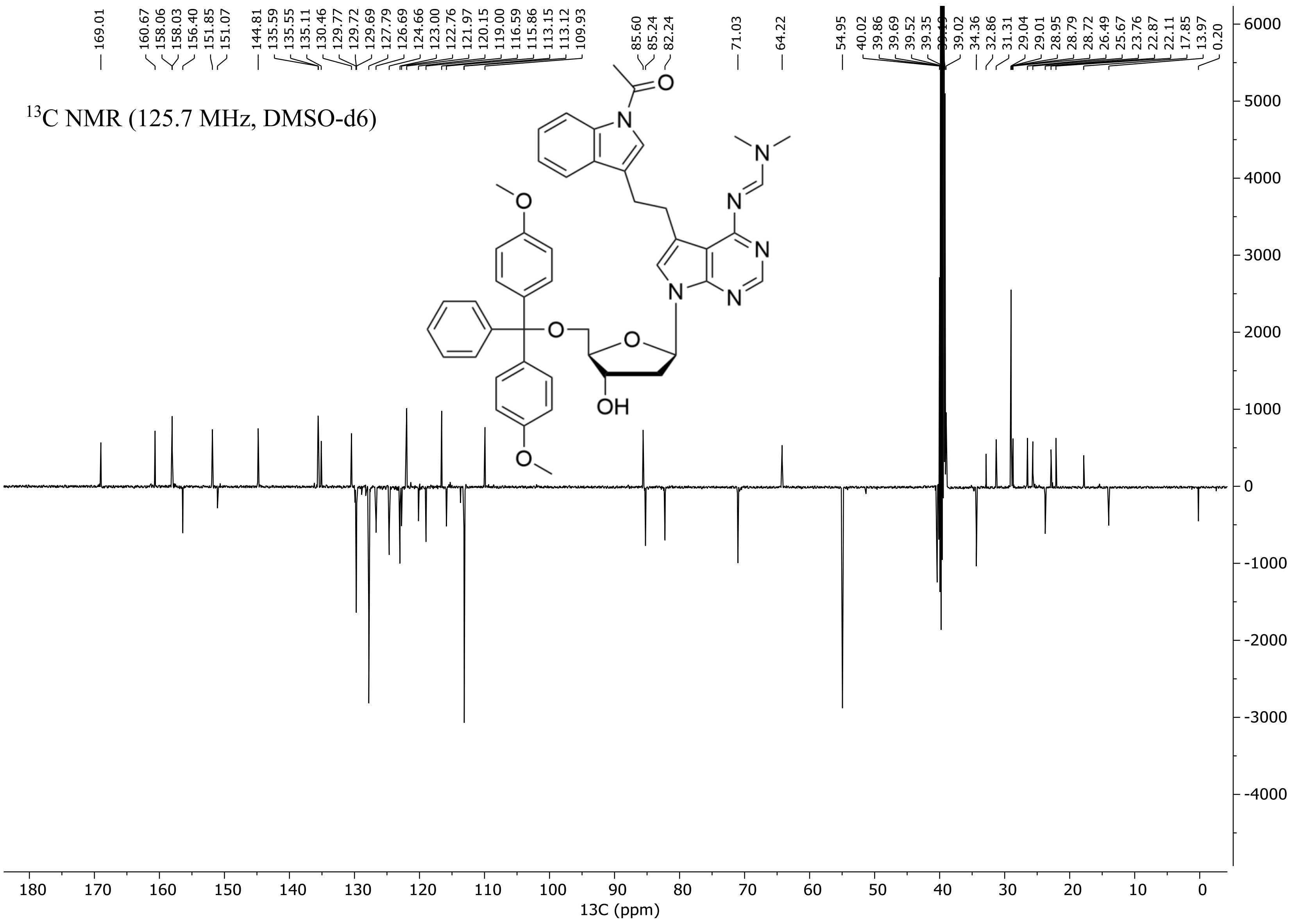

169.01
160.67
158.06
158.03
156.40
151.85
151.07
144.81
135.59
135.55
135.11
130.46
129.77
129.72
129.69
127.79
126.69
124.66
123.00
122.76
121.97
120.15
119.00
116.59
115.86
113.15
113.12
109.93
85.60
85.24
82.24
71.03
64.22
54.95
40.02
39.86
39.69
39.52
39.35
39.02
34.36
32.86
31.31
29.04
29.01
28.95
28.79
28.72
26.49
25.67
23.76
22.87
22.11
17.85
13.97
0.20
^{13}C NMR (125.7 MHz, DMSO-d6)
13C (ppm)

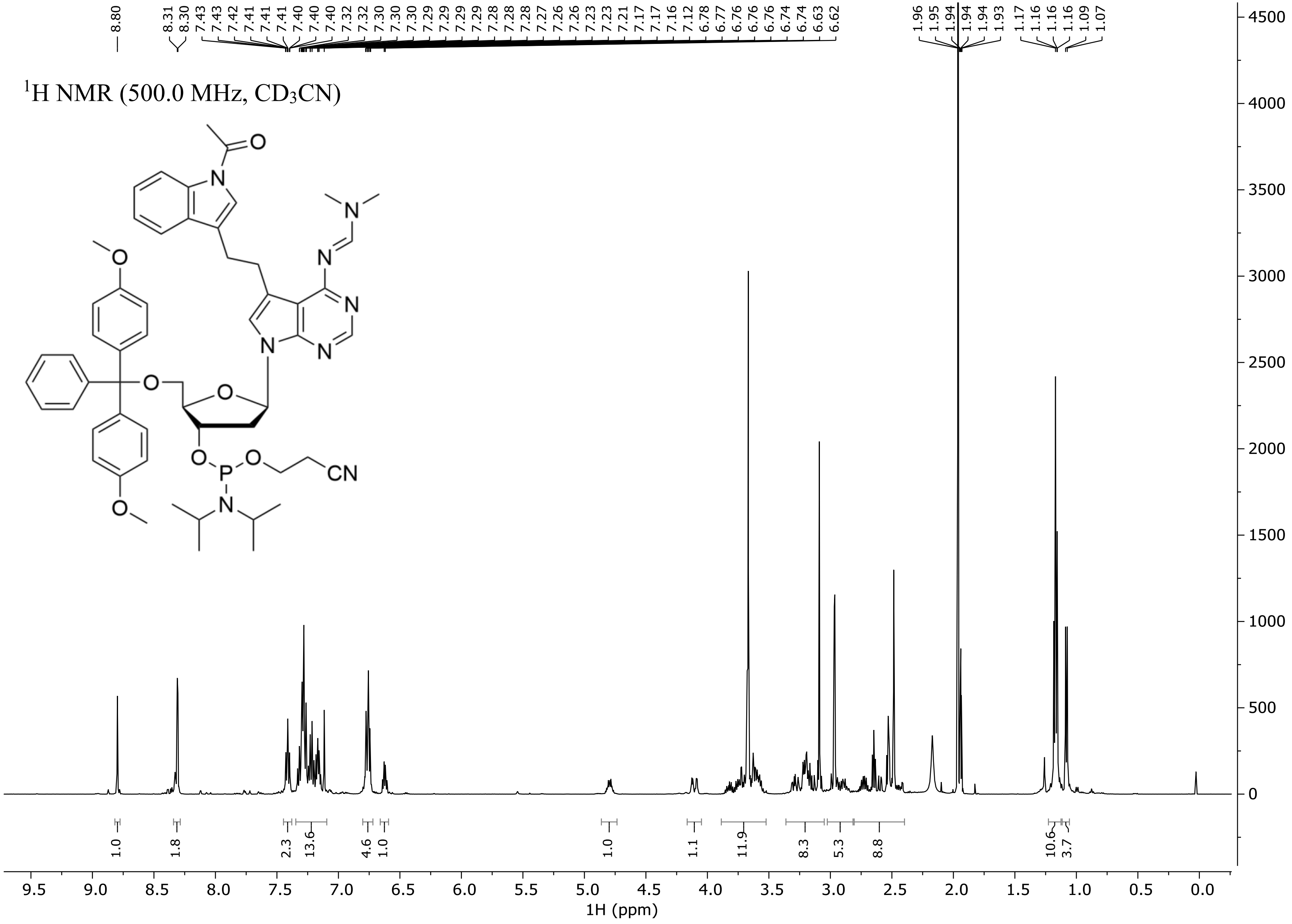
^{1}H NMR (500.0 MHz, CD_3CN)
1H (ppm)

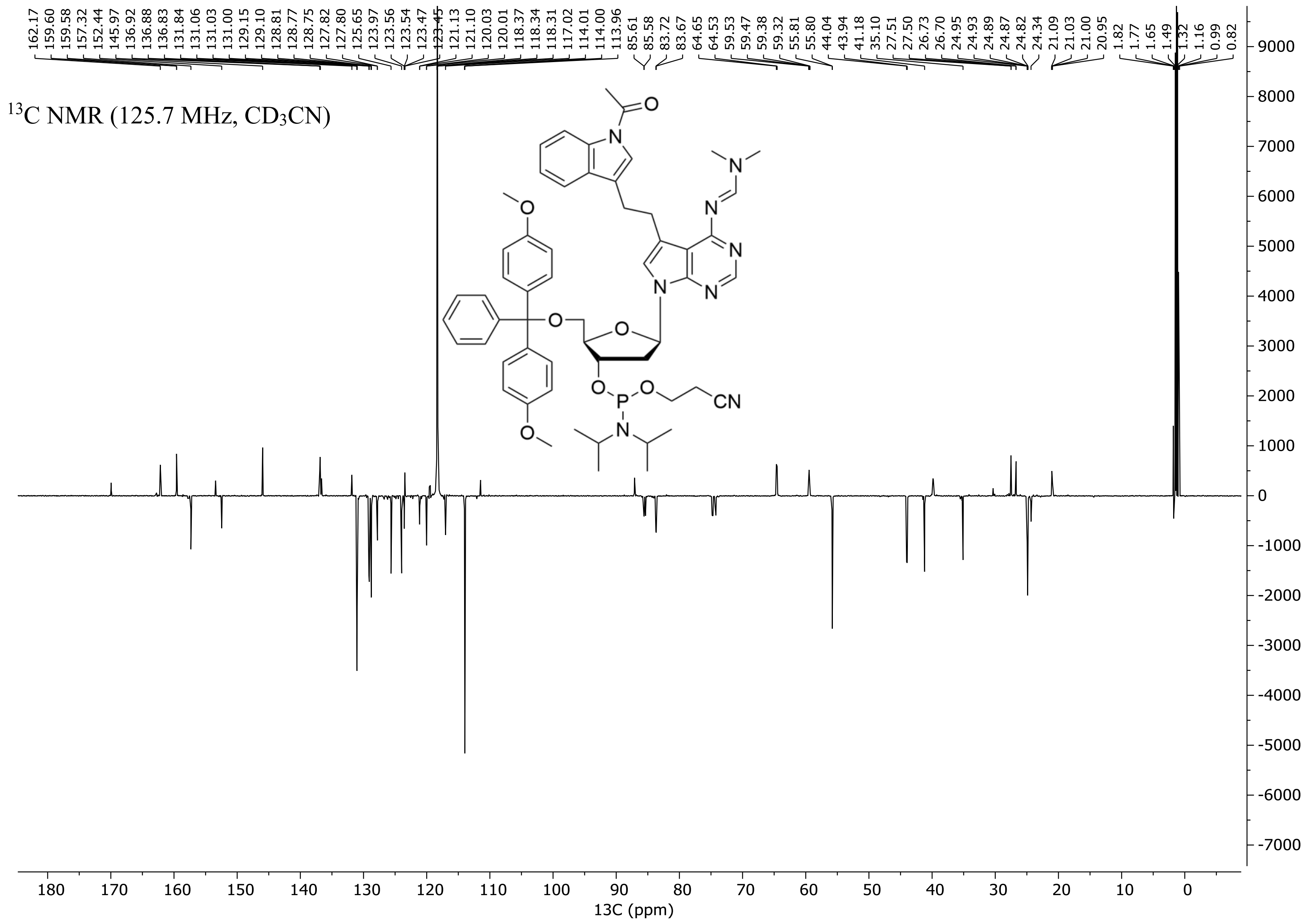

^{13}C NMR (125.7 MHz, CD$_3$CN)
162.17 159.60 159.58 157.32 152.44 145.97 136.92 136.88 136.83 131.84 131.06 131.03 131.00 129.15 129.10 128.81 128.77 128.75 127.82 127.80 125.65 123.97 123.56 123.54 123.47 123.45 121.13 121.10 120.03 120.01 118.37 118.34 118.31 117.02 114.01 114.00 113.96
85.61 85.58 83.72 83.67 64.65 64.53 59.53 59.47 59.38 59.32 55.81 55.80 44.04 43.94 41.18 35.10 27.51 27.50 26.73 26.70 24.95 24.93 24.89 24.87 24.82 24.34 21.09 21.03 21.00 20.95 1.82 1.77 1.65 1.49 1.32 1.16 0.99 0.82
13C (ppm)

$^{31}P\{^{1}H\}$ NMR (202.4 MHz, CD_3CN)

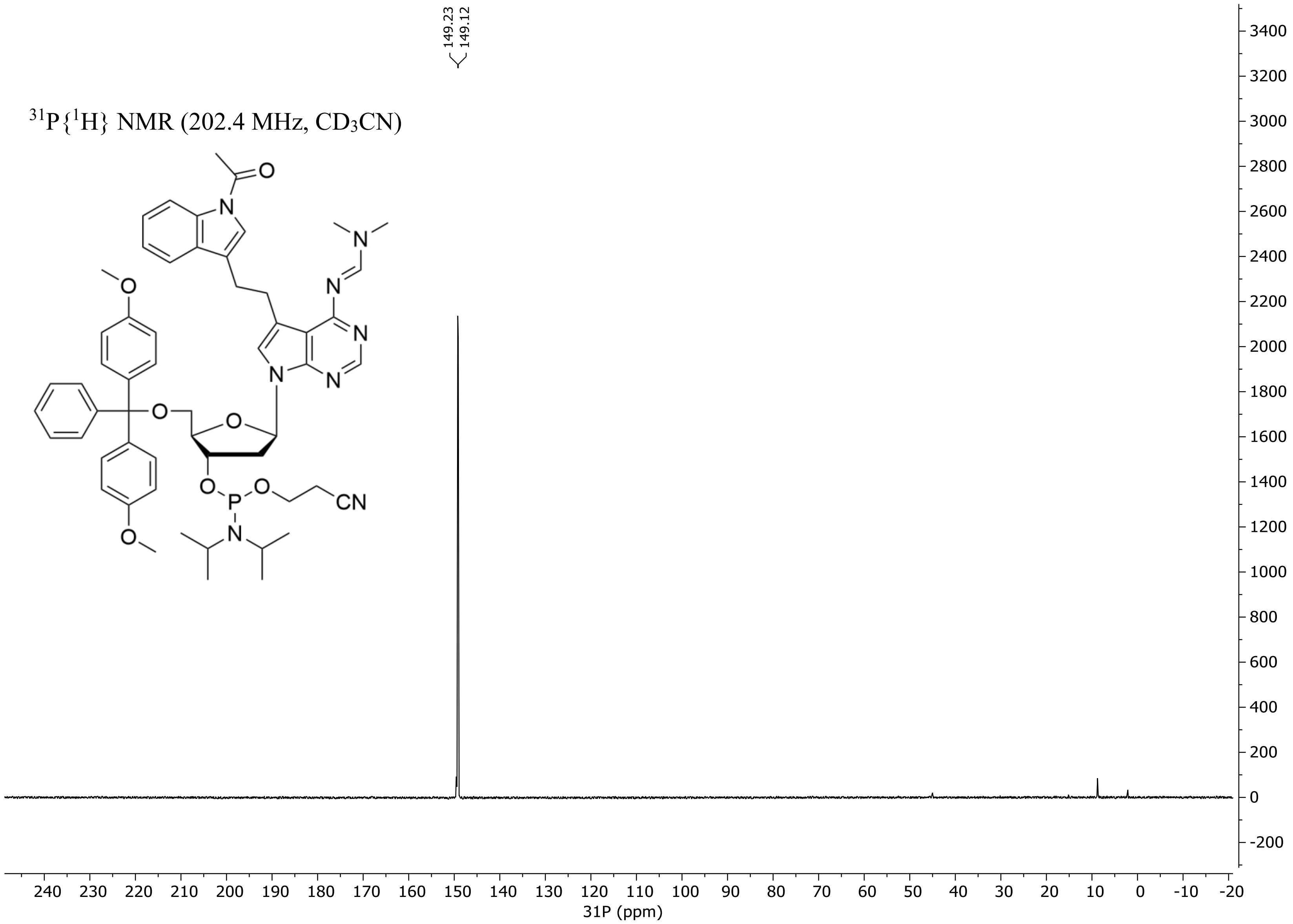

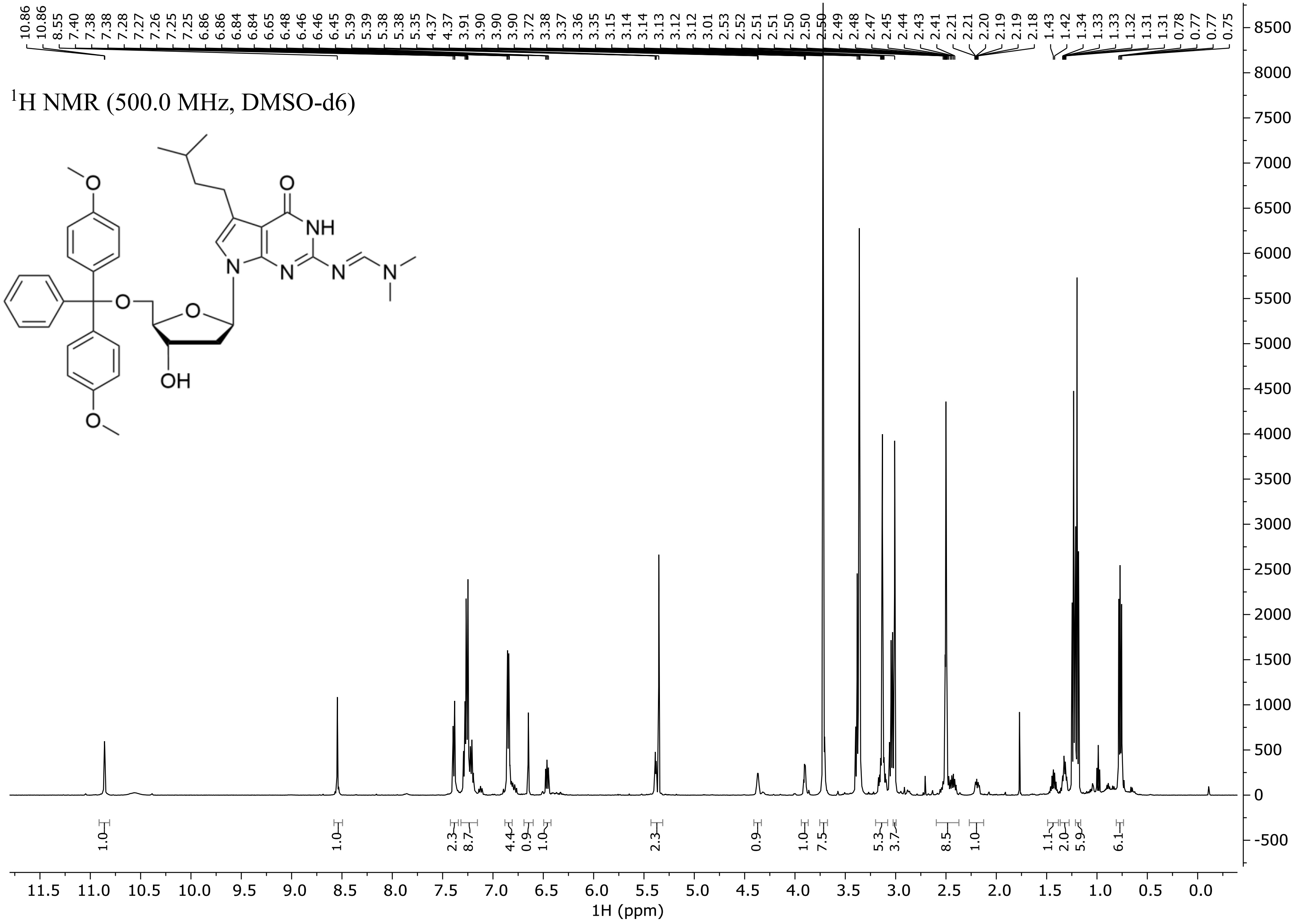
1H NMR (500.0 MHz, DMSO-d6)
1H (ppm)

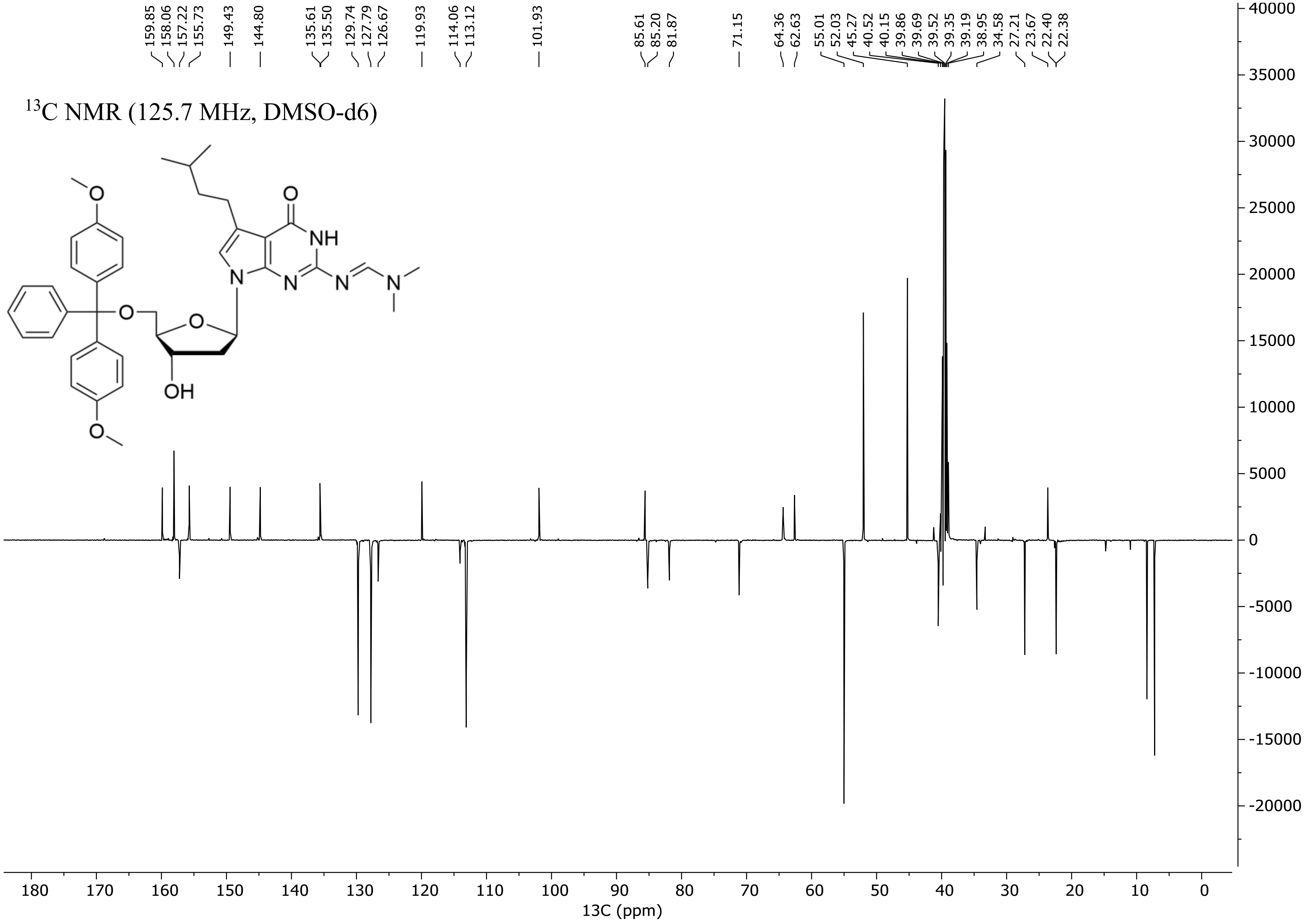
159.85
158.06
157.22
155.73
149.43
144.80
135.61
135.50
129.74
127.79
126.67
119.93
114.06
113.12
101.93
85.61
85.20
81.87
71.15
64.36
62.63
55.01
52.03
45.27
40.52
40.15
39.86
39.69
39.52
39.35
39.19
38.95
34.58
27.21
23.67
22.40
22.38
13C NMR (125.7 MHz, DMSO-d6)
O
NH
N
N
N
N
O
O
O
OH
O
13C (ppm)

^{1}H NMR (500.0 MHz, CD_3CN)

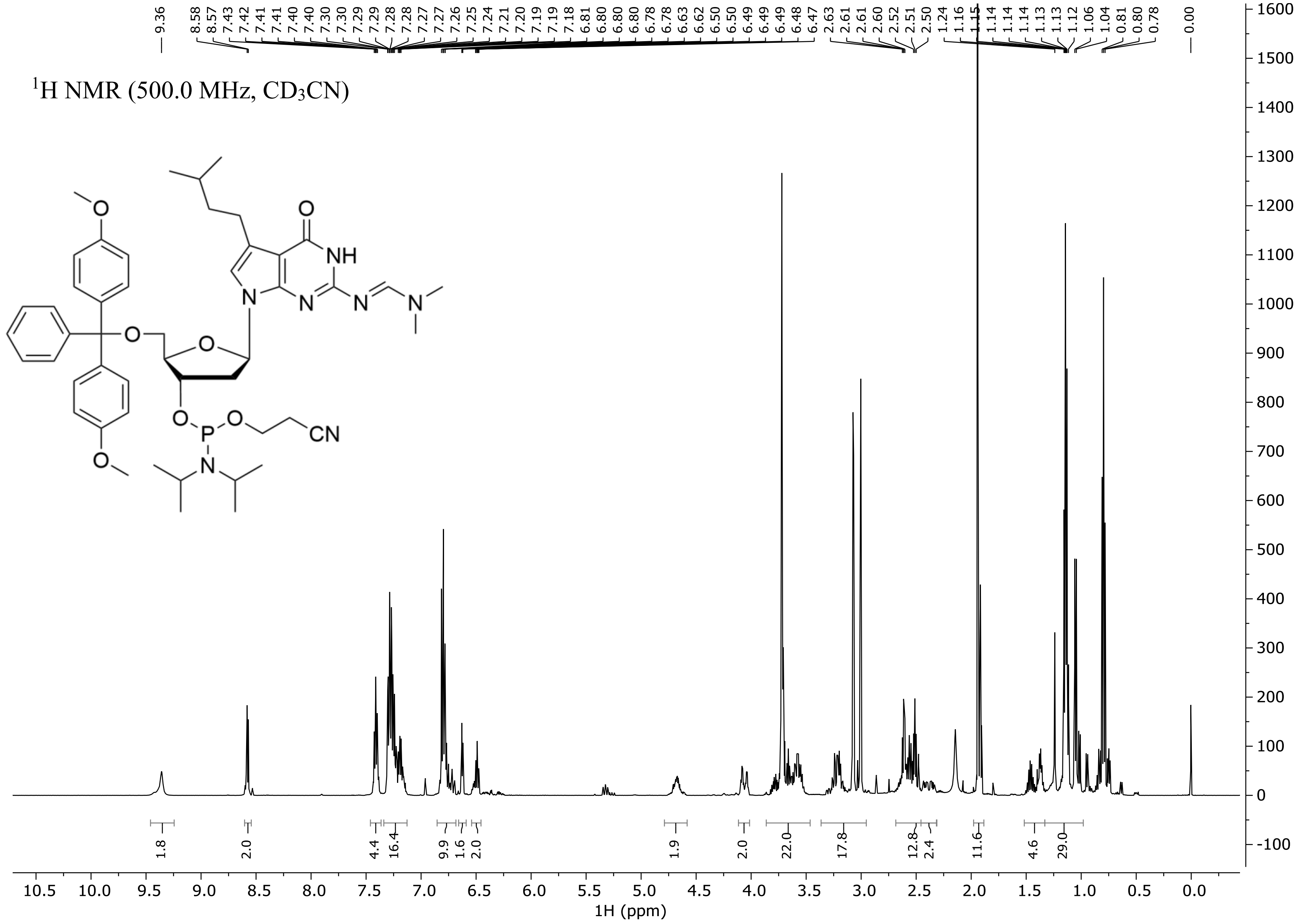

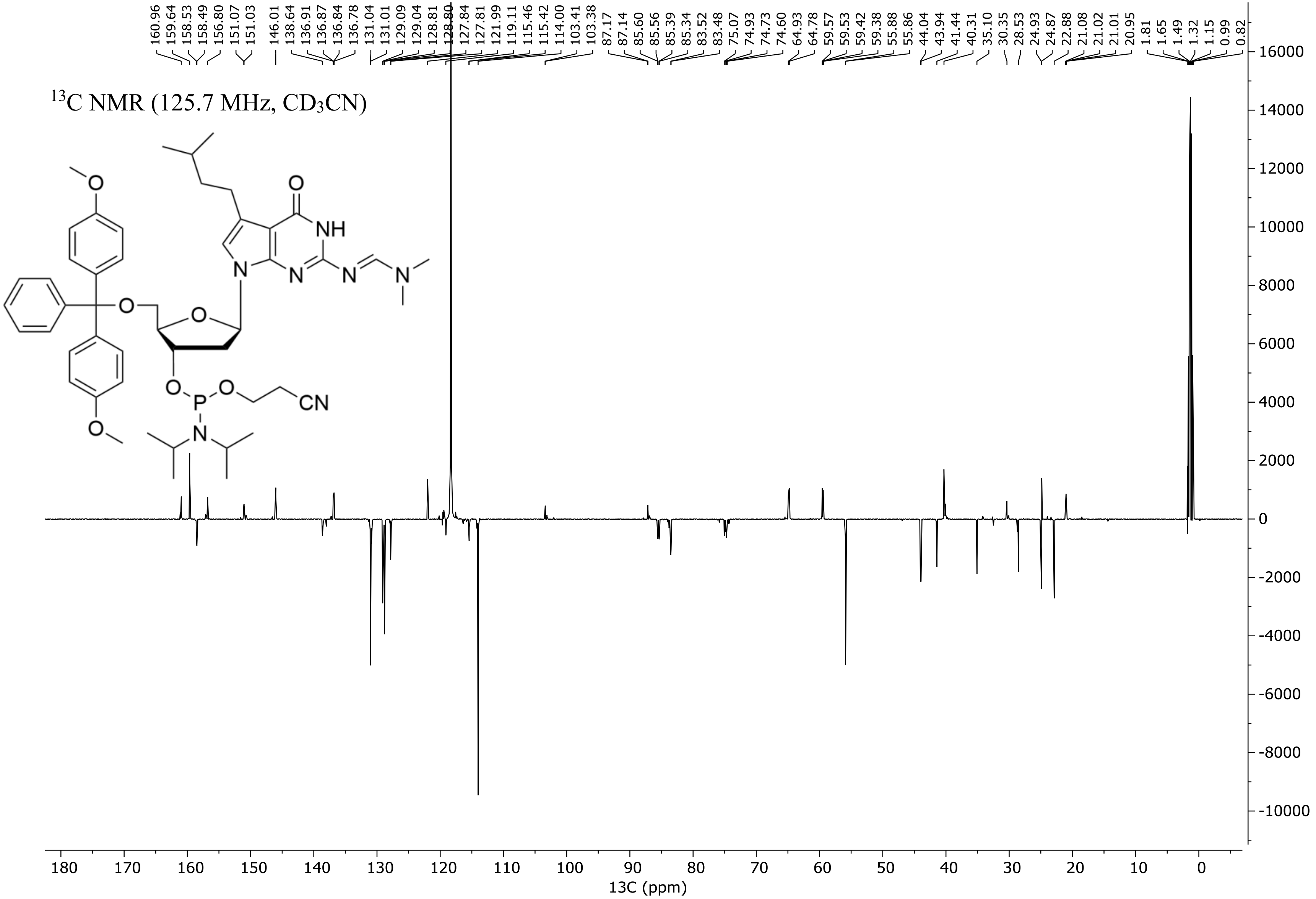
13C NMR (125.7 MHz, CD3CN)
160.96 159.64 158.53 158.49 156.80 151.07 151.03 146.01 138.64 136.91 136.87 136.84 136.78 131.04 131.01 129.09 129.04 128.81 128.80 127.84 127.81 121.99 119.11 115.46 115.42 114.00 103.41 103.38 87.17 87.14 85.60 85.56 85.39 85.34 83.52 83.48 75.07 74.93 74.73 74.60 64.93 64.78 59.57 59.53 59.42 59.38 55.88 55.86 44.04 43.94 41.44 40.31 35.10 30.35 28.53 24.93 24.87 22.88 21.08 21.02 21.01 20.95 1.81 1.65 1.49 1.32 1.15 0.99 0.82
O
NH
N
N
N
N
O
O
O
O
O
P
O
CN
N
13C (ppm)

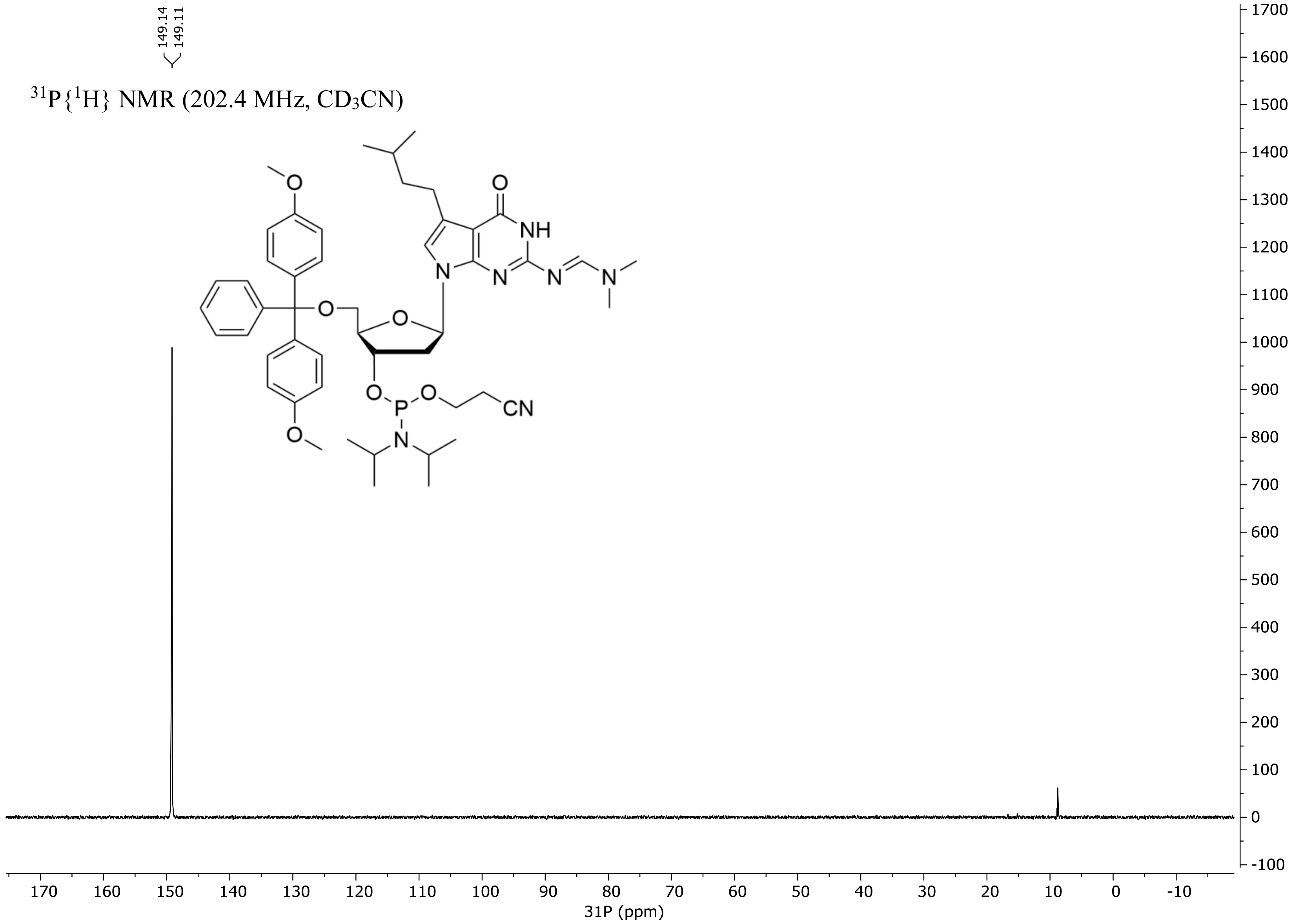
149.14
149.11
$^{31}P\{^{1}H\}$ NMR (202.4 MHz, CD_3CN)
O
NH
N
N
N
N
O
O
O
P
N
O
CN
O
O
31P (ppm)

4 Reference List

1. B. D. Marty, A. J. Baldwin, E. G. Marklund, N. Hochberg, J. L. P. Benesch and C. V. Robinson, *Anal. Chem.*, **2015**, **87**, 4370–4376.
2. I. Jestřábová, L. Poštová Slavětínská and M. Hocek, *ACS Omega*, **2023**, **8**, 39447–39453.
3. L. A. Matarazzo, J. M. Brow and R. H. Hudson, *Can. J. Chem.*, **2018**, **96**, 1093–1100.
4. M. Ondruš, V. Sýkorová, L. Bednárová, R. Pohl and M. Hocek, *Nucleic Acids Res.*, **2020**, **48**, 11982–11993.
5. X. Zhang, H. Wang, J. Chen, Y. Zhang, L. Gu and B. Xiao, *Angew. Chem. Int. Ed.*, **2011**, **50**, 1896–1900.
6. H. Iida, T. Ishida, Y. Watanabe and S. Arai, *J. Org. Chem.*, **2019**, **84**, 7411–7417.
7. F. Sánchez-Cantalejo, J. D. Priest and P. W. Davies, *Chem. Eur. J.*, **2018**, **24**, 17215–17219.
8. F. Seela and X. Peng, *Collect. Czech. Chem. Commun.*, **2006**, **71**, 1401–1424.
9. S. Knapp, S. Serva, J. D'Onofrio, W. Keller, L. Lubys, R. Kurg, M. Remm and J. W. Engels, *Chem. Eur. J.*, **2011**, **17**, 2903–2915.
10. F. Seela and M. Zulauf, *Chem. Eur. J.*, **1998**, **4**, 1781–1790.
11. F. Seela and X. Peng, *Synthesis*, **2004**, 1203–1210.
